# Principles of mRNA targeting via the *Arabidopsis* m^6^A-binding protein ECT2

**DOI:** 10.7554/eLife.72375

**Published:** 2021-09-30

**Authors:** Laura Arribas-Hernández, Sarah Rennie, Tino Köster, Carlotta Porcelli, Martin Lewinski, Dorothee Staiger, Robin Andersson, Peter Brodersen

**Affiliations:** 1 University of Copenhagen, Copenhagen Plant Science Center Copenhagen N Denmark; 2 Department of Biology, University of Copenhagen Copenhagen Denmark; 3 University of Bielefeld, Faculty of Biology, RNA Biology and Molecular Physiology Bielefeld Germany; Universidad Nacional del Litoral-CONICET Argentina; University of Freiburg Germany

**Keywords:** m6A, ECT2, iCLIP, hyperTRIBE, RRACH, motif, GGAU, URUAY, target, *A. thaliana*

## Abstract

Specific recognition of *N6*-methyladenosine (m^6^A) in mRNA by RNA-binding proteins containing a YT521-B homology (YTH) domain is important in eukaryotic gene regulation. The *Arabidopsis* YTH domain protein ECT2 is thought to bind to mRNA at URU(m^6^A)Y sites, yet RR(m^6^A)CH is the canonical m^6^A consensus site in all eukaryotes and ECT2 functions require m^6^A-binding activity. Here, we apply iCLIP (*i*ndividual nucleotide resolution *c*ross*l*inking and *i*mmuno*p*recipitation) and HyperTRIBE (*t*argets of *R*NA-binding proteins *i*dentified *b*y *e*diting) to define high-quality target sets of ECT2 and analyze the patterns of enriched sequence motifs around ECT2 crosslink sites. Our analyses show that ECT2 does in fact bind to RR(m^6^A)CH. Pyrimidine-rich motifs are enriched around, but not at m^6^A sites, reflecting a preference for *N6*-adenosine methylation of RRACH/GGAU islands in pyrimidine-rich regions. Such motifs, particularly oligo-U and UNUNU upstream of m^6^A sites, are also implicated in ECT2 binding via its intrinsically disordered region (IDR). Finally, URUAY-type motifs are enriched at ECT2 crosslink sites, but their distinct properties suggest function as sites of competition between binding of ECT2 and as yet unidentified RNA-binding proteins. Our study provides coherence between genetic and molecular studies of m^6^A-YTH function in plants and reveals new insight into the mode of RNA recognition by YTH domain-containing proteins.

## eLife digest

Genes are strings of genetic code that contain instructions for producing a cell’s proteins. Active genes are copied from DNA into molecules called mRNAs, and mRNA molecules are subsequently translated to create new proteins. However, the number of proteins produced by a cell is not only limited by the number of mRNA molecules produced by copying DNA. Cells use a variety of methods to control the stability of mRNA molecules and their translation efficiency to regulate protein production. One of these methods involves adding a chemical tag, a methyl group, onto mRNA while it is being created. These methyl tags can then be used as docking stations by RNA-binding proteins that help regulate protein translation.

Most eukaryotic species – which include animals, plants and fungi – use the same system to add methyl tags to mRNA molecules. One methyl tag in particular, known as m^6^A, is a well-characterised docking site for a particular type of RNA-binding protein that goes by the name of ECT2 in plants. However, in the flowering plant *Arabidopsis thaliana*, ECT2 was thought to bind to an mRNA sequence different from the one normally carrying the chemical tag, creating obvious confusion about how the system works in plants.

Arribas-Hernández, Rennie et al. investigated this question using advanced large-scale biochemical techniques, and discovered that conventional m^6^A methyl tags are indeed used by ECT2 in *Arabidopsis thaliana*. The confusion likely arose because the sequence ECT2 was thought bind is often located in close proximity to the m^6^A tags, possibly acting as docking stations for proteins that can influence the ability of ECT2 to bind mRNA. Arribas-Hernández, Rennie et al. also uncovered additional mRNA sequences that directly interact with parts of ECT2 previously unknown to participate in mRNA binding.

These findings provide new insights into how chemical labels in mRNA control gene activity. They have broad implications that extend beyond plants into other eukaryotic species, including humans. Since this chemical labelling system has a major role in controlling plant growth, these findings could be leveraged in biotechnology applications to improve crop yields and enhance plant-based food production.

## Introduction

*N6*-methyladenosine (m^6^A) is the most abundant modified nucleotide in eukaryotic mRNA bodies. It is required for embryonic development and stem cell differentiation in several animals and plants (Zhong et al., 2008; Batista et al., 2014; Ping et al., 2014; Geula et al., 2015; Zhang et al., 2017) and for the control of the meiotic program in yeast (Shah and Clancy, 1992; Clancy et al., 2002; Agarwala et al., 2012). Most *N6*-adenosine methylation of mRNA is catalyzed in the nucleus (Salditt-Georgieff et al., 1976; Ke et al., 2017; Huang et al., 2019) by a highly conserved, multimeric methylase (the m^6^A ‘writer’; Balacco and Soller, 2019) whose catalytic core consists of the heterodimer METTL3/METTL14 (MTA/MTB in plants; Bokar et al., 1997; Zhong et al., 2008; Liu et al., 2014). In addition, a number of highly conserved proteins is required for *N6*-methylation in vivo (Balacco and Soller, 2019). The strong conservation of these core factors suggests that the biochemical basis of *N6*-adenosine methylation is common in eukaryotes, and indeed, m^6^A occurs in the consensus site RR(m^6^A)CH (R = G/A, H = A/C/U), primarily in 3′-UTRs in vertebrates, plants, and fungi that possess the canonical METTL3/METTL14 methyltransferase (Bodi et al., 2012; Dominissini et al., 2012; Meyer et al., 2012; Schwartz et al., 2013; Luo et al., 2014a; Zhao et al., 2017; Miao et al., 2020; Parker et al., 2020). Conversely, the characteristic motif and gene body location is not detected in organisms that lack METTL3/METTL14 homologs, such as the nematode *Caenorhabditis elegans* (Sendinc et al., 2020) and bacteria (Deng et al., 2015).

m^6^A may impact mRNA function by different mechanisms, including the creation of binding sites for reader proteins that specifically recognize m^6^A in mRNA (Dominissini et al., 2012; Fu et al., 2014; Meyer and Jaffrey, 2014). The best understood class of readers contains a so-called YT521-B homology (YTH) domain (Stoilov et al., 2002) of which two phylogenetic groups, YTHDF and YTHDC, have been defined (Patil et al., 2018; Balacco and Soller, 2019). The YTH domain harbors a hydrophobic methyl-binding pocket that increases the affinity of m^6^A-containing RNA by more than 10-fold compared to unmethylated RNA (Li et al., 2014b; Luo and Tong, 2014b; Theler et al., 2014; Xu et al., 2014; Zhu et al., 2014). Apart from interactions with the methylated adenosine and the purine at the –1 position, YTH domain-RNA interactions mostly involve the sugar-phosphate backbone of the RNA (Luo and Tong, 2014b; Theler et al., 2014; Xu et al., 2014). That is consistent with only mild reductions in the binding affinity of the YTH domain of human YTHDC1 upon substitution of nucleotides −2, +1, and +3 that abrogate the canonical RR(m^6^A)CH motif (Xu et al., 2014), and poor sequence specificity of RNA binding by isolated YTH domains of human YTHDF1, YTHDF2, and YTHDC1 (Arguello et al., 2019). Thus, the methyltransferase complex gives the sequence specificity, while YTH domain proteins may bind to m^6^A-containing RNA regardless of the identity of the immediately adjacent nucleotides.

YTHDF proteins are typically cytoplasmic and consist of a long N-terminal intrinsically disordered region (IDR) followed by the globular YTH domain (Patil et al., 2018). Because the affinity of isolated YTH domains for m^6^A-containing RNA is modest, typically with dissociation constants on the order of 0.1–1 μM (Li et al., 2014b; Luo and Tong, 2014b; Theler et al., 2014; Xu et al., 2014; Zhu et al., 2014), it has been suggested that the IDR may participate in RNA binding (Patil et al., 2018). Nonetheless, the clearest evidence for functions of the IDRs in YTHDF proteins reported thus far includes direct interactions with effectors such as the CCR4-NOT complex in mammalian cells (Du et al., 2016), and the ability to cause liquid-liquid phase transition when sufficiently high local concentrations are reached (Arribas-Hernández et al., 2018; Gao et al., 2019; Ries et al., 2019; Fu and Zhuang, 2020; Wang et al., 2020).

The YTHDF family comprises 11 proteins in *Arabidopsis* that are referred to as EVOLUTIONARILY CONSERVED C-TERMINAL REGION1-11 (ECT1-11) (Li et al., 2014c; Scutenaire et al., 2018). ECT2, ECT3, and ECT4 are expressed in rapidly dividing cells of root, leaf, and flower primordia, and genetic analyses have revealed their general importance in organogenesis (Arribas-Hernández et al., 2018; Arribas-Hernández et al., 2020). Importantly, the biological functions of ECT2/ECT3/ECT4 described thus far are shared with those of m^6^A writer components and, where tested, have been shown to depend on intact m^6^A-binding pockets, strongly suggesting that the basis for the observed phenotypes in *ect2/ect3/ect4* mutants is defective regulation of m^6^A-modified mRNA targets (Bodi et al., 2012; Shen et al., 2016; Ružička et al., 2017; Arribas-Hernández et al., 2018; Scutenaire et al., 2018; Wei et al., 2018; Arribas-Hernández et al., 2020). Despite the progress in identifying biological functions of plant m^6^A-YTHDF axes, a number of fundamental questions regarding their molecular basis remains unanswered. For example, it is unclear whether sequence determinants in addition to m^6^A are important for mRNA target association of ECT proteins in vivo, the mRNA targets of ECT2/ECT3/ECT4 responsible for the developmental delay of *ect2*/*ect3/*(*ect4*) mutants have not been identified, and it is not clear what the effects of ECT2/ECT3/ECT4 binding to them may be (Arribas-Hernández and Brodersen, 2020). Clearly, robust identification of the mRNA targets directly bound by ECT proteins is key to obtain satisfactory answers to all of these questions. Towards that goal, formaldehyde crosslinking and immunoprecipitation (FA-CLIP) was used to identify mRNA targets of ECT2 (Wei et al., 2018). Nonetheless, because formaldehyde, in contrast to UV illumination, generates both protein-protein and protein-RNA crosslinks, it is not an ideal choice for identification of mRNAs bound directly by a protein of interest (see Arribas-Hernández and Brodersen, 2020 for a discussion). In particular, this problem concerns the unexpected conclusion that ECT2 binds to the ‘plant-specific consensus motif’ URU(m^6^A)Y (Y = U/C), not RR(m^6^A)CH (Wei et al., 2018). Thus, the field of gene regulation via m^6^A-YTHDF modules in plants is in a state of confusion: on the one hand, m^6^A mapping (Luo et al., 2014a; Wan et al., 2015; Shen et al., 2016; Duan et al., 2017; Anderson et al., 2018; Miao et al., 2020; Parker et al., 2020) and phenotypes of mutants defective in m^6^A writing (Bodi et al., 2012; Shen et al., 2016; Ružička et al., 2017) or m^6^A binding of ECT2/ECT3/ECT4 (Arribas-Hernández et al., 2018; Arribas-Hernández et al., 2020) suggest that these YTHDF proteins should act via recognition of m^6^A in the RRACH context. On the other hand, the only attempt at a mechanistic understanding of ECT2 function via mRNA target identification concluded that ECT2 binds to a sequence element different from RRACH (Wei et al., 2018). To complicate matters further, a number of motifs including not only URUAY, but also UGUAMM (M = A/C), UGWAMH (W = A/U), UGUAWA, and GGAU have been reported to be enriched around m^6^A sites in *Arabidopsis* and other plant species (Li et al., 2014a; Anderson et al., 2018; Miao et al., 2020; Zhang et al., 2019; Zhou et al., 2019), but it remains unclear whether the adenosines in such motifs are methylated in vivo. Alternatively, these sequence contexts may play a role in guiding m^6^A deposition or ECT recognition nearby, either directly by ECT interaction or indirectly via additional RNA-binding proteins assisting or competing with ECT binding.

To clarify principles underlying mRNA recognition by ECT2, we undertook rigorous analysis of its mRNA-binding sites using two orthogonal methods, the proximity-labeling method HyperTRIBE (*t*argets of *R*NA-binding proteins *i*dentified *b*y *e*diting) (McMahon Aoife et al., 2016; Xu et al., 2018) and iCLIP (*i*ndividual nucleotide resolution *c*ross*l*inking and *i*mmuno*p*recipitation) (König et al., 2010). This resulted in identification of high-quality target sets as judged by mutual overlaps and by overlaps with previously reported m^6^A maps from plants at a similar developmental stage (Shen et al., 2016; Parker et al., 2020). Relying on this high-quality target set, we used the position information inherent to iCLIP and a single-nucleotide resolution m^6^A dataset (Parker et al., 2020) to establish six properties of m^6^A-containing mRNA and mRNA targeting by ECT2. (1) RRACH and its variant DRACH (D = R/U) are unequivocally the most highly enriched motifs at m^6^A sites in *Arabidopsis*. (2) ECT2 binds to m^6^A sites in the canonical RRACH context as ECT2 crosslinking sites are preferentially found immediately 5′ to m^6^A sites, and RRACH is enriched immediately 3′ to ECT2 crosslinking sites. (3) GGAU is a minor m^6^A consensus site in plants. (4) U- and U/C-rich motifs are enriched around, but not at, m^6^A sites, and, together with RRACH and GGAU, constitute core elements that distinguish m^6^A-containing 3′-UTRs from non-m^6^A-containing 3′-UTRs in plants. (5) The IDR of ECT2 participates in RNA binding as it crosslinks to target mRNAs at U-rich elements highly abundant upstream of m^6^A sites. (6) Although URUAY, URURU, and similar motifs may crosslink to ECT2, their presence in m^6^A-containing mRNA disfavors ECT2 binding, consistent with those motifs acting predominantly as sites of interaction for RNA-binding proteins that may compete with ECT2.

## Results

### ADARcd fusions to ECT2 are functional in vivo

**Figure 1.**
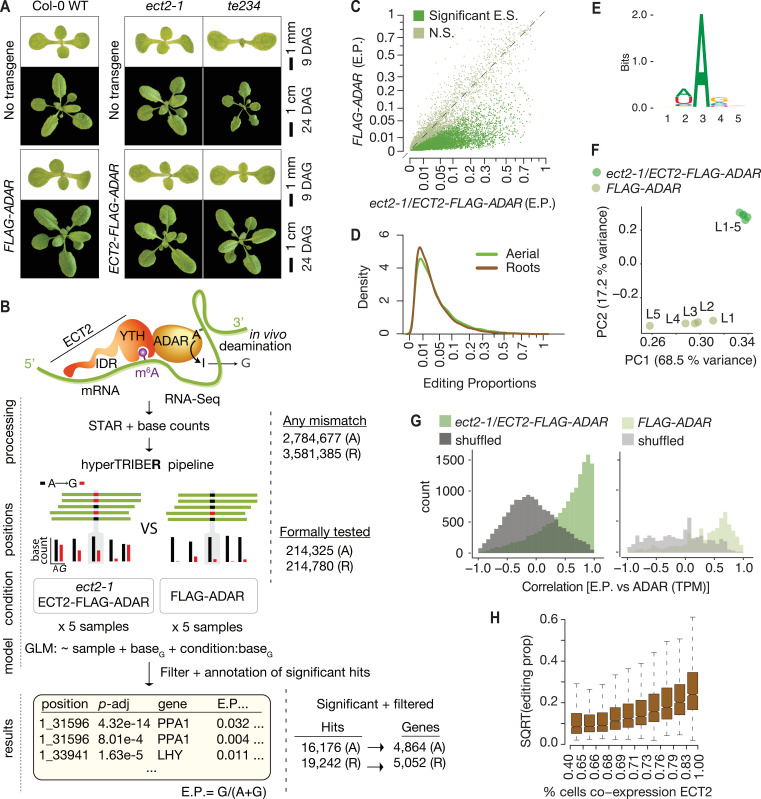
*Drosophila* ADARcd fused to ECT2 can edit target mRNAs in vivo in plants. (**A**) Phenotypes of wild type, *ect2-1* and *te234* mutants with (lower panels) or without (upper panels) *ECT2-FLAG-ADAR* or *FLAG-ADAR* transgenes, at 9 or 24 days after germination (DAG). (**B**) Experimental design for ECT2-HyperTRIBE (ECT2-HT) target identification and hyperTRIBE**R** pipeline (Rennie et al., 2021). Nucleotide base counts quantified from mapped RNA-seq libraries were passed into the hyperTRIBE**R** pipeline to call significant editing sites, which were further filtered and annotated. The number of sites in either aerial (A, dissected apices) or root (R, root tips) tissues considered at each stage of the analysis is indicated. GLM, generalized linear model; E.P., editing proportion. (**C**) Scatterplot of the editing proportions of potential and significant editing sites (E.S.) in aerial tissues of *ect2-1/ECT2-FLAG-ADAR* lines compared to the *FLAG-ADAR* controls. Significant sites are highlighted in vivid green. N.S., not significant. (**D**) Density of editing proportions for significant editing sites in aerial tissues and roots of *ect2-1/ECT2-FLAG-ADAR* lines. (**E**) Consensus motif identified at significant editing sites in aerial tissues of *ect2-1/ECT2-FLAG-ADAR* lines. (**F**) Principal component analysis of editing proportions at significant editing sites in samples with aerial tissues. (**G**) Distribution of the correlations between editing proportions and ADAR expression (TPM) for significant editing sites in aerial tissues of either *ect2-1/ECT2-FLAG-ADAR* or *FLAG-ADAR* lines. Background correlations (gray) are based on randomly shuffling ADAR expression for each site. (**H**) Boxplots showing the mean editing proportions as a function of the proportion of cells co-expressing *ECT2*, calculated based on single cell RNA-seq in roots (Denyer et al., 2019). For panels **C, E, F**, and **G**, comparable analyses in both aerial and root tissues are shown in the Figure 1—figure supplement 1.

HyperTRIBE uses fusion of RNA-binding proteins to the hyperactive E488Q mutant of the catalytic domain of the *Drosophila melanogaster* adenosine deaminase acting on RNA (*Dm*ADAR^E488Q^cd) (Kuttan and Bass, 2012) to achieve proximity labeling in vivo (McMahon Aoife et al., 2016; Xu et al., 2018). Targets are identified as those mRNAs that contain adenosine-inosine sites significantly more highly edited than background controls, measured as A-G changes upon reverse transcription and sequencing. To develop material suitable for ECT2 HyperTRIBE, we expressed *AtECT2pro:AtECT2-FLAG-DmADAR^E488Q^cd-AtECT2ter* (henceforth ‘*ECT2-FLAG-ADAR’*) in the single *ect2-1* and triple *ect2-1/ect3-1/ect4-2* (*te234*) knockout backgrounds (Arribas-Hernández et al., 2018; Arribas-Hernández et al., 2020). We identified lines exhibiting nearly complete rescue of *te234* mutant seedling phenotypes, indicating that the fusion protein was functional (Figure 1A). We then used the expression level in complementing lines as a criterion to select lines in the *ect2-1* single mutant background, for which no easily scorable phenotype has been described (Figure 1—figure supplement 1A). Lines expressing free *Dm*ADAR^E488Q^cd under the control of the endogenous *ECT2* promoter (*AtECT2pro:FLAG-DmADAR^E488Q^cd-AtECT2ter;* henceforth *FLAG-ADAR*) at levels similar to or higher than those of the fusion lines (Figure 1—figure supplement 1A,B) were used to control for background editing after verification that *FLAG-ADAR* expression did not result in phenotypic abnormalities in Col-0 WT plants (Figure 1A).

**Figure 1—figure supplement 1.**
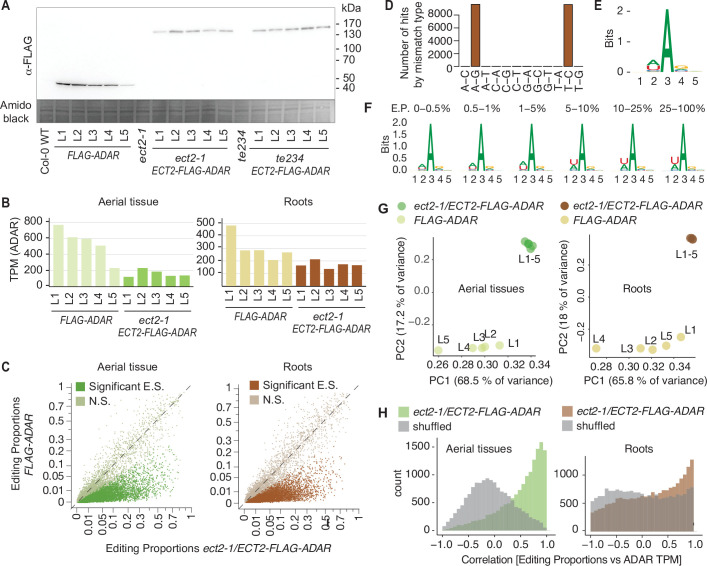
*Drosophila* ADARcd fused to ECT2 can edit target mRNAs in vivo in plants (extended data, aerial and root tissues). (**A**) Protein expression levels of *ECT2pro:ECT2-FLAG-DmADAR^E488Q^cd-ECT2ter* (*ECT2-FLAG-ADAR*) or *ECT2pro:FLAG-DmADAR^E488Q^cd-ECT2ter* (*FLAG-ADAR*) transgenes in 10-day-old seedlings of five independent transgenic lines (L1–L5) of each of the genotypes shown in Figure 1A and their background controls. Amido black staining is used as loading control. (**B**) mRNA expression levels (TPM) of *ECT2-FLAG-ADAR* or *FLAG-ADAR* in dissected apices (aerial tissues) or root tips of the lines used for ECT2-HyperTRIBE (ECT2-HT). (**C**) Scatterplot of the editing proportions, defined as G/(A + G), of potential and significant editing sites (E.S.) in aerial and root tissues of *ect2-1/ECT2-FLAG-ADAR* lines compared to the *FLAG-ADAR* controls. Significant sites are highlighted in vivid colors. N.S., not significant. (**D**) Number of significant hits in root samples by mismatch type, after filtering but before removing non-A-G or non-T-C sites. (**E, F**) Consensus motif identified at significant editing sites in roots of *ect2-1/ECT2-FLAG-ADAR* lines, split in groups by editing proportions (E.P.) in (**F**). (**G**) Principal component analysis of editing proportions for significant editing sites. (**H**) Distribution of the correlations between editing proportions and ADAR expression (TPM) for significant editing sites. Background correlations (gray) are based on randomly shuffling ADAR expression for each site. Figure 1—figure supplement 1—source data 1.Uncropped labeled panels and raw image files: Figure 1—figure supplement 1A.

### The ECT2-ADARcd fusion imparts adenosine-to-inosine editing of target mRNAs in planta

To identify ECT2 HyperTRIBE targets (HT-targets), we sequenced mRNA from dissected root tips and shoot apices of 10-day-old seedlings of *ect2-1*/*ECT2-FLAG-ADAR* and *FLAG-ADAR* transgenic lines using five independent lines of each type as biological replicates to prevent line-specific artifacts. Next, we generated nucleotide base counts for all positions with at least one mismatch across the full set of samples of mapped reads (Figure 1B), resulting in a raw list of potential editing positions. This revealed that the amount of editing was clearly higher in the lines expressing the ECT2-FLAG-ADAR fusion protein than in the negative control lines (Figure 1C, Figure 1—figure supplement 1C). To identify positions with significantly higher editing rates in ECT2-FLAG-ADAR lines compared to controls, we developed a new approach to detect differential editing (Figure 1B) described in detail by Rennie et al., 2021. Briefly, the hyperTRIBE**R** method of detecting differential editing exploits the powerful statistical capabilities of a method originally designed to detect differential exon usage (Anders et al., 2012). It efficiently takes replicates and possible differences in expression into account, resulting in high power to detect sites despite the generally low editing proportions that we found in our data (Figure 1D). As expected, the tendency towards higher editing proportions in fusion lines compared to controls was even more pronounced after filtering nonsignificantly edited sites (Figure 1C, Figure 1—figure supplement 1C). Three additional properties of the resulting editing sites indicate that they are the result of ADARcd activity guided by its fusion to ECT2. First, the vast majority of significant hits corresponded to A-to-G transitions (Figure 1—figure supplement 1D). Second, the consensus motif at the edited sites matched the sequence preference of *Dm*ADAR^E488Q^cd (5′ and 3′ nearest-neighbor preference of U>A>C>G and G>C>A~U, respectively [Xu et al., 2018; Figure 1E, Figure 1—figure supplement 1E]), with highly edited sites more closely matching the optimal sequence context than lowly edited ones (Figure 1—figure supplement 1F). Third, principal component analysis of editing proportions at significant sites over the different lines clearly separated the ECT2-FLAG-ADAR fusion lines from the control lines (Figure 1F, Figure 1—figure supplement 1G). Application of subsequent filtering steps, including removal of non-(A-to-G) mismatches and of potential line-specific single-nucleotide variants (see Materials and methods), resulted in a final list of 16,176 edited sites for aerial tissues and 19,242 for roots, corresponding to 4864 and 5052 genes (ECT2 HT-targets), respectively (Figure 1B, Supplementary file 1). In both cases, this represents 27% of all expressed genes. We note that the editing proportions were generally low (Figure 1D) compared to previous work in *Drosophila* (Xu et al., 2018), perhaps in part due to the limited number of cells that express ECT2 (Arribas-Hernández et al., 2018; Arribas-Hernández et al., 2020). Indeed, the *ADAR* expression level (TPMs) correlated strongly with editing proportions among *ECT2-FLAG-ADAR* lines (Figure 1G, Figure 1—figure supplement 1H), and editing proportions were higher for target mRNAs that are coexpressed with *ECT2* in a large percentage of cells according to single-cell RNA-seq (Denyer et al., 2019; Figure 1H), lending further support to the conclusion that the observed editing is ADAR-specific and driven to target mRNAs by ECT2. Hence, HyperTRIBE can be used to identify targets of RNA-binding proteins in planta.

### HyperTRIBE is highly sensitive and identifies primarily m^6^A-containing transcripts as ECT2 targets

**Figure 2.**
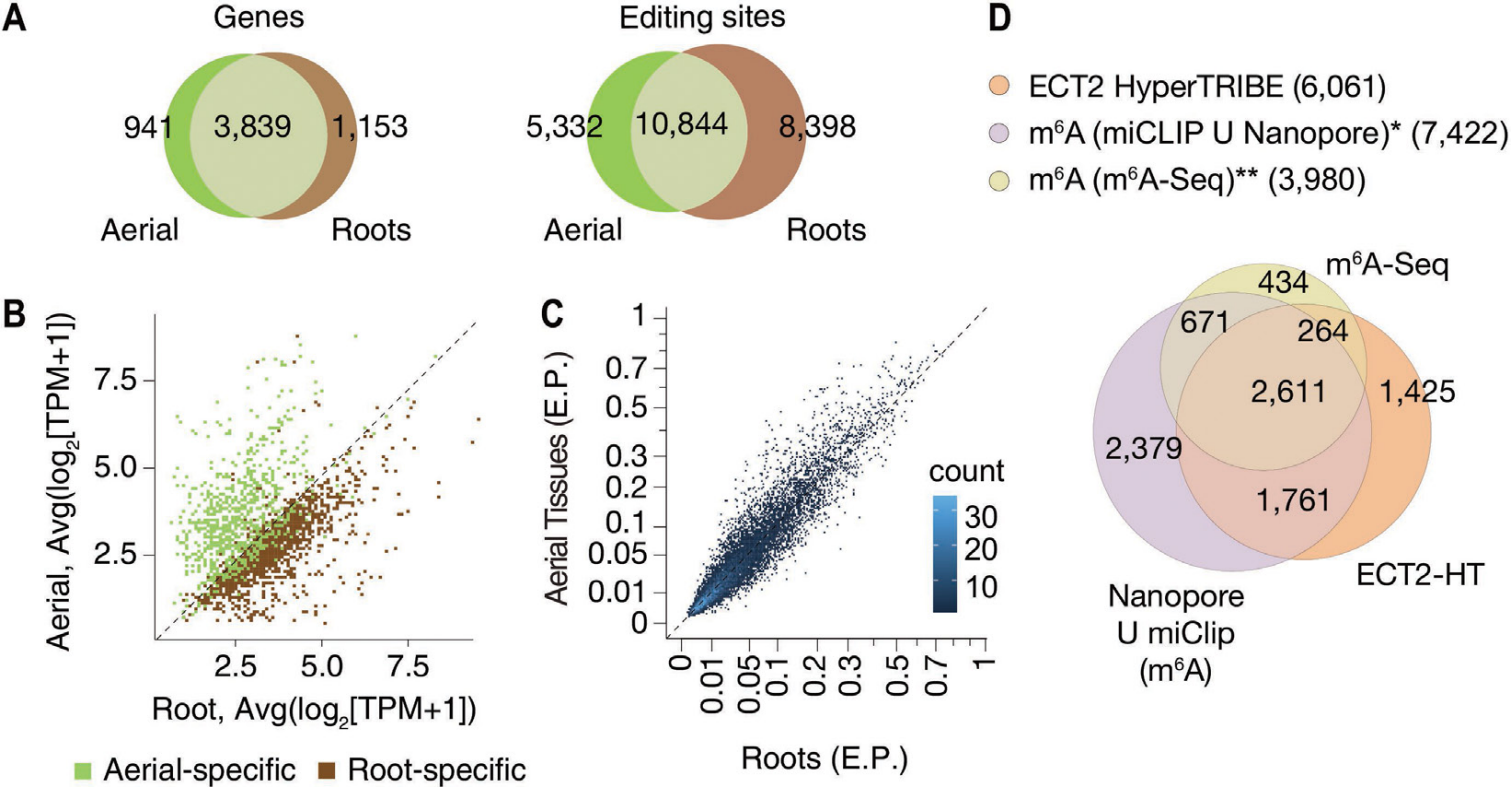
HyperTRIBE identifies m^6^A-reader targets in plants. (**A**) Overlap between ECT2-HT targets (genes and editing sites) in roots and aerial tissues, based on genes commonly expressed in both tissues. (**B**) Scatterplot showing the expression levels in roots and aerial tissues (mean log_2_(TPM+1) over the five ECT2-HT control samples) of the genes identified as aerial or root-specific targets. (**C**) Scatterplot of the editing proportions (E.P.) of significant editing sites in ECT2-HT for aerial vs root tissues. (**D**) Overlap between ECT2-HT targets and m^6^A-containing genes. *Parker et al., 2020; ** Shen et al., 2016.

To evaluate the properties of ECT2 HT-targets, we first noted that most of them were common between root and aerial tissues (Figure 2A), as expected given the recurrent function of ECT2 in stimulating cell division in all organ primordia (Arribas-Hernández et al., 2020). In agreement with this result, most of the targets specific to root or aerial tissues were simply preferentially expressed in either tissue (Figure 2B). Moreover, the significant editing sites in roots and aerial tissues had a considerable overlap (Figure 2A), and their editing proportions were similar in the two tissues (Figure 2C). Of most importance, we observed a large overlap between the ECT2 HT-targets and m^6^A-containing transcripts mapped by different methods in seedlings (Shen et al., 2016; Parker et al., 2020) as more than 76% of ECT2 HT-targets had m^6^A support by either study (Figure 2D). These results validate our HyperTRIBE experimental setup and data analysis, and confirm that ECT2 binds predominantly to m^6^A-containing transcripts in vivo.

### ECT2-mCherry can be specifically UV-crosslinked to target RNA in vivo

**Figure 3.**
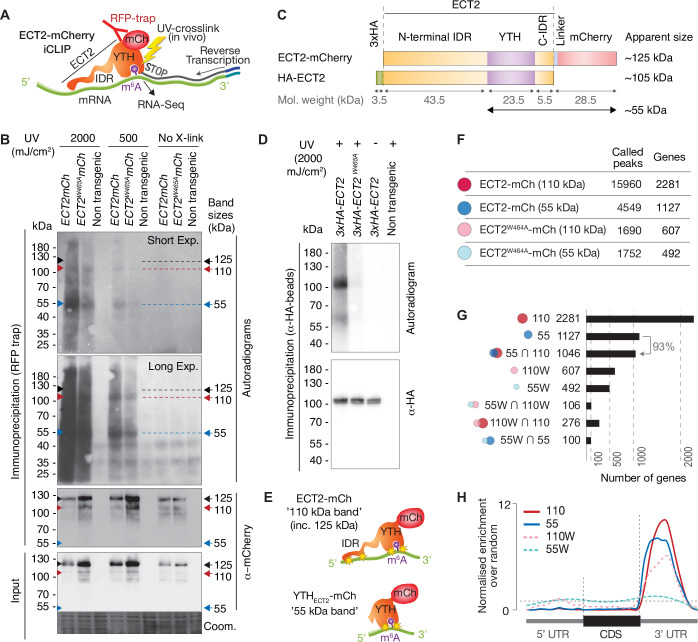
RNA-binding properties of ECT2 revealed by CLIP. (**A**) iCLIP experimental design. (**B**) Upper panels: autoradiogram (top) and α-mCherry protein blot (below) of RFP-trap immuno-purifications. Samples are cell extracts from 12-day-old seedlings expressing *ECT2-mCherry* or *ECT2^W464A^-mCherry* in the *ect2-1* mutant background after in vivo UV-crosslinking as indicated, and subjected to DNase digestion, partial RNase digestion, and 5’-^32^P labeling of RNA. Non-transgenic, Col-0 wild type. Lower panels: α-mCherry protein blot of the same extracts before immunoprecipitation (input) and Coomassie staining of the membrane. Sizes corresponding to full length ECT2-mCherry (~125 kDa) and the most apparent RNA bands are indicated with arrows. A repeat of the experiment with independently grown and crosslinked tissue is shown in the Figure 3—figure supplement 1A. (**C**) Schematic representation of ECT2-mCherry and HA-ECT2 fusion proteins with their apparent size (electrophoretic mobility). The molecular weight of each region is indicated. Notice that IDRs tend to show higher apparent sizes (lower electrophoretic mobility) than globular domains. (**D**) Equivalent to **B** with lines expressing *3xHA-ECT2* variants in the *ect2-1* background, α-HA immuno-purifications and α-HA detection by western blot. (**E**) Cartoon illustrating the nature of the bands of labelled RNA co-purifying with ECT2-mCherry. Yellow stars indicate possible crosslinking sites. (**F**) Number of called peaks and genes detected from the four iCLIP libraries sequenced for this study (Figure 3—figure supplement 3). (**G**) Upset plot showing single and pairwise combinations of genes for the four sequenced iCLIP libraries. Additional intersections can be found in the Figure 3—figure supplement 4. (**H**) Metagene profiles depicting the enrichment along the gene body (5’UTR, CDS or 3’UTR) of the called iCLIP peaks detailed in **F**. Figure 3—source data 1.Uncropped labelled panels and raw image files - Figure 3B, D.

We next moved on to independent target and binding site identification via iCLIP (Figure 3A). We used transgenic lines expressing functional ECT2-mCherry under the control of the endogenous *ECT2* promoter in the *ect2-1* knockout background (Arribas-Hernández et al., 2018; Arribas-Hernández et al., 2020) to co-purify mRNAs crosslinked to ECT2 for iCLIP. Lines expressing the *ECT2^W464A^-mCherry* variant were used as negative controls because this Trp-to-Ala mutation in the hydrophobic methyl-binding pocket of the YTH domain abrogates the increased affinity for m^6^A-RNA (Li et al., 2014b; Xu et al., 2014; Zhu et al., 2014). Accordingly, the point mutant behaves like a null allele in plants despite its wild-type-like expression pattern and level (Arribas-Hernández et al., 2018; Arribas-Hernández et al., 2020).

**Figure 3—figure supplement 1.**
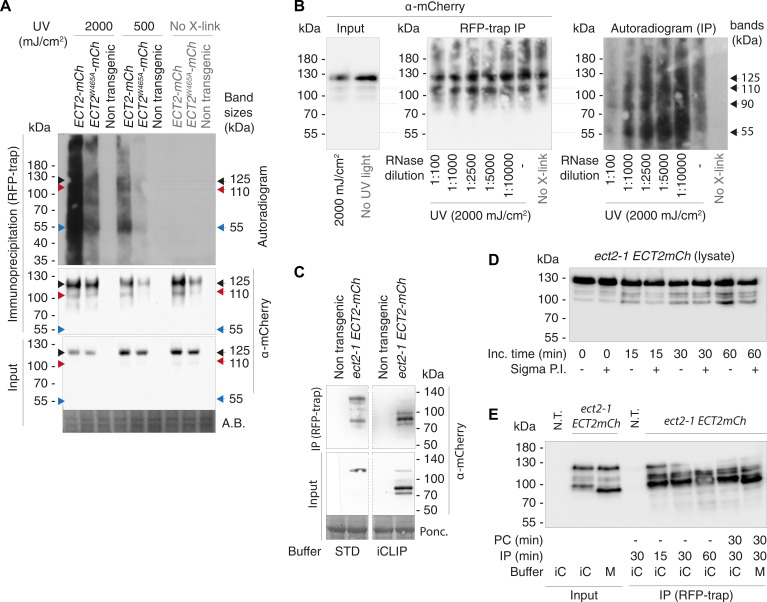
UV-crosslinked RNA co-purifies with ECT2-mCherry in a pattern that depends on the proteolytic cleavage of the ECT2 intrinsically disordered region (IDR) in the lysate. (**A**) Independent repeat of the CLIP experiment in Figure 3B. Sizes corresponding to full-length ECT2-mCherry (~125-kDa) (Figure 3C) and the most apparent RNA bands are indicated. (**B**) Same as (**A**) using a gradient of increasing concentrations of RNase I. Dilutions refer to the 100 U/μL stock, from which 5 μL were added to 100 μL reactions (e.g., 1:5000 corresponds to the final concentration of 1 U/mL used in all other CLIP experiments and for construction of iCLIP libraries). Note that the background in the IP-western blot is presumably due to lack of cooling during the 3-hr-long SDS-PAGE in this first experiment. (**C**) α-mCherry protein blot of lysates (input) or immunopurifications (RFP-trap) incubated for 1 hr at 4°C in either standard (STD) IP buffer (50 mM Tris-HCl, pH 7.5, 150 mM NaCl, 5 mM MgCl_2_, 10% glycerol, 4 mM DTT, 0.1% Nonidet P-40) or iCLIP buffer (50 mM Tris-HCl pH 7.5, 150 mM NaCl, 4 mM MgCl_2_, 5 mM DTT, 1% SDS, 0.25% sodium deoxycholate, 0.25% Igepal), both supplemented only with Roche EDTA-free Protease Inhibitor Cocktail (1 tablet/10 mL). All IP or input lanes have been developed identically on the same membrane. Dashed lines indicate cropping of lanes containing samples irrelevant for this work. Part of the input/non-transgenic/STD buffer sample lane was accidentally left out when the membrane was developed (the thin line indicates the border of the photograph), but the remaining half lane suggests absence of signal. (**D**) α-mCherry protein blot of cell extracts incubated for increasing amounts of time (at 4°C) in iCLIP buffer supplemented with 4 mM PMSF, 1 tablet/10 mL of Complete Protease Inhibitor Cocktail (Roche), and with or without Sigma Protease Inhibitor Optimized for Plant Extracts (1/30 vol). The progressive, protease inhibitor-sensitive appearance of <125kDa ECT2-mCherry species is evidence of their generation by proteolysis in the lysate. (**E**) α-mCherry protein blot of lysates (input) or immunopurifications (RFP-trap) from plants expressing ECT2-mCherry using different preclearing (PC) and immunoprecipitation (IP) times at 4°C, either in iCLIP buffer (iC) or in a milder (M) variant with ½ amount of detergents, both supplemented with 1 mM PMSF and 1 tablet/10 mL of Complete Protease Inhibitor Cocktail (Roche). Again, the progressive appearance of <125kDa ECT2-mCherry species with increasing incubation time is seen. Figure 3—figure supplement 1—source data 1.Uncropped labeled panels and raw image files: Figure 3—figure supplement 1A-E.

**Figure 3—figure supplement 2.**
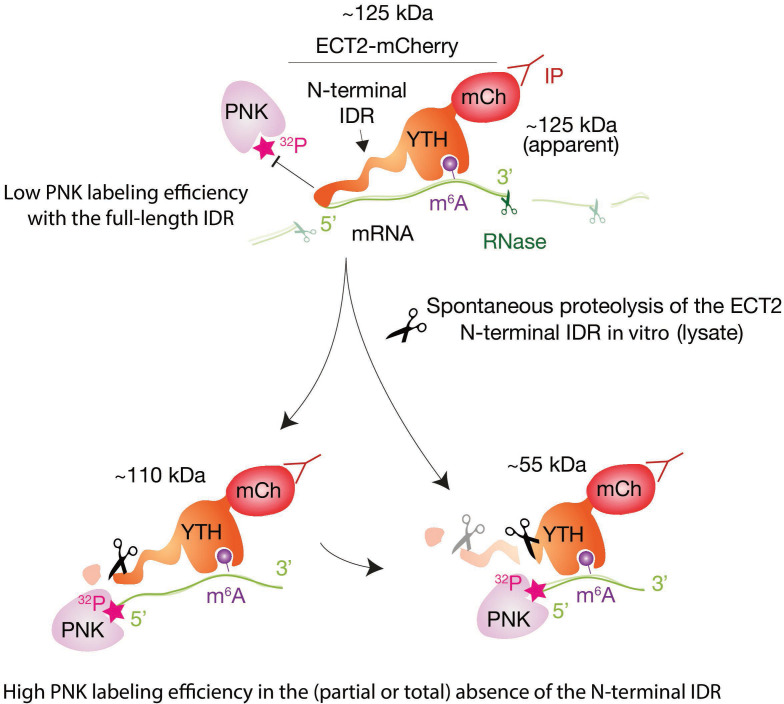
Illustration of RNA-binding properties of ECT2 revealed by CLIP. Interpretation of the pattern of labeled RNA in CLIP experiments due to the spontaneous proteolysis of the ECT2 intrinsically disordered region (IDR) in the lysate, and the different labeling efficiency of bound RNA: RNA co-migrating with the most abundant ECT2-mCherry fragment (full-length, ~125kDa) is barely labeled while the strongest signal appears at ~55kDa (the size of the YTH domain fused to mCherry), where protein abundance is below the western blot detection limit (Figure 3B and C). This observation suggests limited accessibility of 5′-ends of full length ECT2-bound RNA to polynucleotide kinase (PNK), likely due to binding to the IDR. Supporting this idea, the samples containing full-length protein (’110kDa band’) required fewer PCR cycles to obtain similar amounts of library DNA and generated more unique reads than their ‘55kDa band’ counterparts (Figure 3—figure supplements 3 and 4)*,* indicating that most of the RNA co-purifies with full-length ECT2-mCherry, and the stronger intensity of the ‘55kDa band’ (Figure 3B) is indeed due to differences in labeling efficiency.

**Figure 3—figure supplement 3.**
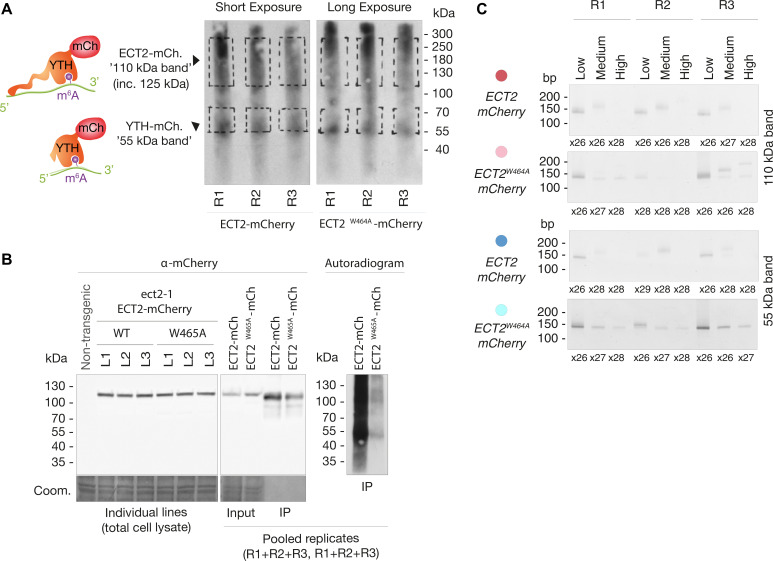
iCLIP Libraries. (**A**) Autoradiograms showing labeled RNA co-purified with ECT2-mCherry and ECT2^W464A^-mCherry in the replicates used to generate iCLIP libraries. Handwritten dashed lines on the films were used as guides to excise the corresponding membrane pieces (‘110 ’Da’ and ‘55kDa’ bands) with a scalpel. The apparently comparable amount of labeled RNA in the two panels, generated from two different membranes and gels run and blotted identically, but separately is a product of different exposure times, which were adjusted in each case for optimal visualization of the RNA. (**B**) Left panel: α-mCherry protein blot from cell extracts of the 3 + 3 independent lines used for iCLIP. Middle and right panels: α-mCherry protein blot and autoradiogram of the cell extracts (input) and the RFP-trap IPs used to prepare the iCLIP libraries. Because samples for ECT2-mCherry and ECT2W464A-mCherry samples were run in separate gels to prevent cross-contamination, pooled aliquots were saved to compare the extent of ECT2 degradation and RNA-binding between them. (**C**) PCR reactions (number of cycles is indicated) used to prepare iCLIP libraries. For each one of the four different libraries, the three corresponding PCRs (low, medium, and high molecular weight) were combined according to their relative concentrations to compensate for inequalities. Notice that the size of the cDNA-insert to be mapped to the genome is expected to be the size of the PCR product minus the length of the P3/P5 Solexa primers and the barcode (128 nt in total) (Huppertz et al., 2014). Therefore, the low molecular weight bands cut at [70–85]-nt on the cDNA gel, with [20–35]-nt-cDNA + 52-nt-primer, generate [145–155]-nt PCR fragments. The low amount of PCR product from the higher molecular weight bands is to be expected, and the non-matching PCR product sizes of the ECT2W464A-mCherry control samples are likely a product of the low amount of RNA co-purified with the m6A-binding-deficient ECT2 mutant. Figure 3—figure supplement 3—source data 1.Uncropped labeled panels and raw image files: Figure 3—figure supplement 3A-C.

**Figure 3—figure supplement 4.**
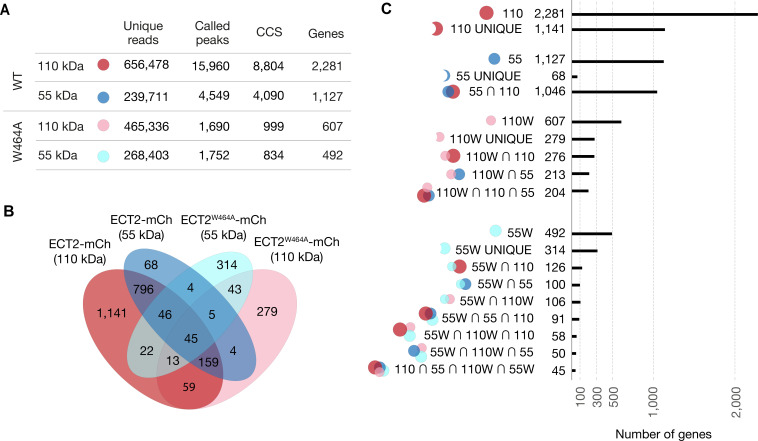
Analysis of ECT2 iCLIP Libraries. (**A**) Number of valid reads (trimmed reads mapped to the *Arabidopsis* genome [TAIR10] discarding PCR duplicates), called peaks ( = crosslink sites) (Krakau et al., 2017), collapsed crosslink sites (CCS) (peaks with the highest PureCLIP-score within clusters and extended by 4 nt in both directions forming 9 nt wide regions), and target genes, obtained from ECT2-mCherry iCLIP libraries. (**B**) Overlaps between gene sets identified for the four ECT2-mCherry iCLIP libraries generated in this study. (**C**) Upset plot displaying gene counts according to different sets defined from the four iCLIP libraries.

To test the feasibility of iCLIP, we first assessed the specificity of RNA co-purified with ECT2-mCherry after UV illumination of whole seedlings by 5′-radiolabeling of the immunoprecipitated RNP complexes followed by SDS-PAGE. These tests showed that substantially more RNA co-purifies with wild-type ECT2 than with ECT2^W464A^ upon UV-crosslinking, and that no RNA is detected without UV irradiation or from irradiated plants of non-transgenic backgrounds (Figure 3B, Figure 3—figure supplement 1A). RNAse and DNAse treatments also established that the co-purified nucleic acid is RNA (Figure 3—figure supplement 1B). Thus, UV crosslinking of intact *Arabidopsis* seedlings followed by immunopurification successfully captures ECT2-RNA complexes that exist in vivo. Curiously, although the pattern of ECT2-RNA complexes with bands migrating at ~110 and 55kDa is highly reproducible, it does not correspond to the majority of the purified ECT2-mCherry protein, which runs at ~125kDa in SDS-PAGE (Figure 3B and C). A variety of control experiments (Figure 3—figure supplement 1C-E), most importantly the disappearance of additional bands with use of an N-terminal rather than a C-terminal tag (Figure 3C and D), indicate that the band pattern arises as a consequence of proteolytic cleavage of the N-terminal IDR in the lysis buffer, such that fragments purified using the C-terminal mCherry tag include the YTH domain with portions of the IDR of variable lengths (Figure 3—figure supplement 2). Comparative analysis of RNA in 55-kDa and 110–125-kDa complexes may, therefore, provide insight into the possible role of the N-terminal IDR of ECT2 in mRNA binding (Figure 3E), an idea consistent with the comparatively low polynucleotide kinase labeling efficiency of full-length ECT2-mCherry-mRNA complexes (~125kDa) (Figure 3B, Figure 3—figure supplement 2). Thus, we prepared separate iCLIP libraries from RNA crosslinked to ECT2-mCherry/ECT2^W464A^-mCherry that migrates at ~110–280kDa (‘110-kDa band’) and at ~55–75kDa (’55-kDa band’) (Figure 3—figure supplement 3) to investigate the possible existence of IDR-dependent crosslink sites, and thereby gain deeper insights into the mode of YTHDF binding to mRNA in vivo.

### ECT2-mCherry iCLIP peaks are enriched in the 3′-UTR of mRNAs

**Figure 4.**
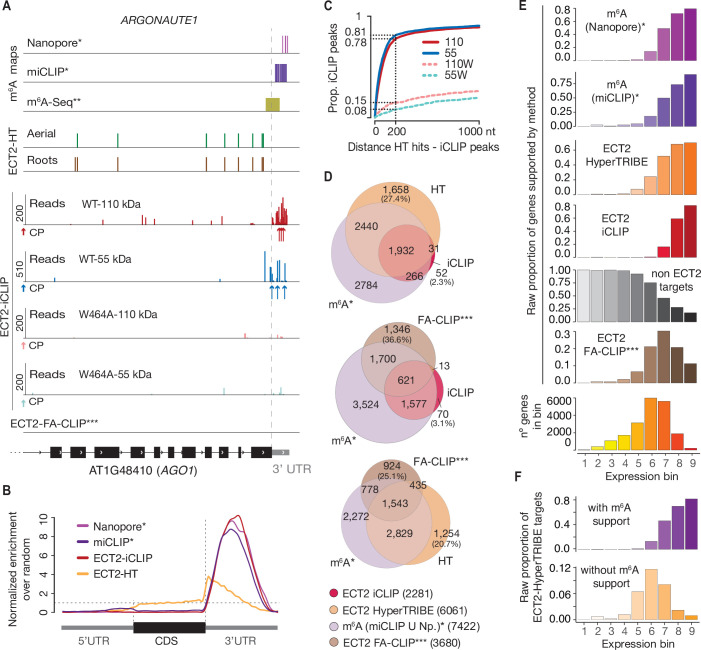
CLIP identifies bona-fide ECT2 targets. (**A**) Example of an ECT2 target (*AGO1*) showing the distribution of m^6^A sites*^,^ **, ECT2-iCLIP reads and peaks, ECT2-HT edited sites, and FA-CLIP peaks*** along the transcript. CP, called peaks. See more examples in the Figure 4—figure supplement 1. (**B**) Metagene profiles comparing the distributions along the gene body of ECT2-mCherry iCLIP peaks (wild type, 110-kDa band), ECT2-HT editing sites (in roots and aerial tissues) and m^6^A sites*. (**C**) Proportion of ECT2 iCLIP peaks within a given distance from the nearest ECT2-HT edited site. Numbers indicated on the y-axis show the proportion of ECT2 iCLIP peaks less than or equal to 200 nt from the nearest ECT2-HT edited site. (**D**) Overlap between genes supported as containing m^6^A or ECT2 targets by the different techniques indicated. The ECT2-HT target set includes the sum of targets identified in root and aerial tissues. Additional overlaps are shown in the Figure 4—figure supplement 2. (**E**) Proportions of genes in each expression bin either containing m^6^A or supported as ECT2 targets by the indicated techniques. (**F**) Proportion of ECT2-HT targets with or without support from m^6^A data (Nanopore*, miCLIP* or m^6^A-Seq**) in each expression bin. * Parker et al., 2020; ** Shen et al., 2016; *** Wei et al., 2018.

We identified a total of 15,960 iCLIP ‘peaks’ or crosslink sites (i.e., single-nucleotide positions called by PureCLIP from mapped iCLIP reads [Krakau et al., 2017]) in 2281 genes from the 110-kDa band of wild-type ECT2-mCherry (henceforth referred to as ECT2 iCLIP peaks and targets, respectively). In the corresponding 55-kDa band, 4549 crosslink sites in 1127 genes were called, 93% of them contained in the 110-kDa target set (Figure 3F and G, Figure 3—figure supplement 4, Supplementary file 2). We note that these numbers perfectly agree with the idea of the 55-kDa band containing only YTH domain crosslink sites, while the full length may also include IDR crosslink sites. Importantly, for both libraries, the majority of crosslink sites mapped to the 3′-UTRs of mRNAs (Figure 3H, see Figure 4A, and Figure 4—figure supplement 1 for more examples), coincident with the main location of m^6^A (Figure 4B; Parker et al., 2020). Accordingly, the 3′-UTR specificity was largely lost in RNA isolated from 55-kDa ECT2^W464A^ (Figure 3H), for which neither YTH domain nor IDR binding to RNA can be expected. Finally, iCLIP targets in full-length (110-kDa band) ECT2 WT and ECT2^W464A^ overlapped only marginally (Figure 3G), providing molecular proof of the dependence of m^6^A-binding activity for ECT2 function. Nonetheless, the bias towards occurrence in the 3′-UTR was only reduced, not abolished, for crosslinks to the full-length ECT2^W464A^ protein, providing another indication that the IDR itself is able to associate with RNA-elements in 3′-UTRs (Figure 3H). We elaborate further on this important point by analysis of IDR-specific crosslinks to wild-type ECT2 after in-depth validation of sets of ECT2 target mRNAs and determination of the sequence motifs enriched around m^6^A and ECT2 crosslink sites.

**Figure 4—figure supplement 1.**
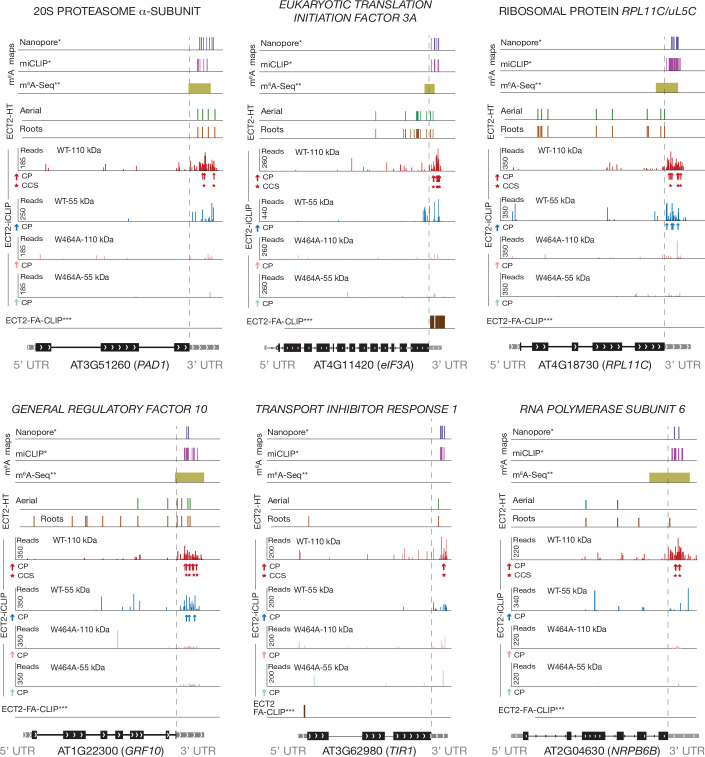
Distribution of m^6^A and ECT2 sites on ECT2 targets. Representative examples of ECT2 targets showing the distribution of m^6^A sites^*, **^, ECT2-iCLIP reads and peaks, ECT2-HT edited sites, and FA-CLIP peaks^***^ along the transcript. CP, called peaks ( = crosslink sites); CCS, collapsed crosslink sites. * Parker et al., 2020; ** Shen et al., 2016; *** Wei et al., 2018.

**Figure 4—figure supplement 2.**
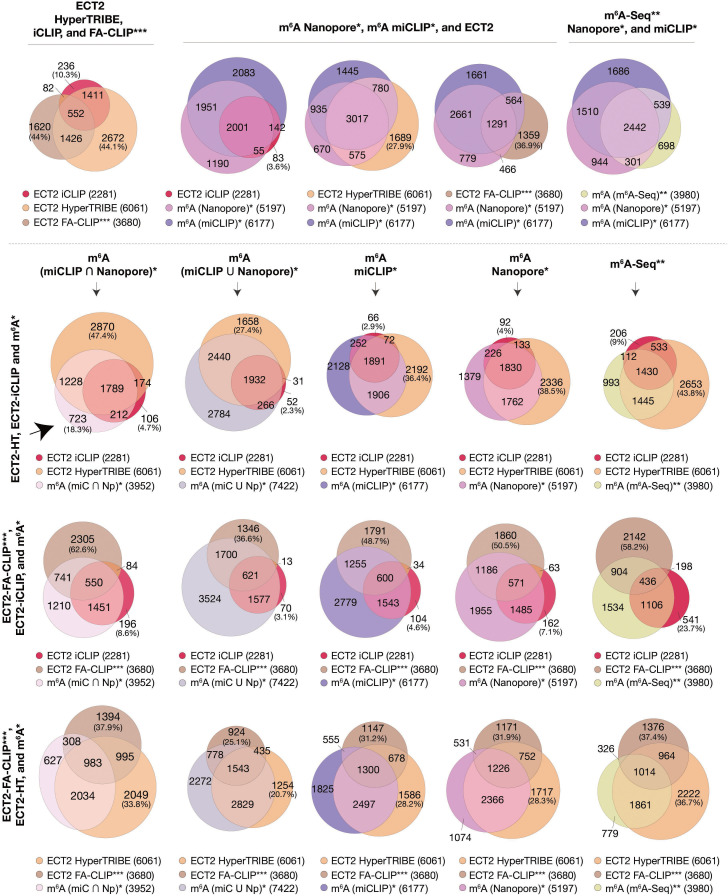
Overlaps between m^6^A-containing genes and ECT2 targets datasets. Overlap between genes supported as containing m^6^A or ECT2 targets by the different techniques indicated. The ECT2-HT target set includes the sum of targets identified in root and aerial tissues. * Parker et al., 2020; ** Shen et al., 2016; *** Wei et al., 2018.

**Figure 4—figure supplement 3.**
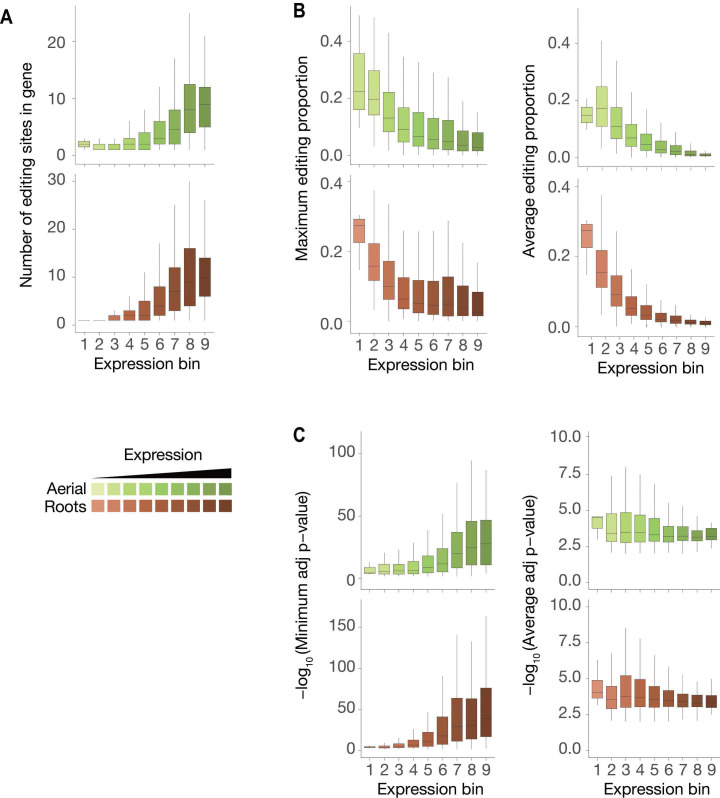
Characteristics of ECT2-HyperTRIBE editing sites relative to target expression levels. (**A–C**) Number of significant editing sites (**A**), maximum or average editing proportions (**B**), and significance of editing sites according to either minimum or average -log_10_(adjusted p-value) per gene (**C**) in ECT2-HT targets split according to their expression levels (calculated as in Figure 2B), in both aerial and root tissues. The number of detected editing sites increases with the expression level of the targets (**A**) while, unexpectedly, the editing proportion decreases (**B**). This may be caused by dilution as the ECT2 promoter is active only in highly dividing cells (Arribas-Hernández et al., 2020) while some abundant target mRNAs may be ubiquitously expressed. Nevertheless, the average statistical significance over all edited sites per target mRNA did not change with the expression level (**C**, right panels), indicating that our HyperTRIBE experimental setup and data analysis identifies targets across a wide range of expression levels including lowly expressed transcripts. This suggests that the significance of differential editing versus a negative control, rather than raw editing proportion or number of editing sites, is the preferred parameter for definition and ranking of targets.

### iCLIP sites tend to be in the vicinity of HyperTRIBE editing sites

To evaluate the congruence of the results obtained by iCLIP and HyperTRIBE, we investigated the cumulative number of iCLIP sites as a function of distance to the nearest editing site determined by HyperTRIBE. This analysis showed a clear tendency for iCLIP peaks called with ECT2^WT^-mCherry, but not for ECT2^W464A^-mCherry, to be in the vicinity of editing sites (Figure 4C), indicating that the majority of called iCLIP peaks identify genuine ECT2-binding sites on mRNAs. Similar tendencies of proximity between iCLIP peaks and HyperTRIBE editing sites were previously observed for a *Drosophila* hnRNP protein (Xu et al., 2018). Although manual inspection of individual target genes confirmed these tendencies, it also revealed that ADAR-edited sites are too dispersed around iCLIP peaks to give precise information on the actual ECT2-binding sites (Figure 4A, Figure 4—figure supplement 1). Therefore, we used both HyperTRIBE and iCLIP for gene target identification, but relied on iCLIP peaks for motif analyses.

### ECT2 targets identified by iCLIP and HyperTRIBE overlap m^6^A-containing transcripts

To examine the quality of our target identification in further detail, we analyzed the overlap between ECT2 targets identified by iCLIP and HyperTRIBE. This analysis also included m^6^A mapping data obtained with either m^6^A-seq (Shen et al., 2016) or the single-nucleotide resolution methods miCLIP and Nanopore sequencing (Parker et al., 2020) as young seedlings were used in all cases. ECT2 targets identified by iCLIP and HyperTRIBE showed clear overlaps, both with each other and with m^6^A-containing transcripts, further supporting the robustness of ECT2 target identification via combined iCLIP and HyperTRIBE approaches (Figure 4D, upper panel, Figure 4—figure supplement 2). Importantly, although some m^6^A targets are expected not to be bound by ECT2 because of the presence of MTA in cells that do not express ECT2 (Arribas-Hernández et al., 2020), only 18% of the high-confident set of m^6^A-containing genes (with support from miCLIP and Nanopore) did not overlap with either ECT2 iCLIP or HT target sets (Figure 4—figure supplement 2*,* arrow). We also observed that HyperTRIBE identifies approximately three times more ECT2 targets than iCLIP, possibly because of the bias towards high abundance inherent to purification-based methods like iCLIP (Wheeler et al., 2018). To test this idea, we compared the distribution of target mRNAs identified by the different techniques across nine expression bins. As expected, a bias towards highly abundant transcripts was evident for iCLIP-identified targets compared to HyperTRIBE (Figure 4E). We also observed a similar bias for m^6^A-containing transcripts detected by miCLIP, another purification-based method, and in the Nanopore dataset (Figure 4E), probably explained by its relatively low sequencing depth (Parker et al., 2020). These observations also suggest that the higher sensitivity of HyperTRIBE (analyzed in detail in Figure 4—figure supplement 3) explains the lack of m^6^A support (by Nanopore or miCLIP) for 28% of ECT2 HT-targets (1689) compared to only 4% (83) of ECT2 iCLIP targets (Figure 4D*,* Figure 4—figure supplement 2, upper row) since HT-targets may simply include genes that escape detection by m^6^A mapping methods due to low expression. Indeed, ECT2-HT targets without any m^6^A support were distributed in lower-expression bins compared to those with m^6^A support (Figure 4F). Intriguingly, ECT2 FA-CLIP targets (Wei et al., 2018) did not show a bias towards highly expressed genes as their distribution over expression bins largely reflected that of the total number of genes (Figure 4E), and as many as 37% of FA-CLIP targets did not have m^6^A support (Figure 4D*,* Figure 4—figure supplement 2, upper row). In summary, these analyses show that ECT2 iCLIP and HT target sets are in excellent agreement with each other and with independently generated m^6^A maps, and that HyperTRIBE identifies targets below the detection limit of other techniques.

### ECT2 crosslink sites coincide with m^6^A miCLIP sites and are immediately upstream of Nanopore m^6^A sites

**Figure 5.**
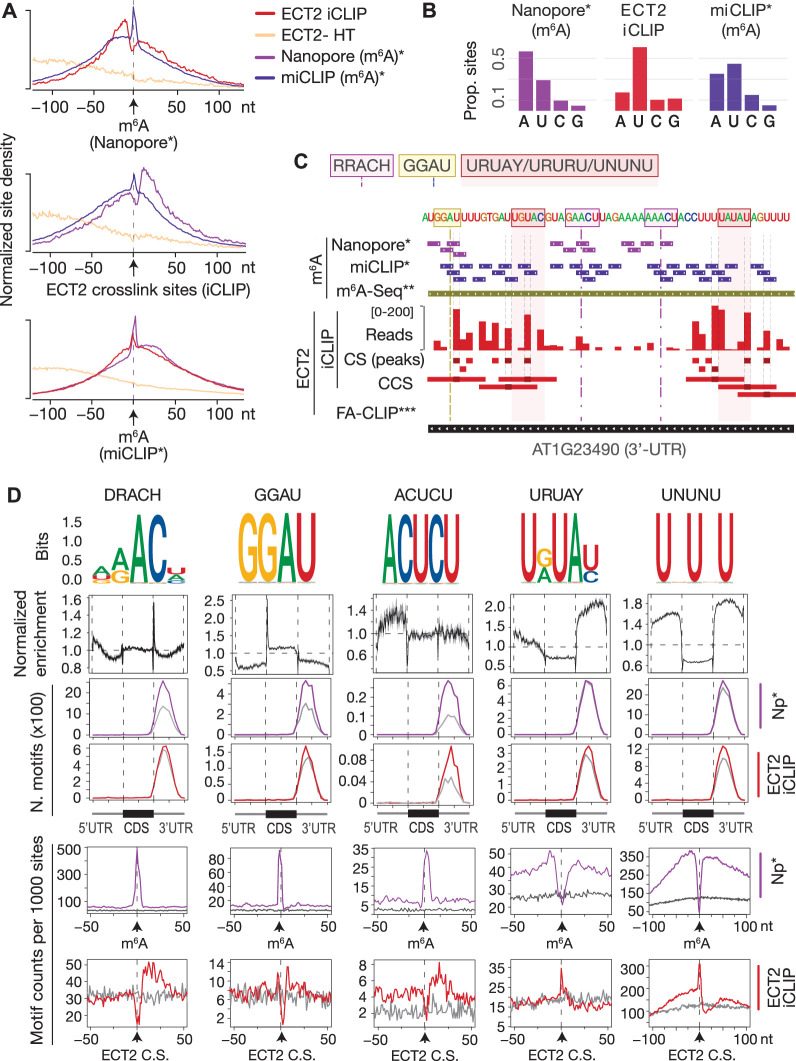
ECT2 UV-crosslinks to uridines in the immediate vicinity of DR(m^6^A)CH or GG(m^6^A)U sites. (**A**) Normalized density of sites at and up to +/-100 nt of either m^6^A-Nanopore*, m^6^A-miCLIP* or ECT2-iCLIP sites. (**B**) Proportion of m^6^A and ECT2-iCLIP sites at each nucleotide by the different methods. (**C**) View from IGV browser illustrating the presence of RRACH, GGAU and U-rich motifs in the vicinity of m^6^A and ECT2 sites in the 3’-UTR of AT1G23490 (*ARF1*). CS, crosslink sites; CSS, collapsed crosslink sites. (**D**) Key motifs analyzed in this study. From top to bottom: (1) motif logos for derived position weight matrices (PWMs); (2) normalized enrichment of motif locations across gene body; (3-4) total number of the relevant motif found at m^6^A-Nanopore* (3) or ECT2-iCLIP (4) sites according to gene body location. Gray lines indicate numbers found in a gene-body location-matched background set of sites of equivalent number; (5-6) distribution of the relevant motif relative to m^6^A-Nanopore* (5) or ECT2–iCLIP (6) sites. Gray lines represent the distribution for the same gene-body location-matched set as derived in the panels above. * Parker et al., 2020; ** Shen et al., 2016; *** Wei et al., 2018.

To characterize the sequence composition and exact positions of ECT2-binding sites relative to m^6^A, we first used the high resolution of iCLIP data to examine the position of ECT2 crosslink sites relative to m^6^A sites, determined at single-nucleotide resolution (Parker et al., 2020). This analysis showed that ECT2 crosslinks in the immediate vicinity, but preferentially upstream (~11 nt) of Nanopore-determined m^6^A sites, with a mild depletion at the exact m^6^A site (Figure 5A, upper panel). Furthermore, while m^6^A-miCLIP sites corresponded to m^6^A-Nanopore sites overall, a subset of m^6^A-miCLIP sites were located upstream of m^6^A-Nanopore sites and coincided well with ECT2-iCLIP peaks (Figure 5A). This pattern is probably explained by the fact that the UV illumination used in both iCLIP and miCLIP preferentially generates RNA-protein crosslinks involving uridine (Hafner et al., 2021), also detectable in the datasets analyzed here (Figure 5B and C). Thus, the depletion of ECT2-iCLIP sites at Nanopore-, but enrichment at miCLIP-determined m^6^A sites (Figure 5A), might be explained by the absence of uridine within the RRAC core of the m^6^A consensus motif, and perhaps also to some extent by reduced photoreactivity of the m^6^A base stacking with indole side chains of the YTH domain. Furthermore, the fact that nucleotides at −2, +1, and +2 positions are only expected to contribute sugar-phosphate backbone interactions with the YTH domain (Luo and Tong, 2014b; Theler et al., 2014; Xu et al., 2014) may also contribute to the absence of direct crosslinks at the m^6^A site relative to the adjacent bases.

**Figure 5—figure supplement 1.**
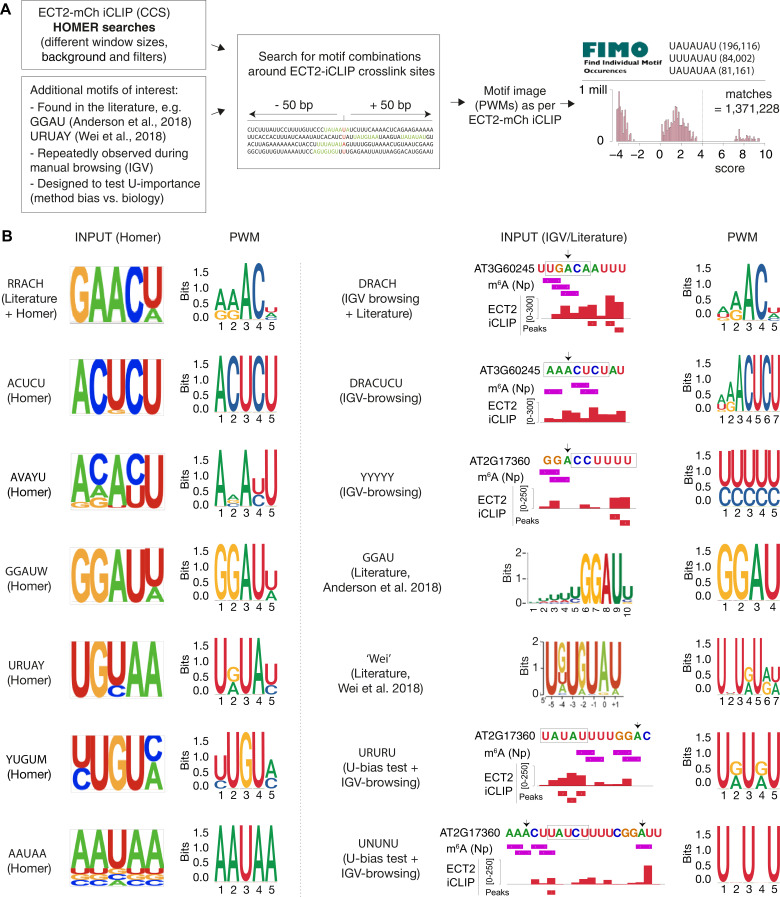
Sources of motifs and generation of position weight matrices (PWMs). (**A**) Relative nucleotide frequencies for motifs of interest with potential sequence redundancies were estimated from windows around ECT2-iCLIP collapsed crosslink sites (CCS) and converted into PWMs that were subsequently used in FIMO to scan for motif matches genome wide. Plot illustrates score distribution for the motif derived from Wei et al., 2018, and vertical dotted line indicates chosen score cutoff. (**B**) Subset of motifs according to source. Left: examples of motifs inspired by Homer searches (left logo) and subsequent PWM used in the analysis (right logo). Right: examples of further motifs derived from other sources. Logo from the relevant paper is shown if the motif derives from literature, and representative examples of IGV-browser screenshots are shown otherwise (motifs are outlined; arrows mark As within DRACH/GGAU contexts; Np, Nanopore [Parker et al., 2020]). Subsequent PWMs are also indicated (right logos).

**Figure 5—figure supplement 2.**
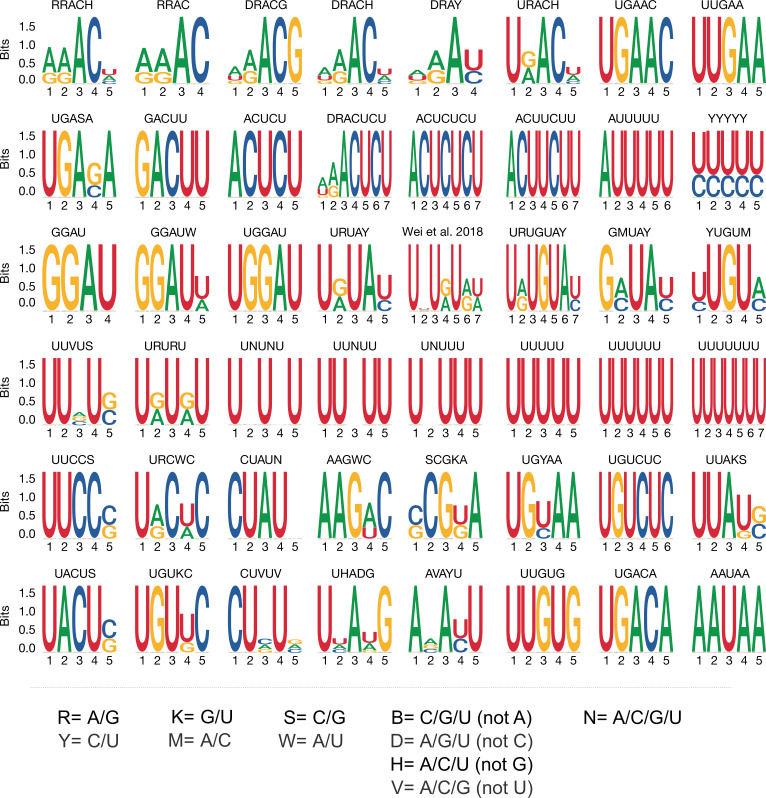
Motif logos generated from position weight matrices. Motif logos represent all of the 48 derived motif position weight matrices considered in the current analysis (see Materials and methods and Figure 5—figure supplement 1 for selection details). UPAC-IUB codes to define multiple nucleotide possibilities in one position are detailed below.

**Figure 5—figure supplement 3.**
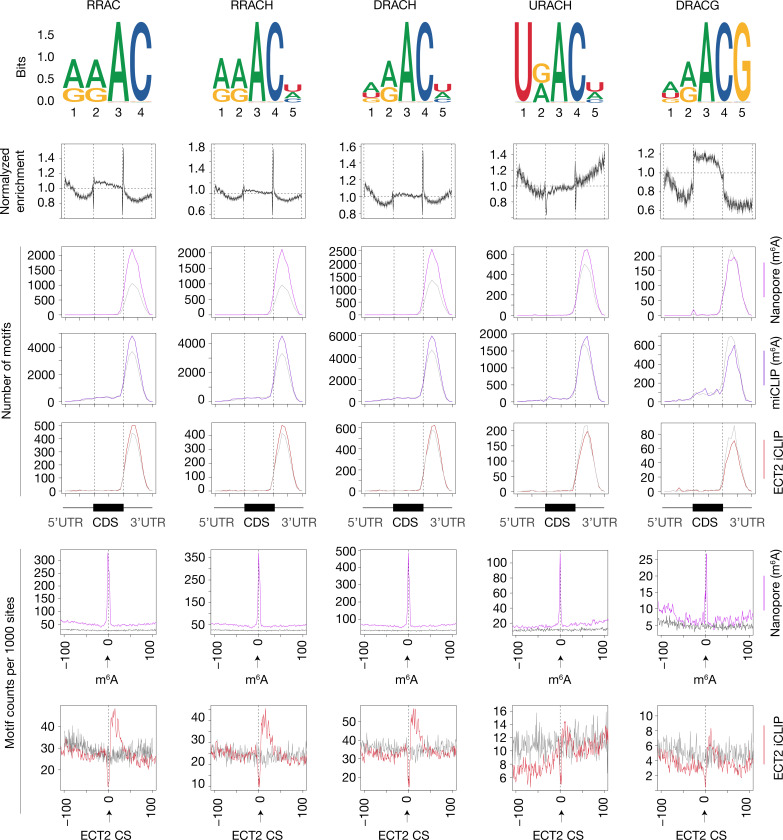
Enrichment of RRACH variants around m^6^A and ECT2 sites. From top to bottom: (1) motif logos for derived position weight matrices (PWMs); (2) normalized enrichment of motif locations across gene body; (3–5) total number of the relevant motif found at m^6^A-Nanopore* (3), m^6^A-miCLIP*, (4) or ECT2-iCLIP (5) sites according to gene body location. Gray lines indicate numbers found in a gene body location-matched background set of sites of equivalent number; (6 and 7) distribution of the relevant motif relative to m^6^A-Nanopore* (6) or ECT2–iCLIP (7) sites. Gray lines represent the distribution for the same gene body location-matched set as derived in the panels above. RRACH shows a slightly higher enrichment over RRAC around m^6^A-Nanopore* sites, which is further increased in the more lenient version DRACH. Accordingly, there is also clear enrichment of URAC. On the contrary, there is no global enrichment of DRACG in m^6^A-Nanopore* datasets along the gene body, and only very modest around m^6^A-Nanopore* sites, highlighting the importance of the final H in DRACH. R = A/G, H = A/C/U, D = A/G/U. * Parker et al., 2020.

**Figure 5—figure supplement 4.**
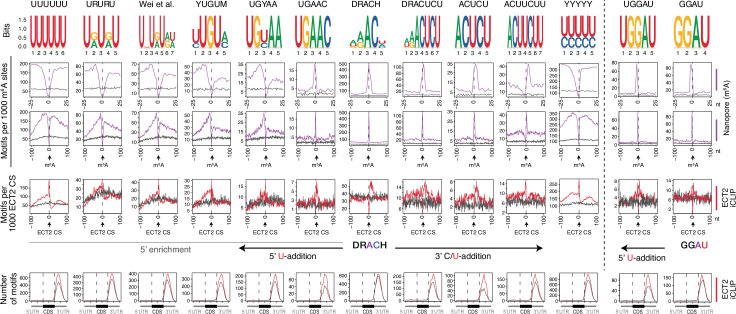
Uridines flanking DRACH result in additional motifs enriched at ECT2 iCLIP sites. Enrichment of DRACH-like motifs containing additive amounts of U(/Y)s at the flanks until there is only U/Ys. Through the upstream U-additions, transition forms like URURU/UGUAY/YUGUM are included. GGAU with addition of one U upstream is also shown on the right side of the figure. From top panel to bottom: (1) motif logos for derived position weight matrices (PWMs); (2-4) distribution of the relevant motif relative to m^6^A-Nanopore (Parker et al., 2020) (2, 3) or ECT2–iCLIP crosslink sites (CS) (4). Gray lines represent the distribution for gene body location-matched background set of sites of equivalent number; (5) total number of the relevant motif found at ECT2-iCLIP sites according to gene body location. Gray lines indicate numbers found in the same gene body location-matched background set. Figure 5—figure supplement 4—source data 1.High quality image file.

### DRACH, GGAU, and U/Y-rich motifs are the most enriched around m^6^A/ECT2 sites

The 5′ shift observed for iCLIP and miCLIP sites relative to Nanopore sites might be explained by a higher occurrence of uridines upstream of m^6^A sites, a particularly interesting possibility given the numerous reports of U-rich motifs enriched around m^6^A sites in plants (Li et al., 2014a; Anderson et al., 2018; Miao et al., 2020; Zhang et al., 2019; Zhou et al., 2019; Luo et al., 2020) and animals (Patil et al., 2016). To investigate the sequence composition around m^6^A and ECT2 sites, we first performed exhaustive unbiased de novo motif searches using Homer (Heinz et al., 2010; Figure 5—figure supplement 1) and extracted all candidate motifs, including the m^6^A consensus motif RRACH, as well as GGAU (Anderson et al., 2018), URUAY (Wei et al., 2018), and several other U-rich sequences. Combined with manually derived candidate motifs (Figure 5—figure supplement 1B), we then calculated position weight matrices (PWMs) for a final set of 48 motifs and scanned for their occurrences genome-wide using FIMO (Grant et al., 2011; Figure 5—figure supplements 1 and 2). This allowed us to determine three key properties. First, the global enrichment of the motifs at locations across the gene body. Second, the total count of occurrences of each motif at m^6^A sites and ECT2-iCLIP crosslink sites compared to a set of sites in non-target mRNAs matching the location within gene bodies of m^6^A/ECT2-iCLIP sites (expected background). Third, the distribution of the motifs relative to m^6^A and ECT2 iCLIP sites. The results of this systematic analysis (Supplementary file 3) were used to select those motifs with a more prominent enrichment at or around m^6^A and ECT2 sites (Figure 5D). This approach defined two major categories of motifs of outstanding interest, RRACH-like and GGAU on the one side, and a variety of U/Y-rich motifs on the other. Figure 5D shows a minimal selection of such motifs, while a more comprehensive compilation is displayed in Figure 5—figure supplements 3 and 4. Not surprisingly, RRACH-like motifs were the most highly enriched at m^6^A sites and showed a clear enrichment immediately downstream of ECT2 crosslink sites in our analyses, with the degenerate variant DRACH being the most frequently observed (Figure 5D, Figure 5—figure supplement 3). Motifs containing GGAU behaved similarly to DRACH, with a sharp enrichment exactly at m^6^A sites and mild enrichment downstream of ECT2 peaks (Figure 5D), supporting a previous suggestion of GGAU as an alternative methylation site (Anderson et al., 2018). The possible roles of the U/Y-rich motifs in m^6^A deposition and ECT2 binding are analyzed in the following sections.

### Neighboring U/Ys result in enriched RRACH- and GGAU-derived motifs

We first noticed that several motifs retrieved around ECT2 crosslink sites by Homer constituted extended versions of **DRACH/GGAU** with *U*s upstream (e.g., *U***GAAC**/*U***GGAU**) or remnants of DR**ACH** with *U/C*s (*Y*s) downstream (e.g., **ACU***CU*). To test whether these motifs are indeed located adjacent to m^6^A, we examined their distribution and enrichment around ECT2 and m^6^A sites. The distributions showed a clear enrichment at m^6^A positions with a shift in the direction of the *U/Y*-extension (see Figure 5D for ACUCU and Figure 5—figure supplement 4 for others). An enrichment over location-matched background sites close to ECT2-iCLIP sites was also apparent (see Figure 5D for ACUCU and Figure 5—figure supplement 4 for others), further supporting that ECT2 preferentially crosslinks to uridines located in the immediate vicinity of DRACH (/GGAU). Thus, several enriched motifs around ECT2 crosslink sites are DRACH/GGAU-derived, and their detection in unbiased searches simply reflects a tendency of methylated DRACH/GGAU sites to be flanked by U/Ys.

### Nature of U/Y-rich motifs more distant from m^6^A sites

U/R-rich motifs without traces of adjacent DRACH (e.g., YUGUM, URUAY, URURU) showed a characteristic enrichment around, but depletion at, m^6^A sites. For some motifs, the enrichment was more pronounced 5′ than 3′ to m^6^A sites (see Figure 5D for URUAY and Figure 5—figure supplement 4 for others). The distance between the site of maximal motif occurrence and the m^6^A site roughly coincided with the shift observed in ECT2 crosslink sites relative to m^6^A (Figure 5A*,* upper panel). Accordingly, these motifs were enriched exactly at ECT2 crosslink sites (see Figure 5D for URUAY and Figure 5—figure supplement 4 for others), suggesting that they may constitute additional m^6^A-independent sites of interaction with ECT2. We also observed that the 3′ enrichment of YYYYY was asymmetric and closer to m^6^A than that of UUUUU/URURU/URUAY (Figure 5—figure supplement 4, second row from the top), indicating a preference for hetero-oligopyrimidine tracts immediately downstream the m^6^A site, as suggested by the 3′-enrichment of DRACUCU-type motifs as described above.

Taken together, these results suggest that *N6*-adenosine methylation preferentially occurs in DRACH/GGAU sequences surrounded by stretches of pyrimidines, with a preference for YYYYY (e.g., CUCU) immediately downstream, URURU (including URUAY) immediately upstream, and UUUUU/UNUNU slightly further away in both directions. The enrichment of ECT2 crosslink sites at these motifs, and the fact that the m^6^A-binding-deficient mutant of ECT2 (W464A) crosslinks preferentially to 3′-UTRs through its N-terminal IDR, indicates IDR-mediated binding to U/R- and Y-rich motifs around m^6^A.

### DRACH/GGAU motifs are determinants of m^6^A deposition at the site, while flanking U(/Y)-rich motifs are indicative of m^6^A presence and ECT2 binding

**Figure 6.**
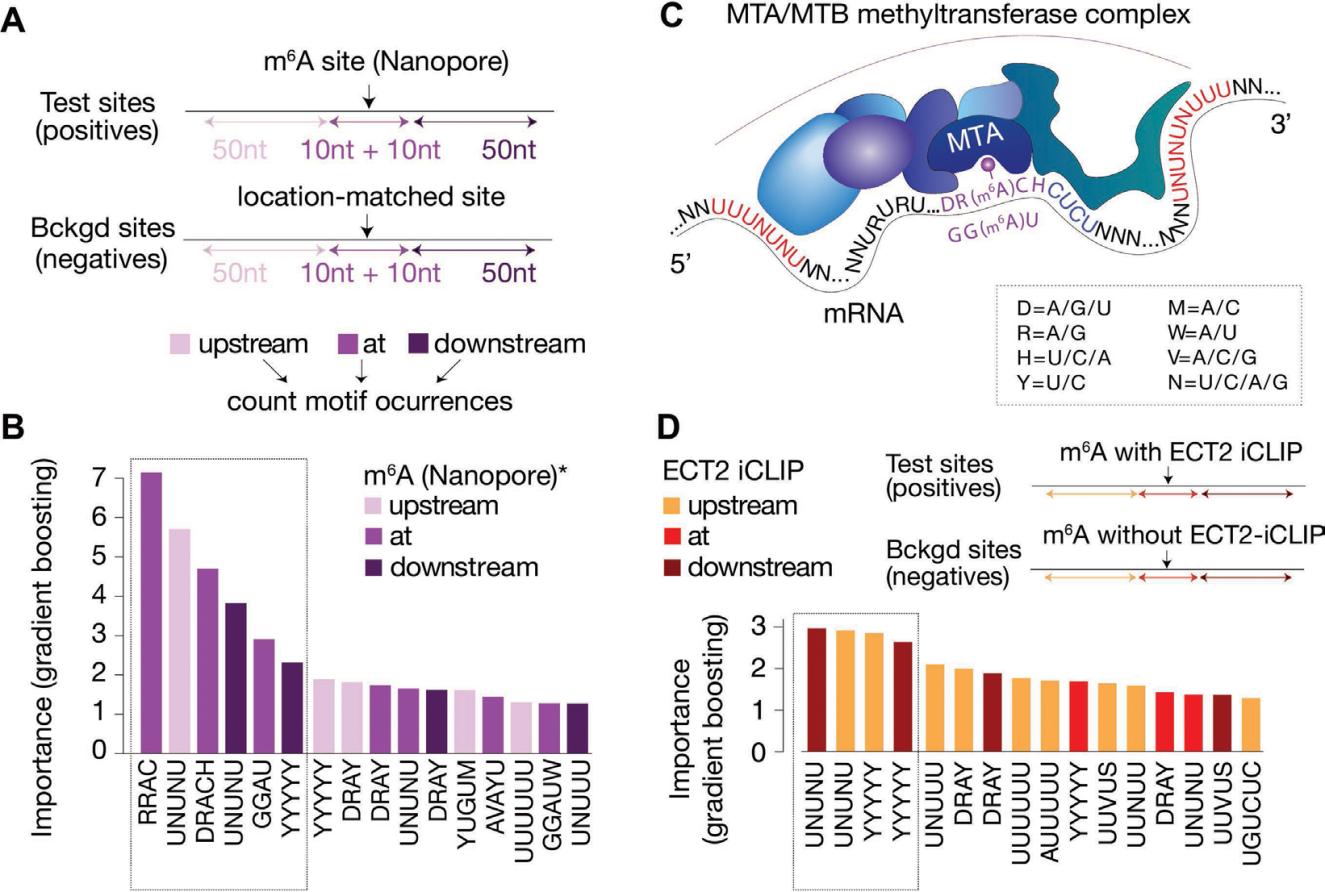
Distal U-rich motifs and at-the-site DRACH/GGAU are determinants for m^6^A deposition. (**A**) Diagram representing the strategy for machine learning model trained to distinguish m^6^A-Nanopore* sites from their respective gene-body location matched background sets. (**B**) Bar plots showing top 16 motif feature importance scores from the m^6^A model, ordered from left to right by importance. The dotted rectangle highlights motifs with outstanding importance compared to the rest. (**C**) Cartoon representing the most important motifs found at and around m^6^A sites. UPAC-IUB codes to define multiple nucleotide possibilites in one position are indicated. (**D**) Machine learning model trained to distinguish between m^6^A sites with and without ECT2 crosslink sites, and the resulting bar plot showing top 16 motif feature importance scores. Nucleotide distances for intervals, order and dotted box are as in **A**/**B**. * Parker et al., 2020.

Since our analysis thus far uncovered several motifs of potential importance for m^6^A deposition and ECT2 binding, we employed machine learning to distinguish m^6^A and ECT2 iCLIP sites from random location-matched background sites using motif-based features. Importantly, the underlying classification model includes all motif features within the same model, allowing an evaluation of the importance of the motifs relative to each other. We used as features the number of matches to each of the 48 motifs (Figure 5—figure supplement 2) in three distinct regions relative to the methylated site according to Nanopore sequencing (Parker et al., 2020), defined as position 0: ‘at’ [–10 nt; +10 nt], ‘down’ [–50 nt; –10 nt], or ‘up’ [+ 10 nt; +50 nt] (Figure 6A). The model involving all motifs could successfully distinguish the methylated sites from the background as indicated by an area under the receiver operating characteristic (ROC) curve (true positive rate versus false positive rate, area under the curve [AUC]) of 0.93, and even a reduced model incorporating only the top 10 features from the full model classified sites largely correctly (AUC = 0.86; Figure 6—figure supplement 1). The top 16 features ordered by importance from the full model confirmed that RRAC/DRACH or GGAU at the site was indicative of the presence of m^6^A (Figure 6B). Interestingly, U/Y-rich sequences (UNUNU and YYYYY in particular) flanking the site were also strongly indicative (Figure 6B). Some motifs showed a skew in their feature importance score, with UNUNU and YUGUM showing a preference to be upstream, and YYYYY downstream (Figure 6B), thus corroborating our previous observations (Figure 6C).

**Figure 6—figure supplement 1.**
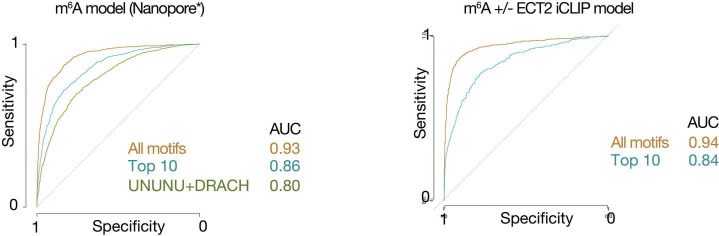
Model performance receiver operating characteristic (ROC) curves for distinguishing sequence preferences of either m^6^A or ECT2-bound sites. ROC curves showing model performance of random forest (gradient boosting) trained models, for distinguishing either m^6^A-Nanopore* sites from their respective random location-matched background sites (left), or m^6^A-Nanopore* sites with and without ECT2-iCLIP crosslink sites (right), with overall performance represented by area under the curve (AUC) values. ‘All motifs’ refers to the full set of 48 motifs (at, upstream, or downstream), ‘Top 10’ to the top 10 most important features from the full model, and ‘UNUNU + DRACH’ includes features derived only from UNUNU or DRACH motifs. *Parker et al., 2020.

We used a similar modeling approach to identify non-m^6^A determinants of ECT2 binding, in this case comparing m^6^A sites within 10 nt distance of ECT2-iCLIP sites to m^6^A sites without ECT2-iCLIP sites nearby (AUC = 0.94, and AUC = 0.84 using only the top 10 features, Figure 6—figure supplement 1). In agreement with previous observations, this model showed flanking U/Y-rich sequences as the main determinants for ECT2 crosslinking (Figure 6D).

### The U(-R) paradox: URURU-like sequences around m^6^A sites repel ECT2 binding, while U-rich sequences upstream enhance its crosslinking

**Figure 7.**
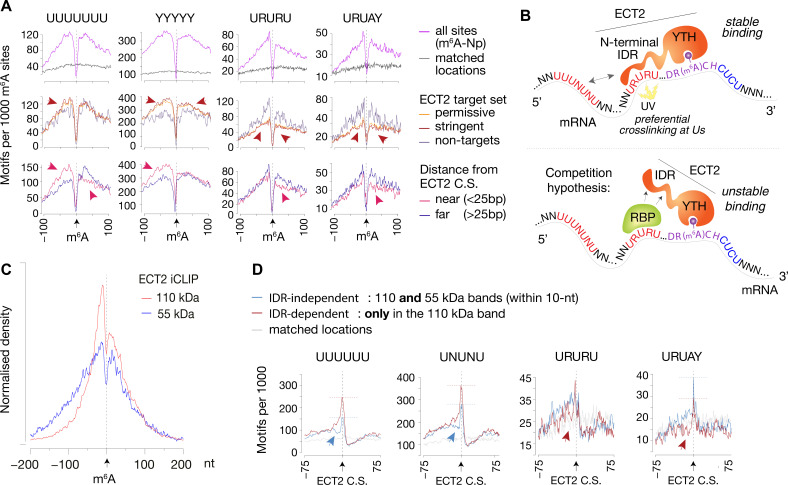
IDR-dependent binding of ECT2 to U-rich motifs 5’ of m^6^A. (**A**) Top panels: Distance-based enrichment of motifs at and around m^6^A-Nanopore (Np, Parker et al., 2020) sites, plotted as motif counts per 1000 m^6^A sites (purple lines). Gray lines indicate the enrichment in a location-matched background set as in Figure 5D. Middle and bottom panels: sites are split according to whether they sit on ECT2 targets (middle), or to distance from the nearest ECT2 crosslink site (for ECT2-iCLIP targets only) (bottom). Additional motifs are shown in the Figure 7—figure supplement 1. (**B**) Cartoon illustrating the ECT2 IDR RNA-binding and competition hypotheses. (**C**) Normalized density of ECT2 iCLIP crosslink sites identified in the libraries corresponding to the 110- and 55-kDa bands (Figure 3B) at and up to +/-200 nt of m^6^A-Nanopore sites. (**D**) Motifs per 1000 ECT2-iCLIP crosslink sites (CS) split according to whether they are found in libraries from both 110-kDa and 55-kDa bands (IDR-independent’), or exclusively (distance > 10 nt) in the 110-kDa band (’IDR-dependent’). Gray lines indicate the enrichment in a location-matched background set as in Figure 5D. Additional motifs are shown in Figure 7—figure supplement 2 and Supplementary file 3.

To investigate the idea of URURU-like motifs as additional sites of ECT2 binding upstream of the m^6^A-YTH interaction site, we split Nanopore-m^6^A sites according to two criteria: (1) whether they occur in ECT2-target transcripts (both permissive and stringent sets analyzed separately), and (2) for ECT2 targets, whether there is an ECT2 crosslink site within 25 nt of the m^6^A site (‘near’) or not (‘far’). Although there was no obvious differences between these categories for most of the motifs (Supplementary file 3*,* page 2), some U-rich sequences displayed distinctive features (Figure 7A, Figure 7—figure supplement 1) that can be summarized as follows. If a transcript has m^6^A *and* ECT2 sites in close proximity, it is (1) more likely to have UNUNU/UUUUU/YYYYY sequences upstream of the m^6^A site than targets with distantly located ECT2-binding sites or than non-ECT2 targets; (2) less likely to have UUUUU/URURU sequences downstream of the m^6^A site, possibly because ECT2 prefers CUCU-like sequences downstream; and (3) less likely to have URURU/URUAY-like motifs upstream of the m^6^A site. The latter observation is striking because for the specific subset of ECT2-bound m^6^A sites with URURU/URUAY upstream of m^6^A, these sequences tend to crosslink to ECT2, as seen by the enrichment spike at ECT2 crosslink sites (Figure 5D*,* Figure 7—figure supplement 1, bottom panels). Although these two results seem contradictory at first glance, they may be reconciled by a model in which a URURU/URUAY-binding protein would compete with ECT2 for binding adjacent to m^6^A. If that protein is absent, ECT2 may bind to the site, potentially via its IDR, to stabilize the low-affinity YTH-m^6^A interaction and crosslink efficiently due to the U-content. Conversely, if occupied by the alternative interacting protein, the site might repel ECT2 (see Discussion and Figure 7B).

**Figure 7—figure supplement 1.**
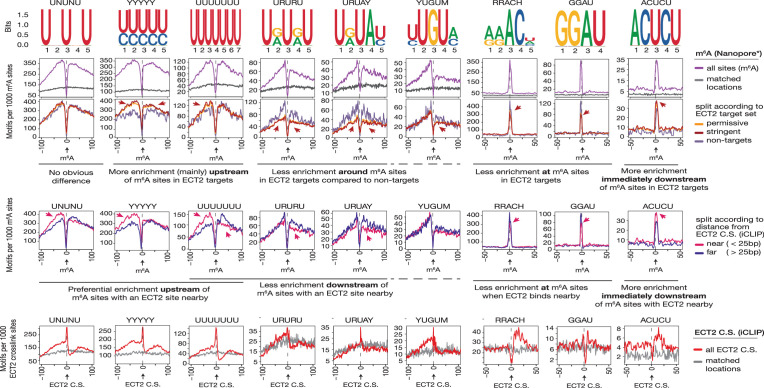
Motif preferences around m^6^A sites according to ECT2 binding. From top to bottom: (1) motif logos for derived position weight matrices (PWMs); (2) distance-based enrichment of motifs at and around m^6^A-Nanopore* sites, plotted as motif counts per 1000 m^6^A sites (purple lines). Gray lines indicate the enrichment in a location-matched background set as in Figure 5D; (3) same as in (2) with sites split according to whether they sit on ECT2 targets; (4) same as in (2) with sites split according to the distance from the nearest ECT2 crosslink site (for ECT2-iCLIP targets only); (5) motif counts per 1000 iCLIP-binding sites, as a function of distance from the iCLIP position, showing all sites against matched background sites (gray lines). Motifs are ordered according to the different relative enrichment upstream or downstream m^6^A sites according to ECT2 binding (see text below panels 3 and 4). * Parker et al., 2020. Figure 7—figure supplement 1—source data 1.High quality image file.

**Figure 7—figure supplement 2.**
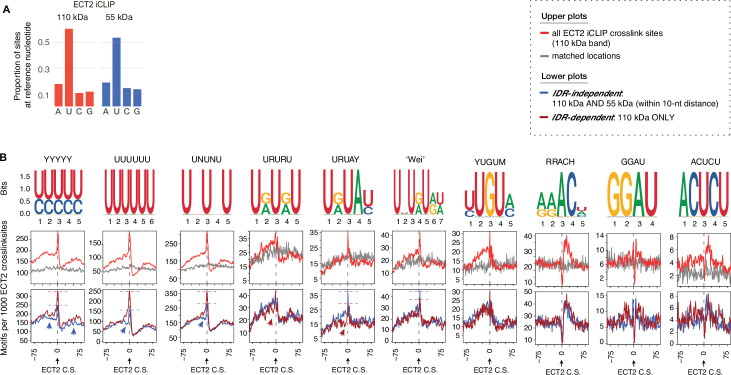
Dependency of the ECT2 intrinsically disordered region (IDR) for motif enrichment. (**A**) Proportion of ECT2-iCLIP sites at each nucleotide for the 110kDa and 55kDa bands. (**B**) From top to bottom: (1) motif logos for derived position weight matrices (PWMs); (2) distribution of motifs per 1000 ECT2-iCLIP crosslink sites (red) or matched-locations background (gray), ±75 nt of the site; (3) motifs per 1000 ECT2-iCLIP crosslink sites, split according to whether they are found in libraries from both 110kDa and 55kDa bands (’IDR-independent’) or exclusively (distance >10 nt) in the 110kDa band (’IDR-dependent’). Figure 7—figure supplement 2—source data 1.High quality image file.

### The N-terminal IDR of ECT2 is involved in preferential crosslinking at U-rich sequences and in URURU-repulsion immediately upstream m^6^A sites

We reasoned that insights into contacts between ECT2 and mRNA may be gained by analysis of the iCLIP libraries prepared with the ‘YTH-mCherry’ truncation devoid of the N-terminal IDR (‘55-kDa band’) compared to the full-length ECT2-mCherry (‘110-kDa band’) (Figure 3, Figure 3—figure supplements 2–4). Initial inspection of the distribution of ECT2 peaks relative to Nanopore-m^6^A sites showed that the 5′–3′ asymmetry observed with full-length ECT2 was largely reduced with the truncated protein (Figure 7C), as was the bias towards uridines (Figure 7—figure supplement 2A). These observations suggest that the IDR indeed is implicated in binding to U-rich regions upstream of m^6^A. We next split the full-length ECT2 iCLIP peaks according to whether they are present in libraries from both full-length and truncated forms (‘IDR-independent’) or exclusively in the full-length (‘IDR-dependent’) (distance >10 nt) and plotted the enrichment of the studied motifs relative to the crosslink site (Figure 7D, Figure 7—figure supplement 2B; Supplementary file 3, page 2). UUUUUU/UNUNU-like motifs were more enriched at and immediately upstream of IDR-dependent crosslink sites relative to the IDR-independent ones, supporting preferential crosslinking of the IDR to Us in this region. Remarkably, the exact opposite was true for URURU/URUAY motifs that showed modest depletion 5′ to IDR-dependent crosslink sites relative to their IDR-independent counterparts (Figure 7D). These observations are consistent with a model of an RNA-binding protein competing with the ECT2 IDR for interaction with upstream URURU/URUAY motifs (Figure 7B).

## Discussion

### Methodology for mapping protein-RNA interactions in plants

Our work establishes experimental and computational approaches to implement HyperTRIBE for unbiased and sensitive mapping of direct targets of RNA-binding proteins in plants. Two points are particularly relevant in this regard. First, the examples studied here show that stable transgenic expression of *Dm*ADARcd does not lead to detrimental phenotypes, perhaps because of the generally low editing proportions obtained in vivo. Second, the rigorous statistical approach developed to call editing sites makes HyperTRIBE powerful, despite the low editing proportions observed. We also note that ECT2 is well suited to verify that HyperTRIBE mostly recovers directly bound target RNAs because of the possibility to cross-reference the data with independently obtained m^6^A maps (Parker et al., 2020). The combination of iCLIP and HyperTRIBE for unbiased mapping of targets proved particularly attractive for at least two reasons. First, the convergence on overlapping target sets by orthogonal methods strengthens the confidence that the identified targets are biologically meaningful. Second, HyperTRIBE, especially with the novel computational approach for calling of editing sites (Rennie et al., 2021), offers higher sensitivity than iCLIP, while iCLIP is unmatched in providing information on binding sites within target RNAs. It is possible that better positional information on binding sites may be obtained from HyperTRIBE data using maximal editing proportions rather than statistical significance as the parameter to call editing sites. Indeed, recent work on the use of HyperTRIBE to identify targets of the RNA-binding protein MUSASHI-2 (MSI-2) in leukemic stem cells recovered the known MSI-2-binding site as enriched around editing sites in targets (Nguyen et al., 2020). Nonetheless, our data shows that highly edited sites match the ADAR substrate consensus site better than lowly edited sites, suggesting that site proximity to ADAR is not the only determinant of editing proportions. Finally, our work also clearly indicates that FA-CLIP, now used in at least two studies involving YTH domain proteins (Wei et al., 2018; Song et al., 2021), is not a recommendable technique as it recovers many false positives and fails to include many genuine targets. Thus, with the possible exception of cases in which evidence for indirect association is specifically in demand, such as the recent study in human cells of mixed tailing of viral RNA by the cellular terminal nucleotidyl transferase TENT4 (Kim et al., 2020), FA-CLIP should not be used for identification of RNAs associating with a particular RNA-binding protein of interest.

### Core elements in m^6^A writing: DRACH, GGAU, and U/Y-rich motifs

Our analyses of motif enrichments around m^6^A and ECT2 crosslink sites clarify roles of previously reported motifs and uncover new motifs of importance in m^6^A writing and ECT2 binding. Since m^6^A is a prerequisite for ECT2 binding, any analysis of determinants of ECT2 binding must consider determinants of *N6*-adenosine methylation separately. Three conclusions stand out from our analysis in this regard. First, the major *N6*-adenosine methylation site is DRACH, consistent with conclusions from multiple other studies. Second, GGAU is a minor *N6*-adenosine methylation site, as seen by its enrichment directly at m^6^A sites. Third, m^6^A occurs in DRACH/GGAU islands embedded in U-rich regions. Such U-rich regions around m^6^A sites emerged from sorting of methylated from non-methylated transcripts by machine learning as being of similar importance for recognition of m^6^A-containing transcripts from sequence features as DRACH and GGAU at m^6^A sites, suggesting their implication in MTA/MTB-catalyzed adenosine methylation (Figure 6C). This, in turn, may also explain the pronounced 3′-UTR bias of m^6^A occurrence as extensive poly-U and poly-pyrimidine tracts are rare in coding regions (Figure 5D, second row on the right-most column; Supplementary file 3, page 1). As a special case in this context, our analyses suggest a simple explanation for the tendency of m^6^A to occur at stop codons. UAA and UGA correspond to DRA, increasing the frequency of occurrence of DRACH directly at stop codons (Figure 5D*,* second row on the left-most column), many of which have adjacent U-rich elements in the 3′-UTRs. We note that the observed pattern is in agreement with a role of the poly(U)-interacting proteins RBM15A/B associated with the mammalian methyltransferase complex in guiding methylation (Patil et al., 2016). Whether a similar mechanism operates in plants, potentially via the distant RBM15A/B homologue FPA (Arribas-Hernández and Brodersen, 2020), remains to be investigated.

### Reading of DR(m^6^A)CH in 3′-UTRs of target mRNAs by ECT2

It is a major conclusion of the present work that ECT2 binds to m^6^A predominantly in the DR(m^6^A)CH sequence context in vivo, consistent with reading of m^6^A written by the conserved nuclear MTA/MTB methyltransferase. This key conclusion refutes the claim by Wei et al., 2018 that ECT2 binds to the supposedly plant-specific m^6^A-containing sequence motif URU(m^6^A)Y, and it thereby reconciles knowledge on m^6^A-YTHDF axes in plants specifically and in eukaryotes more broadly. The phenotypic similarity of plants defective in MTA/MTB writer and ECT2/ECT3/ECT4 reader function is now coherent with the locations of MTA/MTB-written m^6^A and ECT2-binding sites transcriptome-wide, and it is now clear that plants do not constitute an exception to the general biochemical framework for eukaryotic m^6^A-YTHDF function in which YTHDF proteins read the m^6^A signal written by the MTA/MTB methyltransferase.

### The role of U-rich motifs 5′ to m^6^A sites in ECT2 binding: direct interaction of the IDR of ECT2 with mRNA

The pronounced protease sensitivity of IDRs, leading to limited proteolysis of ECT2 upon cell lysis after in vivo crosslinking, allowed us to extract information on the mode of ECT2-RNA binding from different observations, all converging on the conclusion that the IDR of ECT2 participates in RNA binding. First, RNA complexes with YTH-mCherry were 5′-labeled by polynucleotide kinase much more efficiently than RNA complexes with full-length ECT2-mCherry, indicating that the IDR limits accessibility to the 5′ of bound mRNAs. Second, in contrast to the m^6^A-binding-deficient YTH^W464A^-mCherry truncation, the full-length ECT2^W464A^-mCherry mutant retained an enrichment of crosslink sites in 3′-UTRs. Third, crosslinks specific to the IDR (i.e., observed only with full-length ECT2-mCherry-RNA complexes, but not with YTH-Cherry-RNA complexes), could be assigned and have two notable properties. They are mainly 5′ to m^6^A sites, and thereby cause a conspicuously asymmetric distribution of ECT2 crosslink sites around m^6^A sites, not seen with crosslinks to the YTH-mCherry fragment. In addition, the IDR-specific crosslinks are specifically enriched in U-rich elements of the type UUUUUU and UNUNU immediately upstream. Taken together, these observations suggest that the IDR of ECT2 participates in locating ECT2 to 3′-UTRs by association with U-rich elements. Thus, ECT2, and perhaps YTHDF proteins more generally given their highly similar YTH domains, appears to bind RNA through multivalent interactions among which the YTH domain is responsible for m^6^A binding, and the IDR is responsible for interaction with adjacent elements. We note that the notion of RNA interaction by IDRs has precedent (Corley et al., 2020), is consistent with the modest affinity of isolated YTHDF domains for m^6^A-containing oligonucleotides (Patil et al., 2018), and is reminiscent of the recent demonstration that transcription factors use their globular DNA-binding domains to recognize core sequence elements of promoters, and their IDRs to provide additional DNA contacts, contributing to specificity (Brodsky et al., 2020). Similarly, it is possible that diverging IDRs among YTHDF paralogs could confer target specificity via binding to distinct motifs in the vicinity of m^6^A sites, such that specific YTHDF-target mRNA repertoires could exist even for YTHDF proteins coexpressed in the same cells. Finally, we stress that although our data point to an important role of the IDR in RNA binding, it does not in any way suggest that this is the only function of the IDR, and protein-protein interactions involving the IDR are likely to be key to understanding YTHDF function molecularly.

### URUAY as sites of competitive interaction between ECT2 and other RNA-binding proteins

Despite the conclusions that URUAY does not contain m^6^A in *Arabidopsis*, and that ECT2 binds to DR(m^6^A)CH, our detailed analysis of sequence motifs enriched around m^6^A and ECT2 iCLIP crosslink sites shows that additional motifs, including URUAY, are likely to be implicated in m^6^A reading by ECT2, even if not directly. In contrast to other m^6^A-proximal, pyrimidine-rich sequences (e.g., UNUNU, YYYYY) that may be of importance for both m^6^A writing and ECT2 binding, URUAY appears to have ties more specifically to ECT2 binding thanks to three properties. (1) When present 5′ to m^6^A sites, it crosslinks to ECT2, suggesting that some part of the protein can be in contact with URUAY. (2) URUAY is more enriched close to m^6^A sites for which there is no evidence of ECT2 binding, suggesting that it weakens ECT2 binding. This latter point is also consistent with the distinction of ECT2-bound from non-ECT2-bound m^6^A sites by machine learning that did not find URUAY to be of importance for ECT2-bound sites. (3) The URUAY enrichment 5′ to ECT2 crosslink sites is observed only when crosslinks to both full-length protein and the YTH-mCherry fragment are considered (IDR-independent), but disappears when crosslinks specific to the full-length protein (IDR-dependent) are analyzed. Although these observations may be explained by multiple scenarios, we find a simple, yet at present speculative, model attractive: URUAY may be a site of competition between the IDR of ECT2 and another, as yet unknown, RNA-binding protein. Such a competing factor could in theory be another YTHDF protein using higher-affinity IDR-URUAY contacts than ECT2 to achieve competitive binding. Many other possibilities exist, however. For example, it is intriguing that URUAY resembles part of a Pumilio-binding site (Hafner et al., 2010; Huh et al., 2013) as it raises the tantalizing possibility of functional interaction between YTHDF and Pumilio proteins. In any event, the functional dissection of the URUAY element in m^6^A reading now constitutes a subject of major importance, emphasized by the broad conservation of its enrichment around m^6^A sites across multiple plant species, including rice (Li et al., 2014a; Zhang et al., 2019), maize (Luo et al., 2020; Miao et al., 2020), tomato (Zhou et al., 2019), and *Arabidopsis* (Miao et al., 2020).

## Materials and methods

**Key resources table:** 

Reagent type (species) or resource	Designation	Source orreference	Identifiers	Additional information
Gene (*Arabidopsis thaliana*)	*ECT2*	TAIR10	AT3G13460	*EVOLUTIONARILY CONSERVED C-TERMINAL REGION 2*
Gene (*Arabidopsis thaliana*)	*ECT3*	TAIR10	AT5G61020	*EVOLUTIONARILY CONSERVED C- TERMINAL REGION 3*
Gene (*Arabidopsis thaliana*)	*ECT4*	TAIR10	AT1G55500	*EVOLUTIONARILY CONSERVED C- TERMINAL REGION 4*
Gene (*Drosophila melanogaster*)	ADAR Isoform N	Genebank, FlyBase, NCBI	CG12598 NM_001297862	Adenosine deaminase acting on RNA
Strain (*Escherichia coli*)	DH5α	NEB	Cat. # 18258012	MAX Efficiency DH5α Competent Cells
Strain (*Agrobacterium tumefaciens*)	GV3101	Koncz and Schell, 1986		
Genetic reagent (*A. thaliana*)	SALK_002225 C (*ect2-1*)	NASC	N657472 N2110120	
Genetic reagent (*A. thaliana*)	*te234* (*ect2-1/ect3-1/ect4-2*)	Arribas-Hernández et al., 2018	N2110132	Donated to NASC and ABRC
Genetic reagent (*A. thaliana*)	*ECT2pro:FLAG-DmADAR* ^*E488Q*^_*cd*_ *-ECT2ter*	This paper (see Methods)		Seed requests to pbrodersen@bio.ku.dk
Genetic reagent (*A. thaliana*)	*ect2-1/ECT2pro:ECT2-FLAG-DmADAR* ^*E488Q*^_*cd*_ *-ECT2ter*	This paper (see Methods)		Seed requests to pbrodersen@bio.ku.dk
Genetic reagent (*A. thaliana*)	*te234/ECT2pro:ECT2-FLAG-DmADAR* ^*E488Q*^_*cd*_ *-ECT2ter*	This paper (see Methods)		Seed requests to pbrodersen@bio.ku.dk
Genetic reagent (*A. thaliana*)	*ect2-1/ECT2pro: ECT2-mCherry-ECT2ter*	Arribas-Hernández et al., 2018; Arribas-Hernández et al., 2020	N2110839 N2110840	Donated to NASC and ABRC
Genetic reagent (*A. thaliana*)	*ect2-1/ECT2pro: ECT2* ^*W464A*^ *- mCherry-ECT2ter*	Arribas-Hernández et al., 2018; Arribas-Hernández et al., 2020	N2110841 N2110842	Donated to NASC and ABRC
Genetic reagent (*A. thaliana*)	*ect2−1/ ECT2pro:3xHA- ECT2-ECT2ter*	This paper (see Methods)		Seed requests to pbrodersen@bio.ku.dk
Genetic reagent (*A. thaliana*)	*ect2−1/ ECT2pro:3xHA-ECT2* ^*W464A*^ *-ECT2ter*	This paper (see Methods)		Seed requests to pbrodersen@bio.ku.dk
Genetic reagent (*D. melanogaster*)	Canton-S	Bloomington *Drosophila* Stock Center	BDSC:64,349	Used to extract RNA and produce cDNA for cloning
Antibody	anti-FLAG (mouse monoclonal)	Sigma-Aldrich	A8592	Used for WB (1:1000)
Antibody	anti-mCherry (rabbit polyclonal)	Abcam	ab183628	Used for WB (1:1000)
Antibody	anti-HA (mouse monoclonal)	Abnova	12CA5	Used for WB (1:2000)
Antibody	RFP-Trap RFP Nanobody/V_H_H coupled to agarose (recombinant, monoclonal)	ChromoTek	Cat. # rta-20	Used for IP (20 μL of beads for 4 g of tissue in 6 mL of buffer)
Antibody	Anti-HA Affinity Matrix from IgG1 3 F10 (rat, monoclonal)	Roche	Cat. # 11815016001	Used for IP (10 μL of beads for 500 mg of tissue in 750 μL of buffer)
Recombinant DNA reagent	pCAMBIA3300U (plasmid)	Nour-Eldin et al., 2006		Used for cloning
Commercial assay or kit	pGEM -T Easy (plasmid and cloning kit)	Promega	Cat. # A1360	Used for cloning
Commercial assay or kit	KAPAHiFi HotStart Uracil + Kit	Roche	Cat. # 7959079001	Used for cloning
Commercial assay or kit	AccuPrime Supermix I	Invitrogen	Cat. # 12342–010	Used for iCLIP library preparation
Peptide, recombinant protein	Uracil-DNA Glycosylase (USER enzyme)	NEB	Cat. # M5505L	Used for cloning
Peptide, recombinant protein	Turbo DNase	Ambion	Cat. # AM2238	Used for CLIP
Peptide, recombinant protein	RNase I	Ambion	Cat. # AM2294	Used for CLIP
Peptide, recombinant protein	T4 Polynucleotide Kinase (PNK)	ThermoFisher Scientific	Cat. # EK0031	Used for iCLIP library preparation
Peptide, recombinant protein	T4 RNA Ligase I, High Concentration	NEB	Cat. # M0437M	Used for iCLIP library preparation
Peptide, recombinant protein	Proteinase K	Roche	Cat. # 3115887001	Used for iCLIP library preparation
Peptide, recombinant protein	Superscript III Reverse Transcriptase	Invitrogen	Cat. # 18080–093	Used for iCLIP library preparation
Peptide, recombinant protein	CircLigase II ssDNA Ligase	Epicentre	Lucigen Cat. # CL9021K	Used for iCLIP library preparation
Peptide, recombinant protein	BamHI (Fast Digest)	ThermoFisher Scientific	Cat. # FD0054	Used for iCLIP library preparation
Chemical compound, drug	cOmplete protease inhibitor cocktail	Roche	Cat. # 11697498001	Used for CLIP
Chemical compound, drug	Protease inhibitor cocktail for plant cell extracts	Sigma	Cat. # P9599	Used for CLIP
Chemical compound, drug	Glufosinate-ammonium (PESTANAL)	Sigma	Cat. # 45520 77182-82-2	Used for selection of transgenic lines
Sequence-based reagent	Pre-adenylated adapter for iCLIP (3’-RNA linker)	Huppertz et al., 2014	L3-App	rAppAGATCG GAAGAGCGGT TCAG/ddC/
Sequence-based reagent	iCLIP RT-primers (Two-part cleavable DNA adapters complementary to the 3’ RNA linker)	Huppertz et al., 2014	Rt1clip-Rt12clip	Used for iCLIP library preparation (seq: )
Sequence-based reagent	USER and site-directed mutagenesis primers	This paper (Appendix)		Used for cloning. Sequences are in the Appendix
Sequence-based reagent	Primers for detection of point mutations	This paper (Appendix)		Used for cloning. Sequences are in the Appendix
Software, algorithm	R	https://www.R-project.org/		Used for data analyses
Software, algorithm	hyperTRIBE**R**	Rennie et al., 2021; https://github.com/sarah-ku/hyperTRIBER; https://github.com/sarah-ku/targets_arabidopsis		Used for calling significant ADAR-edited sites. Contact: sarah@binf.ku.dk
Software, algorithm	*trimmomatic*	Bolger et al., 2014		Used for trimming RNAseq-reads
Software, algorithm	STAR	Dobin et al., 2013		Used for mapping RNAseq-reads
Software, algorithm	Salmon	Patro et al., 2017		Used for transcript quantification
Software, algorithm	SAMtools *mpileup*	Li et al., 2009		Used to count nt-mismatches
Software, algorithm	*rtracklayer*	Lawrence et al., 2009		Used to retrieve sequences
Software, algorithm	*ggseqlogo*	Wagih, 2017		Used to generate motif logos
Software, algorithm	*Hmisc*	https://github.com/harrelfe/Hmisc/		Used for expression-based binning
Software, algorithm	*fastqc*	https://www.bioinformatics.babraham.ac.uk/projects/fastqc/		Used for quality control
Software, algorithm	*cutadapt*	Martin, 2011		Used for trimming of iCLIP reads
Software, algorithm	*flexbar*	Roehr et al., 2017		Used for demultiplexing iCLIP reads
Software, algorithm	PureCLIP	Krakau et al., 2017		Used for calling iCLIP peaks
Software, algorithm	*GenomicRanges*	Lawrence et al., 2013		Used to retrieve short sequences
Software, algorithm	‘Distributions of motifs per 1,000 sites over distance’	This paper https://github.com/sarah-ku/targets_arabidopsis		Used to calculate motif distributions around m^6^A/iCLIP. Contact: sarah@binf.ku.dk
Software, algorithm	*ggplot2*	https://ggplot2.tidyverse.org		Used to generate plots
Software, algorithm	*bedtools*	Dale et al., 2011; Quinlan and Hall, 2010		Used to filter and clean iCLIP data
Software, algorithm	Homer	Heinz et al., 2010		Used for de novo motif discovery
Software, algorithm	FIMO	Grant et al., 2011		Used to detect motif occurrences
Software, algorithm	*gbm*	https://github.com/gbm-developers/gbm		Used for random forest analysis
Software, algorithm	*pROC*	Robin et al., 2011		Used to estimate predictive score of RF
Software, algorithm	IGV (Integrative Genomics Viewer)	Robinson et al., 2011		Used to show genomic data

All data analyses were carried out using TAIR 10 as the reference genome and Araport11 as the reference transcriptome. Unless otherwise stated, data analyses were performed in R (https://www.R-project.org/) and plots generated using either base R, IGV (for genomic data) (Robinson et al., 2011), or *ggplot2* (https://ggplot2.tidyverse.org).

### Definitions of experiment, biological replicates, and technical replicates

We use the term ‘biological replicate’ in the following way: plants were grown at the same time, under the same conditions, but in separate plates. Each sample replicate contains pools of seedlings prepared in such a way that no two replicates contain seedlings grown on the same plates. This sampling ensures that plate-to-plate variation in growth conditions, if any, will have an effect on measurements of gene expression within a single genotype, and hence minimize the risk that any differences due to such variation are called as significant in comparisons between genotypes. ‘Technical replicates’ are understood to be independently conducted measurements using the same technique on the same biological material (e.g., on one biological replicate as defined above). Technical replicates were not carried out in this study, and the term ‘replicate’ refers to biological replicate as defined above. In our definition, an ‘experiment’ results in generation and comparison of measurements arising from multiple biological replicates of different biological entities, in the present case often *Arabidopsis* seedlings differing in genotype with respect to the genes *ECT2*, *ECT3,* and *ECT4*. Thus, repetition of an experiment in our definition entails generation and analysis of the required biological replicates at different points in time.

### Plant material

All lines used in this study are in the *Arabidopsis thaliana* Col-0 ecotype. The mutant alleles or their combinations – *ect2-1* (SALK_002225) (Arribas-Hernández et al., 2018; Scutenaire et al., 2018; Wei et al., 2018)*, ect3-1* (SALKseq_63401)*, ect4-2* (GK_241H02), and *ect2-1/ect3-1/ect4-2* (*te234*) (Arribas-Hernández et al., 2018) – have been previously described. The transgenic lines expressing *ECT2pro:ECT2-mCherry-ECT2ter*, *ECT2pro:ECT2^W464A^-mCherry-ECT2ter, ECT2pro:3xHA-ECT2-ECT2ter*, or *ECT2pro:3xHA-ECT2^W464A^-ECT2ter* in the *ect2-1* background have also been described or generated by floral dip in additional mutant backgrounds using the same plasmids and methodology (Arribas-Hernández et al., 2018; Arribas-Hernández et al., 2020).

### Growth conditions

Seeds were surface-sterilized by 2 min incubation in 70% EtOH plus 10 min in sterilizing solution (1.5% NaOCl, 0.05% Tween-20) and 2 H_2_O washes. After 2–5 days of stratification at 4°C in darkness, seeds were germinated and grown on plates containing Murashige and Skoog (MS)-agar medium (4.4 g/L MS, 10 g/L sucrose, 10 g/L agar) pH 5.7 at 20°C, receiving ~70 μmol m^–2^ s^–1^ of light in a 16 hr light/8 hr dark cycle as default. For HyperTRIBE and iCLIP experiments, the plates were placed vertically to facilitate root harvesting. MS-agar media for HyperTRIBE T2 seedlings was supplemented with 7.5 mg/L of glufosinate ammonium (Sigma) to select plants expressing the ADAR-containing transgenes. To assess phenotypes of adult plants, ~8-day-old seedlings were transferred from horizontal MS plates (4.4 g/L MS, 10 g/L sucrose, 8 g/L agar; pH 5.7) to soil and maintained in Percival incubators under 16 hr light/8 hr dark cycles, 21°C day/18°C night temperature, and ~100 μmol m^–2^ s^–1^ light intensity. We used Philips fluorescent tubes TL-D 90 De Luxe 36 W as light source.

### Generation of transgenic lines for HyperTRIBE

We employed USER cloning (Bitinaite and Nichols, 2009) to generate *ECT2pro:ECT2-FLAG- DmADAR^E488Q^cd-ECT2ter* and *ECT2pro:FLAG-DmADAR^E488Q^cd-ECT2ter* constructs in pCAMBIA3300U (pCAMBIA3300 with a double PacI USER cassette inserted between the *Pst*I-*Xma*I sites at the multiple cloning site; Nour-Eldin et al., 2006). Fragments containing *ECT2* gDNA sequences were amplified by PCR (KAPA HiFi Hotstart Uracil + ReadyMix, Roche) from plasmids previously generated in our lab (Arribas-Hernández et al., 2018). The *FLAG-DmADAR^E488Q^cd* fragment was produced in the same way using a pGEM-T Easy (Promega) plasmid containing FLAG-*DmADAR^E488Q^cd* as template, previously subcloned to introduce the E488Q hyperactive mutation by site-directed mutagenesis (QuickChange, Agilent Technologies) with primers LA729-LA730 (Phusion HF DNA Polymerase, NEB). The E488Q mutation was detected by *NlaIII* (ThermoFisher) digestion of the PCR reaction (DreamTaq, ThermoFisher) obtained with primers LA660-LA735. Of note, the *FLAG* and *DmADARcd* sequences had been previously glued together by USER cloning to produce *AGO1pro:FLAG-DmADARcd-AGO1ter* in pCAMBIA3300U for unrelated purposes (unpublished work), and subsequently amplified by PCR with primers LA696-615 for introduction into pGEM-T Easy. To build *AGO1pro:FLAG-DmADARcd-AGO1ter* in the first place, the catalytic domain of the ADAR deaminase isoform N (Y268-E669) was amplified from cDNA of *D. melanogaster* Canton-S wild-type flies and larvae with USER primers MVUSER12-22. The rest of the fragments were amplified from *pCAMBIA3300U AGO1pro:FLAG-AGO1-AGO1ter* (Arribas-Hernández et al., 2016) with primers MVUSER1-11 and MVUSER23-6.

USER primers to amplify all fragments were designed to create overhangs compatible with either the *PacI* USER cassette present in the pCAMBIA3300U plasmid or the flanking sequences of the neighboring fragments. All primer sequences, their combinations to produce PCR fragments, and the arrangement of the fragments for USER cloning can be found in Appendix 1.

Kanamycin-resistant colonies of *Escherichia coli* DH5α (NEB) transformed with the constructs were analyzed by restriction digestion and sequencing prior introduction of the plasmids in *Agrobacterium tumefaciens* GV3101 (Koncz and Schell, 1986) for plant transformation.

*Arabidopsis* stable transgenic lines were generated by floral dip transformation (Clough and Bent, 1998) of Col-0 WT, *ect2-1*, or *te234*, and selection of primary transformants (T1) was done on MS-agar plates supplemented with glufosinate-ammonium (Sigma) (10 mg/L). We selected five independent lines of each type based on segregation studies (to isolate single T-DNA insertions), phenotypic complementation (in the *te234* background), and transgene expression levels assessed by FLAG western blot.

### Western blotting

Protein extraction from 10-day-old seedlings and western blotting with FLAG, HA, and mCherry antibodies were done as previously described (Arribas-Hernández et al., 2018). Loading was documented by amido black, Coomassie, or Ponceau staining of the total protein on the membrane.

### RNA extraction and library preparation for HyperTRIBE

We extracted total RNA from manually dissected root tips and apices (removing cotyledons) of five independent lines (10-day-old T2 seedlings) of each of the lines used for ECT2-HT to use as biological replicates. The tissue was flash-frozen in liquid nitrogen and ground into a fine powder using liquid nitrogen-cooled adaptors in a tissue homogenizer. For RNA extraction, we added 1 mL of TRI Reagent (Sigma) to the frozen tissue (<100 mg), mixed quickly by vortexing, added 0.2 mL of chloroform, and separated the two resulting phases by vigorous shaking and 10 min centrifugation at 4°C. The RNA was then precipitated from the aqueous phase for 30 min at room temperature with 1 volume of isopropanol. RNA pellets were solubilized in 300 μL of H_2_O to remove polysaccharides through a mild precipitation by addition of 1/10 vol. 99% EtOH and 1/30 vol. of 3 M NaOAc (pH 5.2) and incubation on ice for 30 min. After 15 min of full-speed centrifugation at 4°C to pellet polysaccharides, we re-precipitated the RNA from the supernatant with 2,5× vol. 99% EtOH and 1/10 vol. of 3 M NaOAc (pH 5.2), washed the pellet two times with 70% EtOH, and resuspended in 20–40 μL of H_2_O. This highly pure total RNA was then used to produce mRNA libraries through enrichment of mRNA with oligo(dT) beads (18-mers), random fragmentation, cDNA synthesis with random hexamers, custom second-strand synthesis (Illumina), terminal repair, A-ligation and sequencing adaptor ligation, size selection (250–300 bp insert), and PCR enrichment. The libraries were prepared and sequenced (Illumina PE150, Q30 ≥ 80%) as a service from Novogene.

The entire HyperTRIBE experiment was done once.

### HyperTRIBE data analysis

Significant differentially edited sites between *ECT2-FLAG-ADAR* (fusion) and *FLAG-ADAR* (control) samples for ECT2 HyperTRIBE (ECT2-HT) were called according to the hyperTRIBE**R** pipeline (Rennie et al., 2021). First, reads were trimmed using *trimmomatic* (Bolger et al., 2014) and mapped to the *Arabidopsis* genome (TAIR10) using STAR (Dobin et al., 2013), according to parameters suggested in a previous HyperTRIBE analysis (Xu et al., 2018). All HyperTRIBE samples were also quantified using Salmon (Patro et al., 2017), with appropriate settings for pair-end sequencing and non-stranded library setup and based on the transcriptome for Araport11 (Cheng et al., 2017) with manual addition of the *FLAG-ADAR* sequence. A custom Perl script based on SAMtools *mpileup* (Li et al., 2009) returned base counts for all positions where there is a mismatch from the reference in at least one sample. For running the hyperTRIBE**R** analysis pipeline, we specified that any tested position must have a putative edit in at least four of the five replicates in the *ECT2-FLAG-ADAR* samples (three of four in the case of roots since one of these samples, ‘L3,’ was deemed as low quality and subsequently removed from the significance calling pipeline). Significant hits (adjusted p-value<0.01 and log_2_FC > 1) were further filtered as follows: (1) hits that did not correspond to an A-to-G change (or a T-to-C change for the negative strand), (2) hits that were likely SNPs arising specifically in either the *ECT2-FLAG-ADAR* or *FLAG-ADAR* line manifesting in an editing proportion at or close to 1, and (3) hits where the coverage of tags at the edit base over the *ECT2-FLAG-ADAR* were fewer than 10 reads. Specific scripts for the analysis of ECT2-HT data can be found at https://github.com/sarah-ku/targets_arabidopsis.

Editing proportions were calculated as G/(A + G) (alternatively C/(U + C) for the negative strand) for all significant sites, averaged over all samples, separately for the *ECT2-FLAG-ADAR* and *FLAG-ADAR* samples. Significant sites were annotated to genes from Araport11, prioritizing the gene with the highest expression (annotated TPMs are based on Salmon quantifications of *FLAG-ADAR* control samples only) in the given tissue in the case of multiple possibilities. Possible transcripts were subsequently ordered by expression, along with gene body location along the transcript (5′-UTR, CDS, 3′-UTR).

Principal component analysis was carried out on the raw editing proportions per sample for all sites with significant evidence of editing.

For the comparison of sites between aerial tissues and roots, genes defined as commonly expressed in both types of tissues were considered in all gene-based comparisons. For significant editing site-based comparison, we directly compared sites that were common and significant to both.

To calculate correlations between editing proportions and *FLAG-ADAR* expression levels among lines, transcripts per million (TPM) mapping to *FLAG-ADAR* were extracted from quantifications from Salmon (Patro et al., 2017) and correlated with the raw editing proportions per sample, separately for the fusion and control samples. Background correlation estimates were calculated by first scrambling the order of the *FLAG-ADAR* TPM vector.

For motif identification at significant ECT2-HT sites, all sequences for bases at and -/+ 2 nt of the significant editing positions were derived from TAIR10 using the R package *rtracklayer* (Lawrence et al., 2009) in either aerial tissues or roots. A matrix of nt frequencies (A, C, G, or U) was generated, and the R package *ggseqlogo* (Wagih, 2017) was used to generate the final motif.

For the calculation of editing proportions as a function of the proportion of cells coexpressing ECT2, we first downloaded the expression matrix based on a total of 4727 individual cells from scRNA-seq in roots from Denyer et al., 2019. To estimate the relationship between coexpression of target genes with ECT2 and their average editing proportions, the expression matrix was used to calculate coexpression for each target gene as follows: (# cells expressing ECT2 AND target gene) / (# cells expressing target gene).

These proportions were then split into groups and plotted against the maximum editing proportions from HyperTRIBE in the containing genes.

### Comparative analysis of target sets and their expression bias

For expression binning, log_2_(TPM +1) values for all expressed genes in either aerial tissues, roots, or combined were split into nine bins of increasing expression, using the cut() function from the R package *Hmisc* version 4.5-0 (https://github.com/harrelfe/Hmisc/). For the proportion of target genes in every expression bin, we calculated the proportion of genes in each set (ECT2 HT/iCLIP-targets or nontargets, ECT2 FA-CLIP; Wei et al., 2018) or m^6^A sets (Parker et al., 2020) falling into each expression bin out of the total number of genes in that bin. To demonstrate expression biases in unsupported ECT2-HT target genes, the genes were further split according to whether or not they had support from m^6^A (Nanopore, miCLIP [Parker et al., 2020] and m^6^A-seq [Shen et al., 2016]).

### CLIP experiments and iCLIP library preparation

In vivo UV crosslinking of 12-day-old seedlings and construction of iCLIP libraries were optimized for ECT2-mCherry from the method previously employed for *Arabidopsis* GRP7-GFP (Meyer et al., 2017; Köster and Staiger, 2020) as follows. Crosslinked plant tissues (see details below) were finely ground in liquid nitrogen with mortar and pestle, homogenized in iCLIP buffer (50 mM Tris-HCl pH 7.5, 150 mM NaCl, 4 mM MgCl_2_, 5 mM DTT, 1% SDS, 0.25% sodium deoxycholate, 0.25% Igepal) supplemented with protease inhibitors (4 mM PMSF, 1 tablet/10 mL of Complete Protease Inhibitor Cocktail [Roche], and 1/30 vol. of Protease Inhibitor Optimized for Plant Extracts [Sigma P9599]), and cleared by centrifugation and filtration (0.45 μm pore) of the supernatant. RNP-complexes were then immunopurified with beads coupled to anti-RFP nanobodies (ChromoTek RFP-Trap in our case) for 1 hr at 4°C under constant rotation. In particular, we used 20 μL of beads for 4 g of tissue in 6 mL of iCLIP buffer for every replicate. After thorough washes with RIP-Wash Buffer (2 M urea, 50 mM Tris-HCl pH 7.5, 500 mM NaCl, 4 mM MgCl_2_, 2 mM DTT, 1% SDS, 0.5% sodium deoxycholate, 0.5% Igepal), RNP-complexes attached to the beads were subjected to treatment with DNase (Turbo DNase [Ambion], 4 U/100 μL) and RNase I (Ambion, 1 U/mL) at 37°C for 10 min, dephosphorylation of RNA 3′ ends (PNK [ThermoFisher] in pH 6.5 buffer), and 3′ RNA linker ligation (L3-App linker [Huppertz et al., 2014] and NEB HC RNA Ligase) at 16°C overnight. RNA was radioactively labeled at the 5′ end by PNK-mediated phosphorylation using γ-^32^P-ATP (20 min at 37°C). The labeled RNP complexes were subjected to SDS-PAGE and blotting on a nitrocellulose membrane (Protran BA-85). Pieces of membrane containing a size range of RNA species bound to the protein (a smear above the expected molecular weight localized by autoradiography) were excised and subjected to proteolysis (200 μg of Proteinase K [Roche] in 200 μL of PK buffer [100 mM Tris-HCl pH 7.4, 50 mM NaCl, 10 mM EDTA] for 20 min at 37°C) to release RNA bound to small peptides. The RNA was then purified with TRI-Reagent (Sigma) and used to prepare sequencing libraries through the following steps: reverse transcription (Superscript III, Invitrogen) using a two-part cleavable DNA adapter complementary to the 3′ RNA linker as primer, gel purification and size selection of cDNA (high, 120–200 nt; medium, 85–120 nt; low, 70–85 nt), circularization (CircLigase II Epicentre), relinearization (BamHI), and PCR amplification (AccuPrime Supermix I, Invitrogen). All steps were performed as described by Huppertz et al., 2014, and the amount of cycles in the final PCR was optimized to the amount of cDNA in each sample.

Notice that we introduced a few modifications in the original protocol (Köster and Staiger, 2020) to account for (1) low abundance of ECT2 compared to AtGRP7. To obtain enough RNA, we increased the crosslinking energy and irradiated 12-day-old seedlings with 2000 mJ/cm^2^ of 254 nm UV light, harvesting roots and shoots (4 g of tissue per replicate) to maximize the amount of purified ECT2-mCherry. (2) ECT2 sensitivity to proteolysis. We did not pre-clear the lysates to reduce the incubation time, and we used high amounts of protease inhibitors during immunoprecipitation. (3) High molecular weight of ECT2-mCherry. Due to the size of the protein, we required longer electrophoresis time and cooling (3 hr at 180 V with the tank on ice). (4) Different RNA-binding capacity of ECT2. Based on trials, we decided to adjust the RNase I treatment to 1 U/mL, incubating for 10 min at 37°C (5 μL of RNase I [Ambion, 100 U/μL pre-diluted 1:5000] in 100 μL).

Of note, the conditions indicated here were specifically used for library preparation. Although we used the same conditions as default for CLIP experiments to assess ECT2 RNA-binding capacity, ECT2 sensitivity to proteolysis and ECT2-bound RNA sensitivity to RNase treatment, variations in buffer composition, incubation time, concentration of protease inhibitors, and/or RNase I are specified in the corresponding figure legends where necessary.

The entire iCLIP-seq experiment including three replicates of each group was done once.

### iCLIP data analysis and peak calling

Sequenced reads from all samples were investigated after each processing step with *fastqc* 0.11.5 (https://www.bioinformatics.babraham.ac.uk/projects/fastqc/). Adapters at the 3′ end were trimmed using *cutadapt* version 1.16 (Martin, 2011). The demultiplexing of the samples was performed using *flexbar* 3.4.0 with the -bk parameter to conserve the barcode information for further steps (Roehr et al., 2017). Reads with a length below 24 nucleotides were discarded. Barcodes were trimmed and saved to the *read_id* field. Processed reads were mapped to the TAIR10 genome with STAR version 2.6.0a allowing a maximum of two mismatches and soft clipping only at 3′ end (Dobin et al., 2013). PCR duplicates were removed by grouping the reads by their mapping start position. Reads with the identical start position and random barcode were removed from the samples (Python3 and pybedtools). The peak calling of uniquely mapped reads was done using PureCLIP 1.0.4, choosing the second peak-shape option to allow more broader peaks to be called (Krakau et al., 2017).

For consistency with the ECT2-HT datasets, the ECT2-iCLIP datasets were annotated using the hyperTRIBE**R** annotation (Rennie et al., 2021), using quantifications based on the average of roots and aerial tissues from *FLAG-ADAR* samples in ECT2-HT (to reflect that the ECT2-iCLIP data is based on whole seedlings).

To calculate the proportion of sites falling at each nucleotide, nucleotide sequences from the reference genome were first obtained from site coordinates for ECT2 iCLIP/m^6^A-Nanopore/ m^6^A-miCLIP using the R packages *GenomicRanges* (Lawrence et al., 2013) and *rtracklayer* (Lawrence et al., 2009). Nucleotide proportions were plotted using ggplot2 (https://ggplot2.tidyverse.org).

### Analysis of publicly available data

Single-cell expression data and marker genes associated with 15 clusters annotated to cell types in roots were downloaded from Denyer et al., 2019. Single-nucleotide resolution locations of m^6^A sites (defined according to Nanopore or miCLIP) were downloaded from Parker et al., 2020. Intervals defining m^6^A locations based on m^6^A-seq were downloaded from Shen et al., 2016, and intervals defining locations of ECT2-bound sites as determined by FA-CLIP were downloaded from Wei et al., 2018. For consistency with HyperTRIBE and ECT2-iCLIP, all sets of m^6^A or ECT2-bound sites were gene annotated using the hyperTRIBE**R** pipeline, based on genes and transcripts from Araport11.

### Motif discovery

To remove redundancy after ECT2-iCLIP peak calling, directly adjacent peaks (crosslink sites) were grouped together and only the peak with the highest pureCLIP score (dominant) was kept. The called peak position (1 nt resolution) was extended by 4 nt up- and downstream to define a ‘collapsed crosslink site’ (CSS) with length 9 nt. The center position marks the dominant called peak. The extension of the peak positions was computed using *bedtools* version 2.27.1 (Quinlan and Hall, 2010; Dale et al., 2011). The collapsed ECT2-iCLIP crosslink sites and m^6^A-Nanopore sites (Parker et al., 2020) were used to find motifs significantly enriched by Homer (Heinz et al., 2010) using a variety of window sizes, settings, and backgrounds. Motifs resulting from Homer searches were collated manually, and a range of variants of the consensus motif RRACH (e.g., RACH, DRAY, DRACH, URACH, DRACG) were also added to the list, as well as various combinations of U-rich sequences (e.g., UUUUU, UNUNU, etc.), specific motifs found to be of interest in scientific literature (e.g., URUAY [Wei et al., 2018], GGAU [Anderson et al., 2018]), and extra motifs that appeared of potential interest from manually browsing with IGV (Robinson et al., 2011) the sequence in the vicinity of iCLIP peaks (e.g., YYYYY, DRACUCU). This resulted in a final list of 48 motifs for further analysis.

### Motif analysis

For each of the 48 motifs compiled from multiple sources, a custom PWM was generated based on local sequence frequencies around ECT2-iCLIP peaks and used as input to FIMO 5.1.1 (Grant et al., 2011) to detect genome-wide occurrences. To generate PWMs, we used the formula PWMb,j = log_2_ [p(b,j)/p(b)], where p(b) is the background frequency of each nucleotide (see further down), and p(b,i) is the frequency of the nucleotides in each position j. We also included an extra small frequency count in the calculation to account for potential uncertainty in redundancies. In order to account for location-specific sequence contexts (typically 3′-UTR), each site from iCLIP or m^6^A (Parker et al., 2020) sets was assigned a random ‘matched background’ site, in a non-target gene, at the same relative location along the annotated genomic feature of the site (5′-UTR, CDS or 3′-UTR), according to a resolution of 10 bins per feature. Logos for all motifs were generated using the R packages ggplot2 (https://ggplot2.tidyverse.org) and *ggseqlogo* (Wagih, 2017). To run the calculated PWMs through FIMO, we specified background letter frequencies (A: 0.273, C: 0.165, G: 0.173, U: 0.389), a threshold of 0.05, and scanning across the full TAIR10 *Arabidopsis* genome. Sites were further filtered downstream to have a score of at least 4 – in the vast majority of cases corresponding to an exact match the (short) motif.

Distributions of motifs per 1000 sites over distance, centering on ECT2-iCLIP or m^6^A sites and the respective matched backgrounds, were generated using a custom R-script (https://github.com/sarah-ku/targets_arabidopsis) based on overlaps using *GenomicRanges* (Lawrence et al., 2013). At any given shift from the peak set, the raw number of overlaps of the motif (at any point) was calculated and normalized to give a motif count per 1000 peaks. To adjust for the potential for downstream regions overshooting the end of the 3′-UTR, at each given distance only sites that continue to overlap an annotated gene (Araport11) are counted. For some analyses, peak sets were further split according to IDR-dependency or target status as indicated.

To calculate motif enrichment over the gene body, motifs were first annotated a value according to their relative position within the gene body regions: 5′-UTR, CDS or 3′-UTR. In order to account for over-representation of counts within the CDS, due to greater sequence coverage within transcript annotations, a random background set of 10 million positions were generated from the transcript annotation file and annotated in the same way as the motif locations to obtain an expected distribution of all positions over the gene body regions. This *E*xpected distribution was used to normalize the *O*bserved distribution of each motif, and O/E values were plotted as a metagene plot over the gene. An enrichment of 1 suggests that the motif is neither over- or under-represented at that location.

### Random forest analysis (machine learning)

Called positions from either Nanopore m^6^A data (Parker et al., 2020) or ECT2 iCLIP were first reduced to remove redundant regions of multiple peaks within the same window, then paired with matched background sets (described above). Windows representing ‘at’ (±10 nt) the motif together with adjacent upstream ‘up’ and downstream ‘down’ windows of length 50 nt (resulting in total window sizes of 120 nt) around each position were annotated according to the number of each of the motifs overlapping (truncated at 10), and the final data set normalized. To create a held-out set, 1/5th of the peaks were removed from the set, and the other 4/5th were used to build a random forest model using gradient boosting (R package *gbm* version 2.1.8*;* https://github.com/gbm-developers/gbm), with settings specifying a shrinkage of 0.05, an interaction.depth of 6, cv.folds = 5, and n.trees = 2000. For each model (m^6^A Nanopore-based or ECT2 iCLIP-based), importance scores were extracted from the model and the top features were selected. The held-out data was further used to estimate the predictive score of the model by calculating the AUC (R package *pROC;* Robin et al., 2011). Two further models were run – one involving the top 10 features from the full feature model, and (only for the m^6^A-Nanopore set-up) one involving features from only DRACH and UNUNU (equating to six features in total), and AUC values were calculated and compared to that of the full feature model.

## Data Availability

All sequencing data (iCLIP-seq, HyperTRIBE, mRNA-seq, small RNA-seq) have been deposited in the European Nucleotide Archive under accession code PRJEB44359. The code specific for this article is available at GitHub https://github.com/sarah-ku/targets_arabidopsis (copy archived at swh:1:rev:ab778b60f735a07d2ef181edc5b2dfbf25153021). The following dataset was generated: BrodersenP 2021Principles of mRNA targeting and regulation via Arabidopsis YTHDF proteinsEuropean Nucleotide ArchivePRJEB44359

## Additional files

Supplementary file 1. ECT2 HyperTRIBE data.

Supplementary file 2. ECT2-mCherry iCLIP data.

Supplementary file 3. Analysis of HOMER-identified and additional motifs around ECT2-iCLIP and m^6^A sites.

Transparent reporting form

## Appendix 1

**Table:**

DNA oligonucleotides (all sequences are 5′ to 3′)	
**Cloning**	
**Constructs**	**Primer pairs (fragments**)
*ECT2pro:ECT2-FLAG-ADAR-ECT2ter* (in pCAMBIA3300-U)	LA336-695 (*ECT2pro:ECT2*), LA696-615 (*FLAG-ADAR*),LA616-337 (*ECT2ter*)
*ECT2pro:FLAG-ADAR-ECT2ter*(in pCAMBIA3300-U)	LA336-697 (*ECT2pro*), LA698-615 (*FLAG-ADAR*), LA616-337 (*ECT2ter*)
*AGO1pro:FLAG-ADAR-AGO1ter*(in pCAMBIA3300-U)	MVUSER1-11 (*AGO1pro-FLAG*), MVUSER12-22 (*ADAR*),MVUSER23-6 (*AGO1ter*)
*FLAG-ADAR* (in pGEM-T Easy)	LA696-615 (*FLAG-ADAR*)
	
**Primers for USER cloning**	
LA336.U-ECT2P.F	GGCTTAAUAAGCAACGAACCAAGGGAAGACG
LA337.ECT2T-U.R	GGTTTAAUAGGTTCTCTCGGCTTCTTTGAC
LA615.dADAR/ECT2T.R	AGTTATUCGGCAAGACCGAACTCGTC
LA616.dADAR/ECT2T.F	AATAACUAAGAGGATGGTGTCGCTC
LA695.ECT2/FLAG.R	ATCGCAACCAUTTGCCACCACATCG
LA696.ECT2/FLAG.F	ATGGTTGCGAUTACAAGGATGACGATGAC
LA697.ECT2P/FLAG.R	AATCCAUGAGAGGAGATTCGACAAACAAAG
LA698.ECT2P/FLAG.F	ATGGATUACAAGGATGACGATGAC
MVUSER1.F	GGCTTAAUCTATCCAAATTCCAAACCATACG
MVUSER6.R	GGTTTAAUGATTCTGTCGATTGCTTTGCTGG
MVUSER11.R	ATTGGACTGUACTTGTCATCGTCATCCTTG
MVUSER12.F	ACAGTCCAAUGGTGGTGCCACAG
MVUSER22.R	ACTGCGGCAGCUCATTCGGCAAGACCGAACTCG
MVUSER23.F	AGCTGCCGCAGUTGATTCACCCTCTATCTATCTTTATGACC
	
**Primers for site-directed mutagenesis (QuickChange**)	
LA729.dADAR_E488Q_QC.F	CAAAATCGAGTCCGGTCAGGGGACGATTCCAG
LA730.dADAR_E488Q_QC.R	CTGGAATCGTCCCCTGACCGGACTCGATTTTG
	
**Primers for detection of point mutations**	
LA660.dADAR_E488Q_CP(NlaIII).F	CGAAAACGACACTGGTGTTG
LA735.dADAR_E488Q_CP(NlaIII).R	GCTTTTCACTGGAATCGTCCCAT
