# Supplementary material for: Principles of mRNA targeting via the Arabidopsis m6A-binding protein ECT2
Source: eLife. 2021 Sep 30;10:e72375. doi: 10.7554/eLife.72375 (PMC8796052; doi:10.7554/eLife.72375)

Figure 1—figure supplement 1—SourceData1

Figure panel

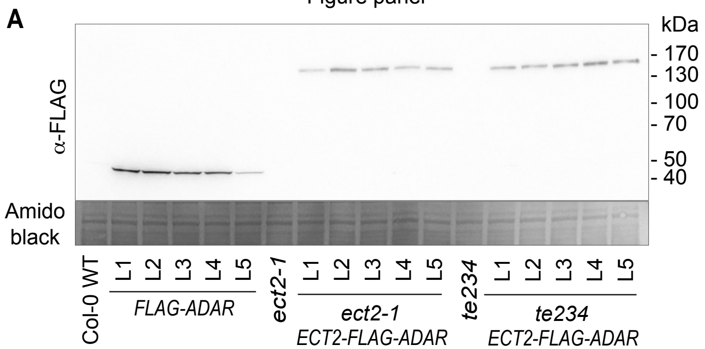

Notice that the loading order has been mirrored left-to-right to restore the original loading order (with the WT control first) that becomes mirrored during the blotting procedure°

Dotted outlines indicate the cropping applied to the figure

Enhanced Chemiluminescence signal (α-FLAG antibody)

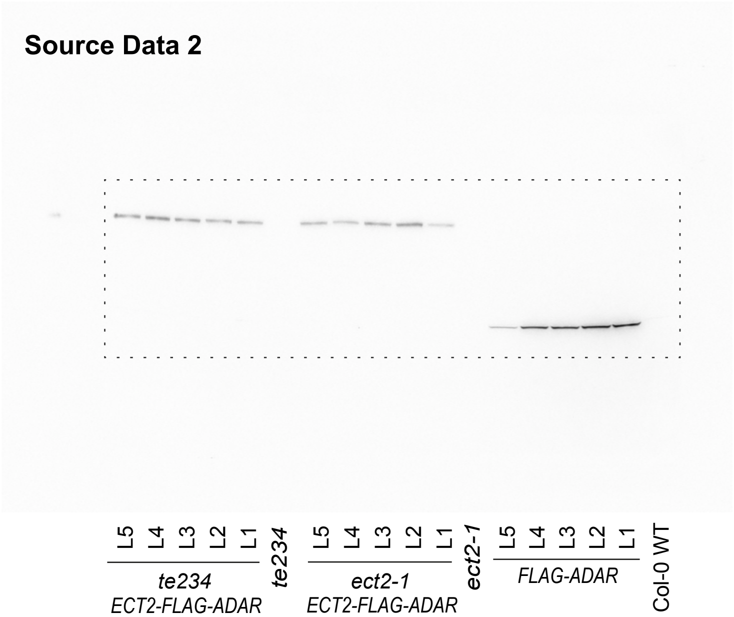

Bright field

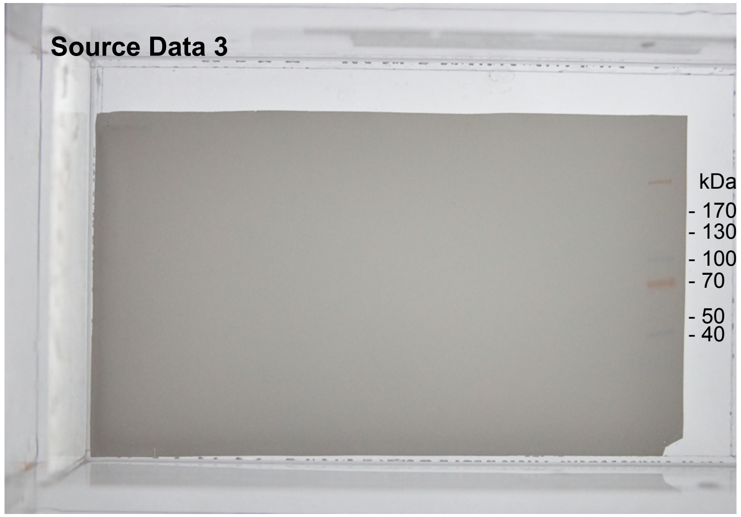

Amido black

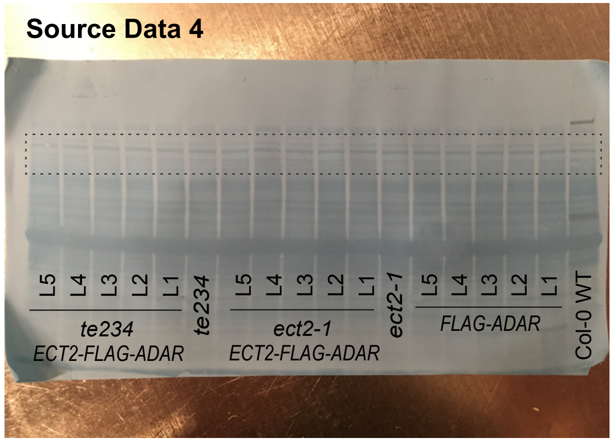

Supplement: Figure 1—figure supplement 1—source data 1. [file elife-72375-fig1-figsupp1-data1.zip › ECT2-Targeting_v2_Figure1-Figure_supplement1-Source_data1.pdf]
