# Supplementary figures and images for: Principles of mRNA targeting via the Arabidopsis m6A-binding protein ECT2
Source: eLife. 2021 Sep 30;10:e72375. doi: 10.7554/eLife.72375 (PMC8796052; doi:10.7554/eLife.72375)

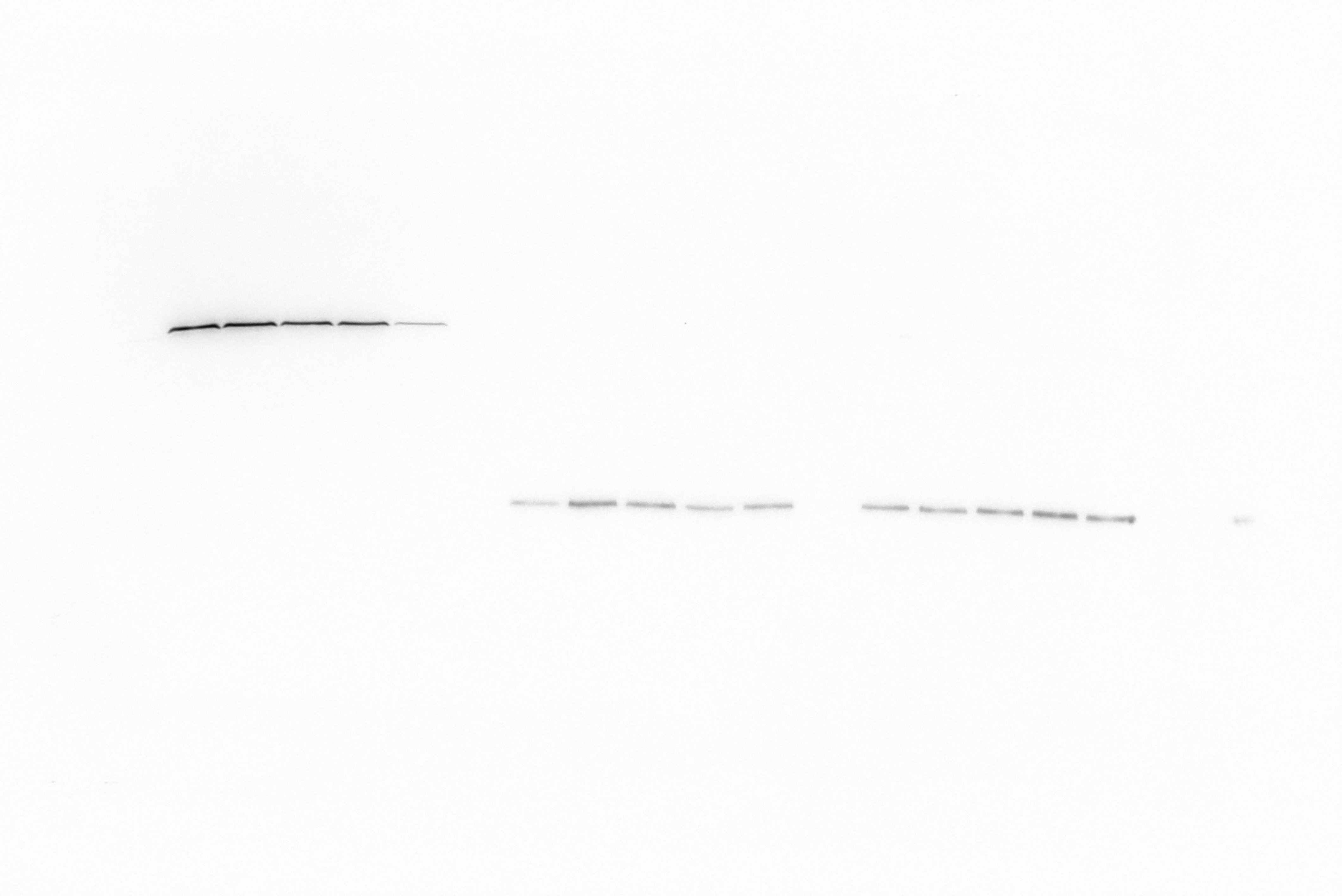

Supplement: Figure 1—figure supplement 1—source data 1. [file elife-72375-fig1-figsupp1-data1.zip › ECT2-Targeting_v2_Figure1-Figure_supplement1-Source_data2.tif]

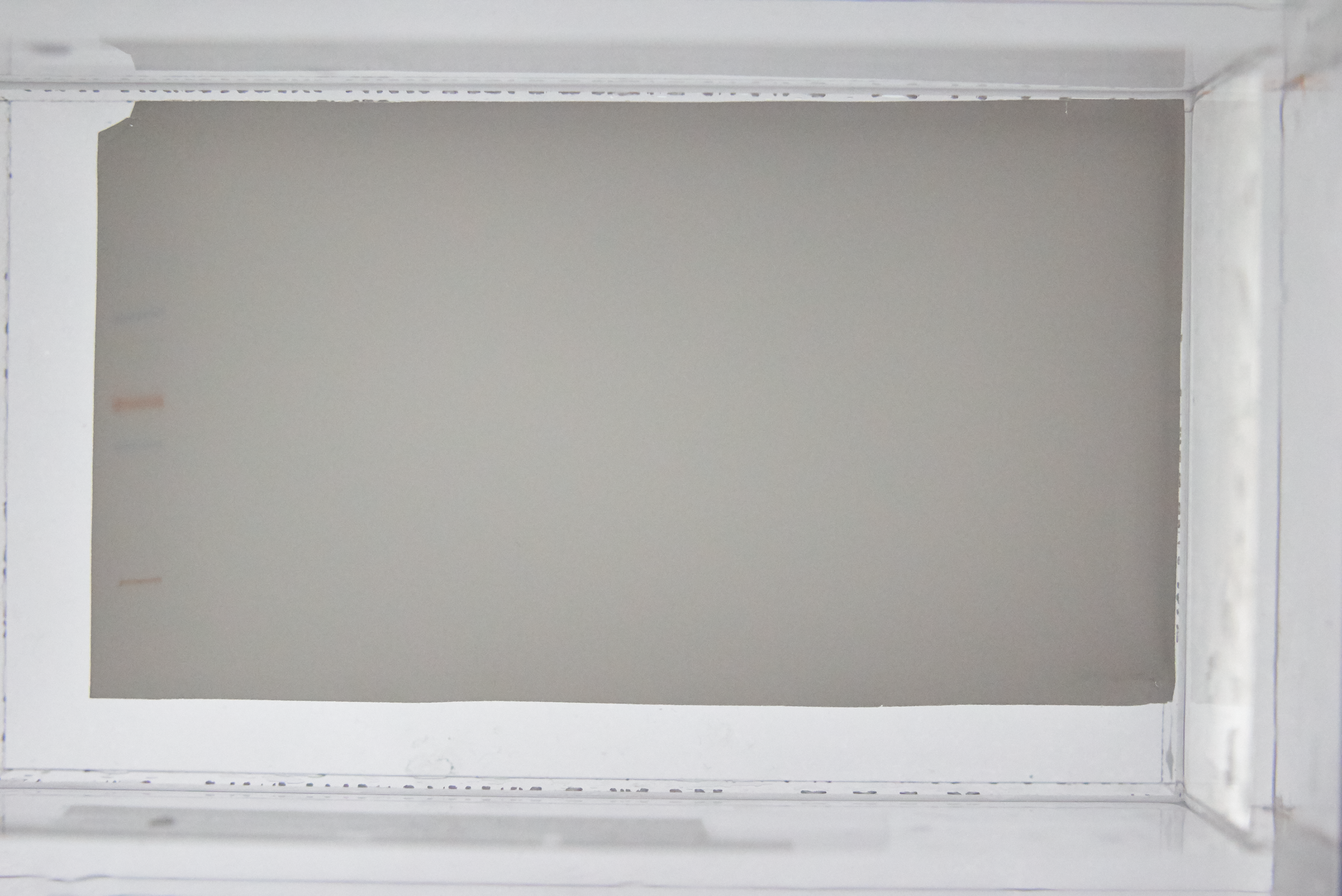

Supplement: Figure 1—figure supplement 1—source data 1. [file elife-72375-fig1-figsupp1-data1.zip › ECT2-Targeting_v2_Figure1-Figure_supplement1-Source_data3.tif]

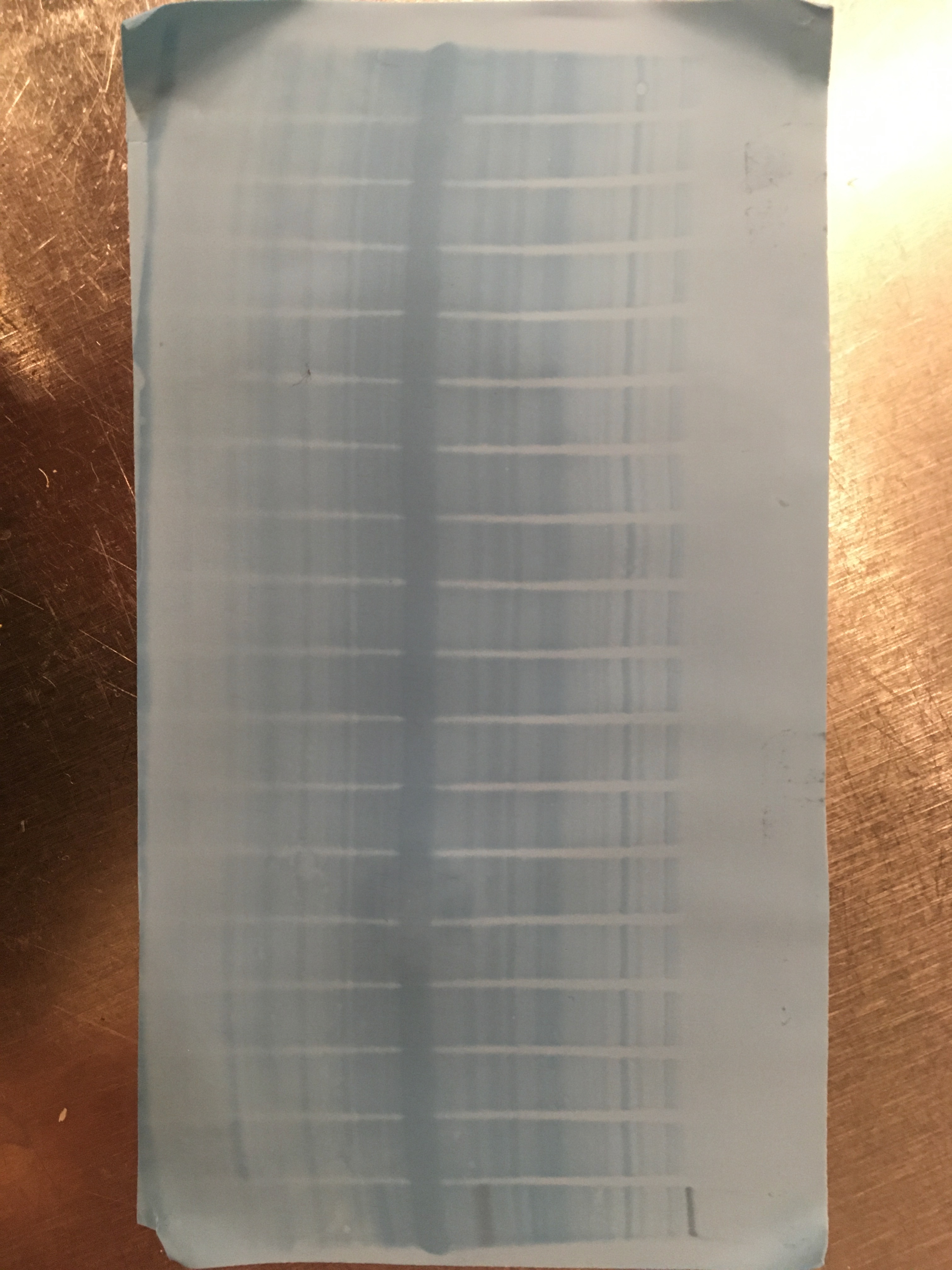

Supplement: Figure 1—figure supplement 1—source data 1. [file elife-72375-fig1-figsupp1-data1.zip › ECT2-Targeting_v2_Figure1-Figure_supplement1-Source_data4.jpeg]

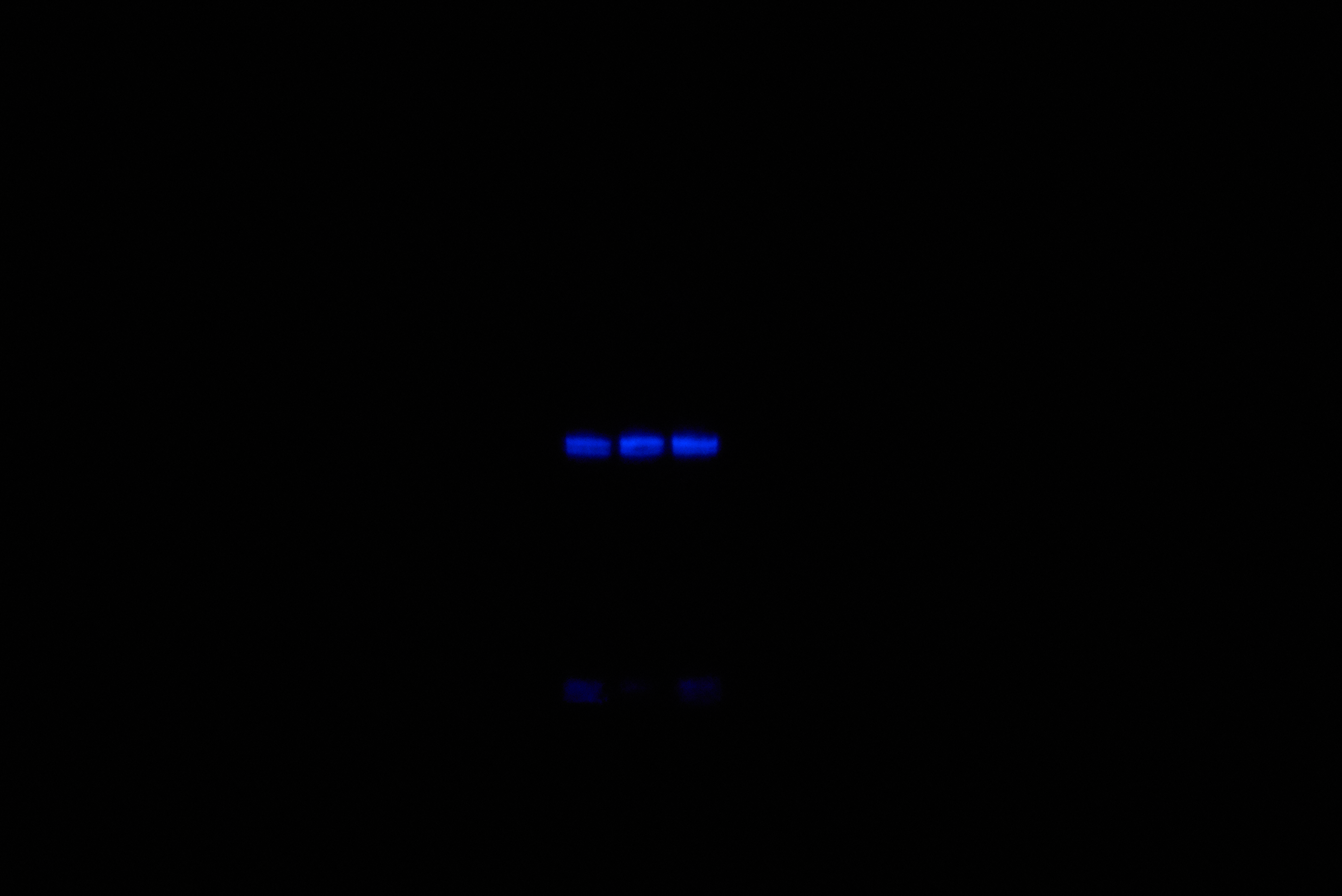

Supplement: Figure 3—source data 1. [file elife-72375-fig3-data1.zip › ECT2-Targeting_v2_Figure3-Source_data_10.tif]

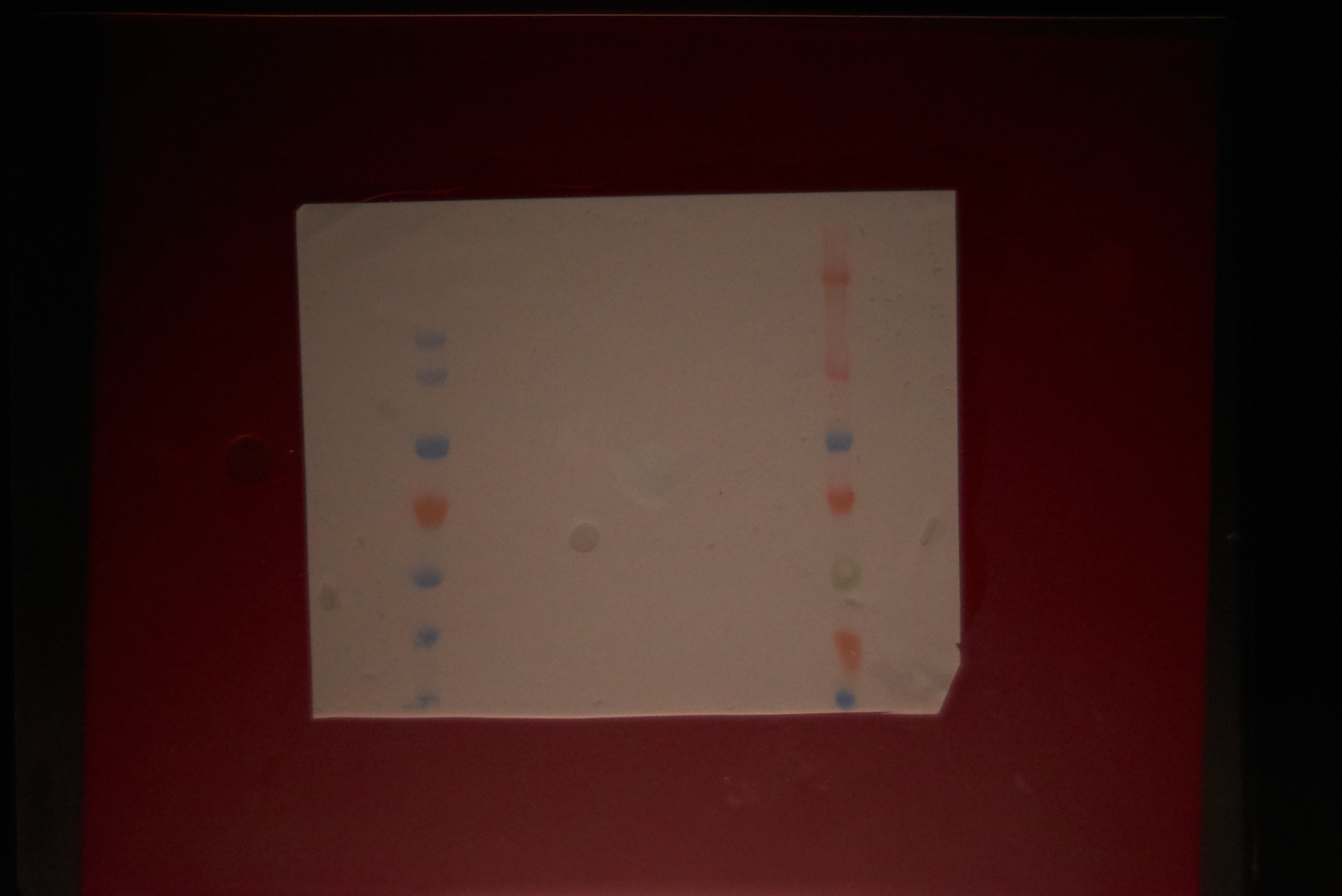

Supplement: Figure 3—source data 1. [file elife-72375-fig3-data1.zip › ECT2-Targeting_v2_Figure3-Source_data_11.tif]

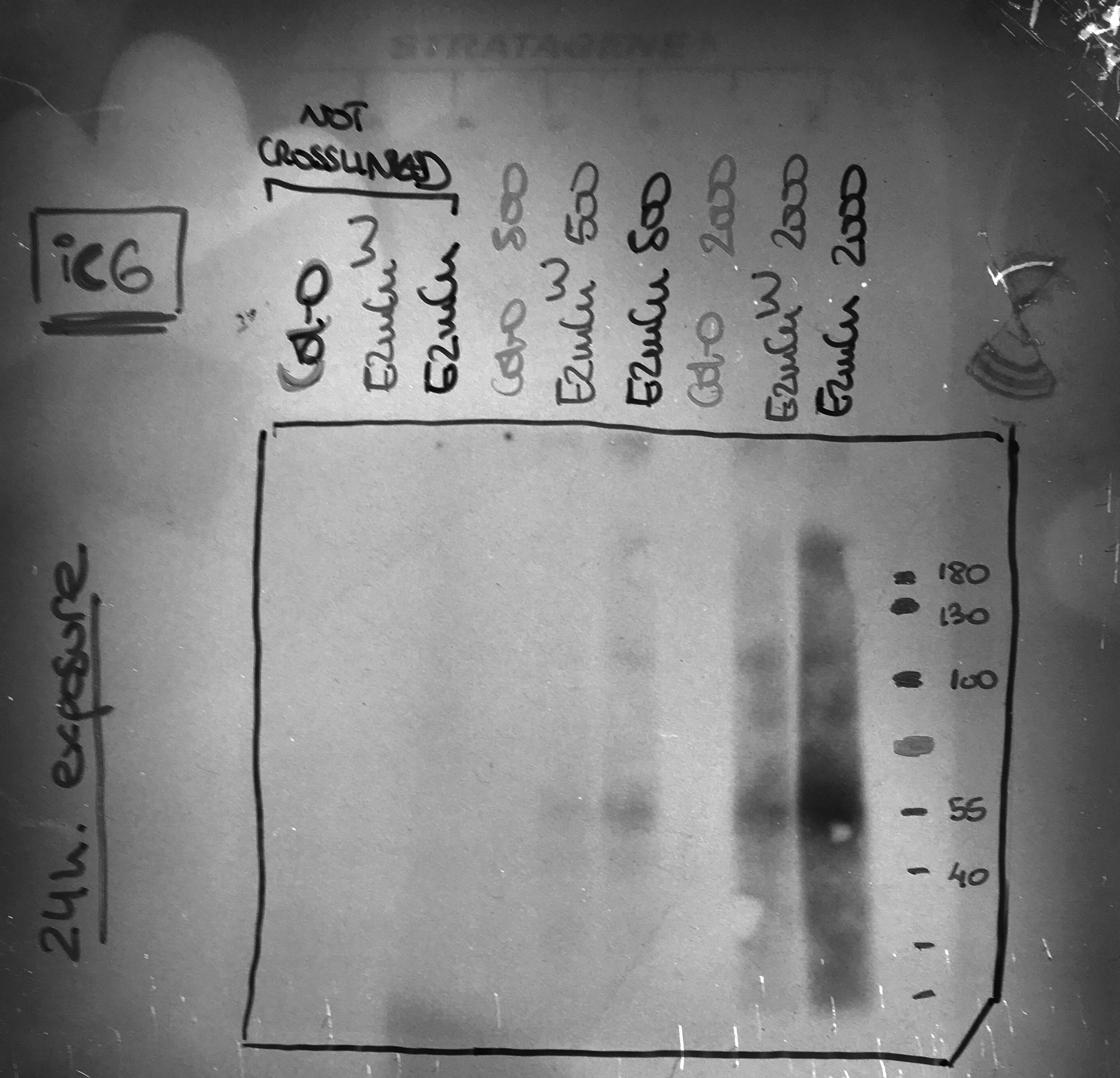

Supplement: Figure 3—source data 1. [file elife-72375-fig3-data1.zip › ECT2-Targeting_v2_Figure3-Source_data_2.jpg]

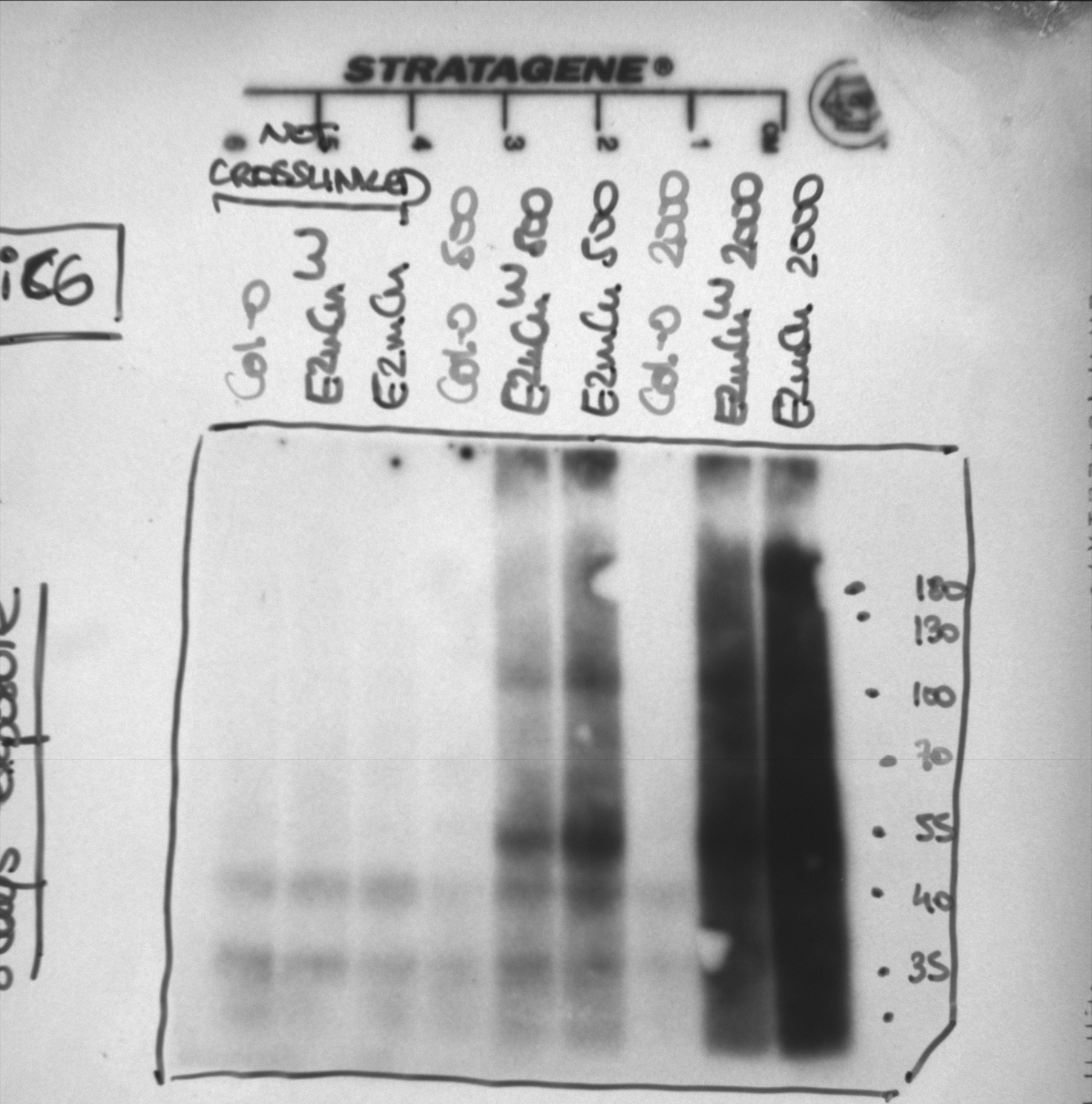

Supplement: Figure 3—source data 1. [file elife-72375-fig3-data1.zip › ECT2-Targeting_v2_Figure3-Source_data_3.jpg]

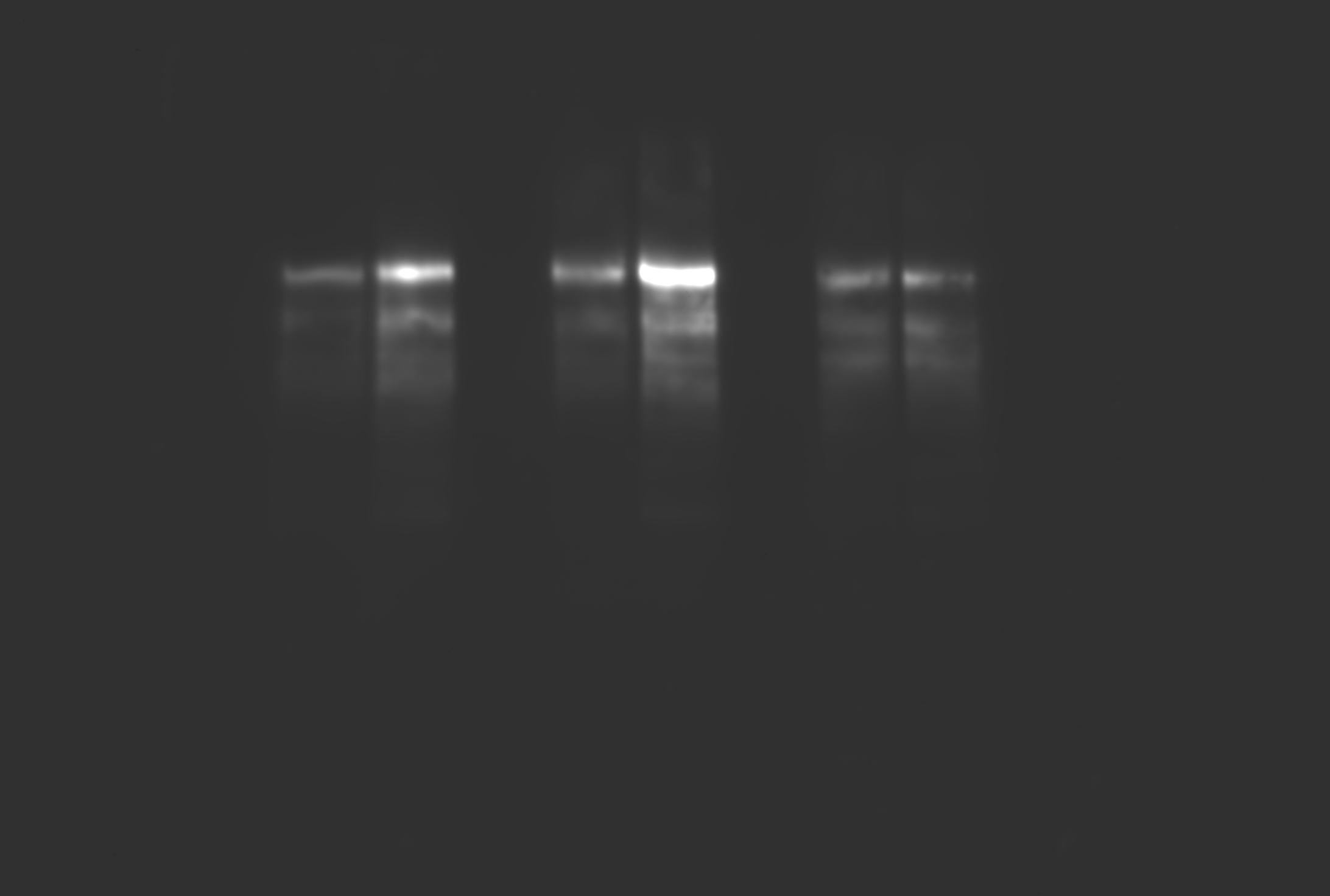

Supplement: Figure 3—source data 1. [file elife-72375-fig3-data1.zip › ECT2-Targeting_v2_Figure3-Source_data_4.tif]

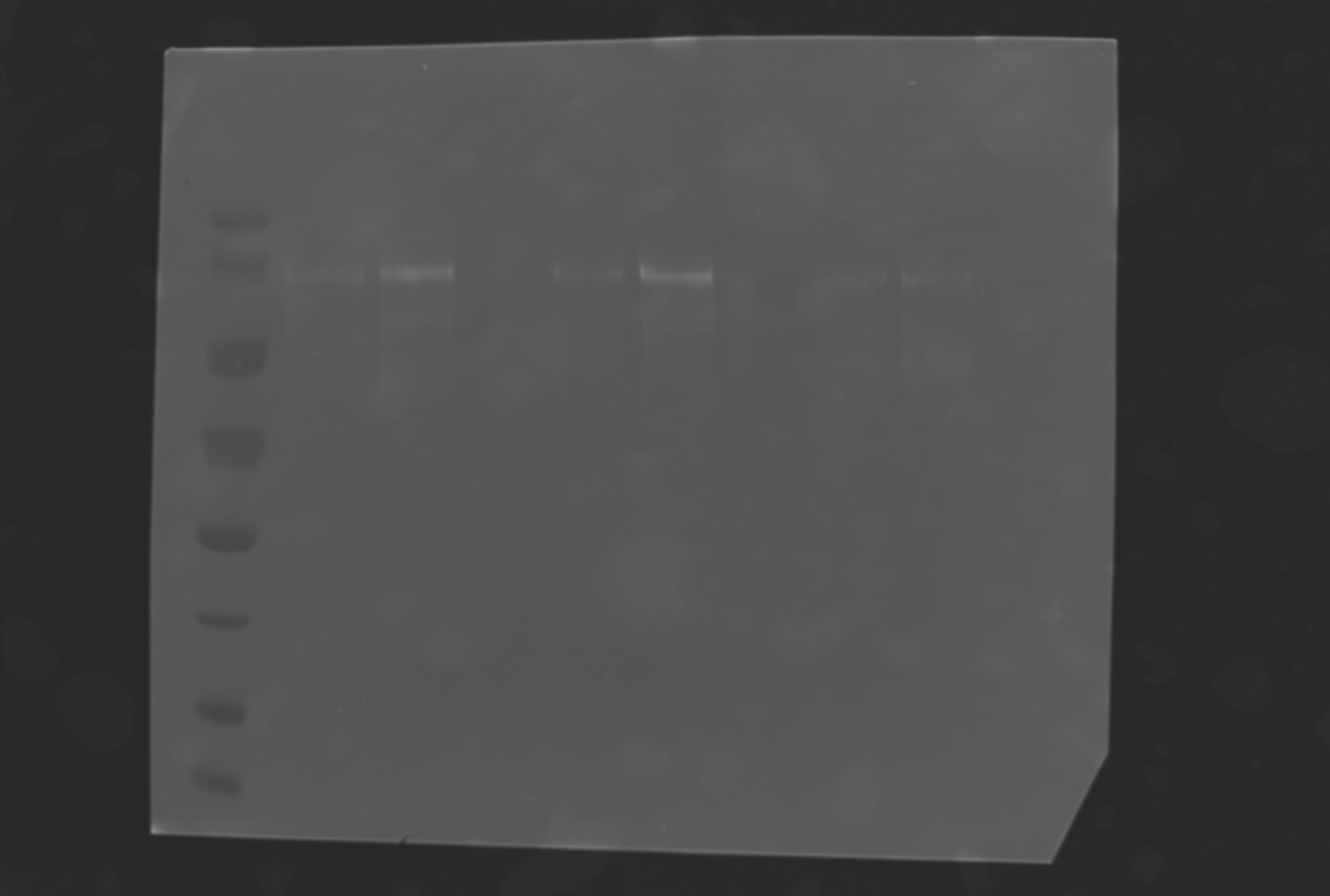

Supplement: Figure 3—source data 1. [file elife-72375-fig3-data1.zip › ECT2-Targeting_v2_Figure3-Source_data_5.tif]

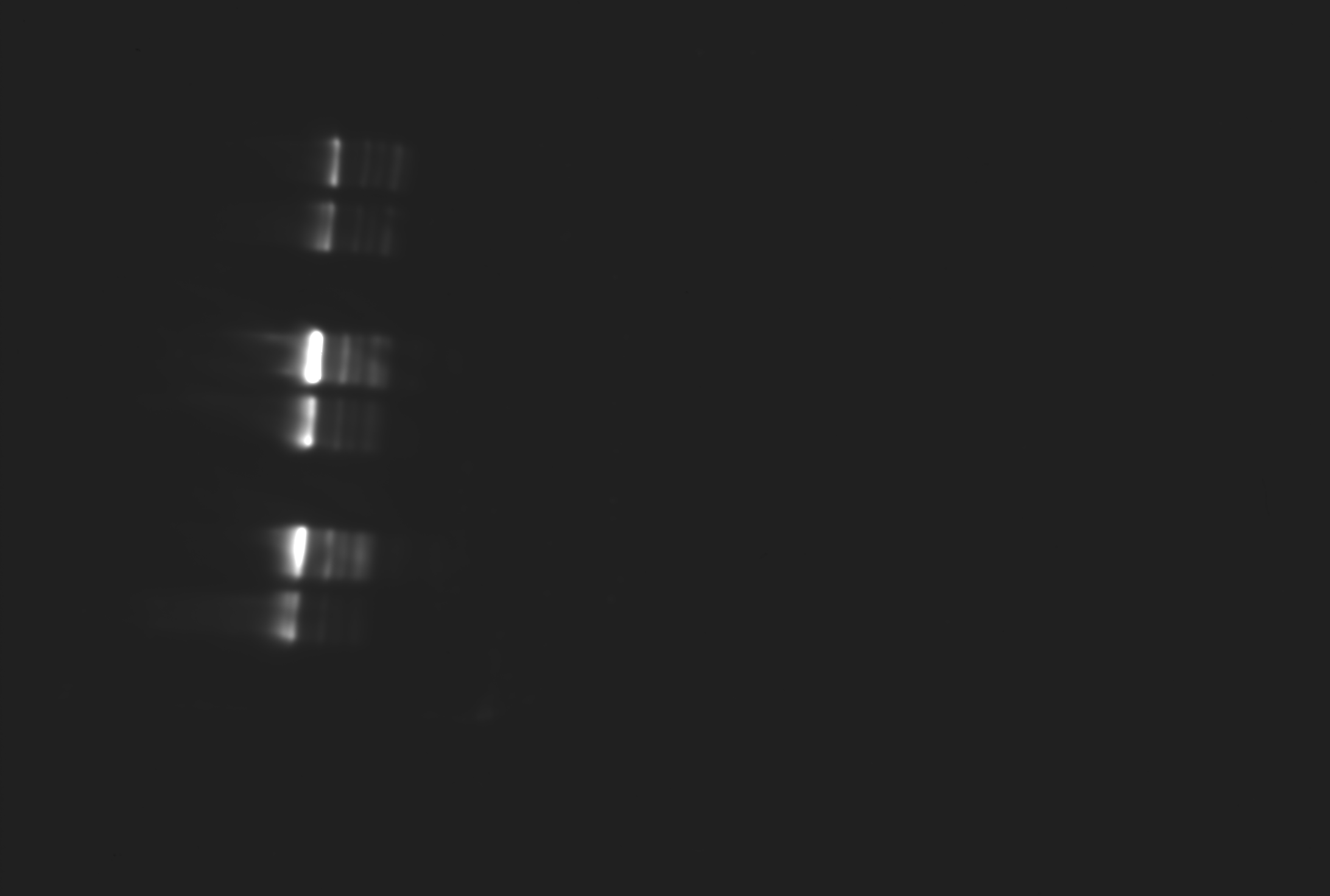

Supplement: Figure 3—source data 1. [file elife-72375-fig3-data1.zip › ECT2-Targeting_v2_Figure3-Source_data_6.tif]

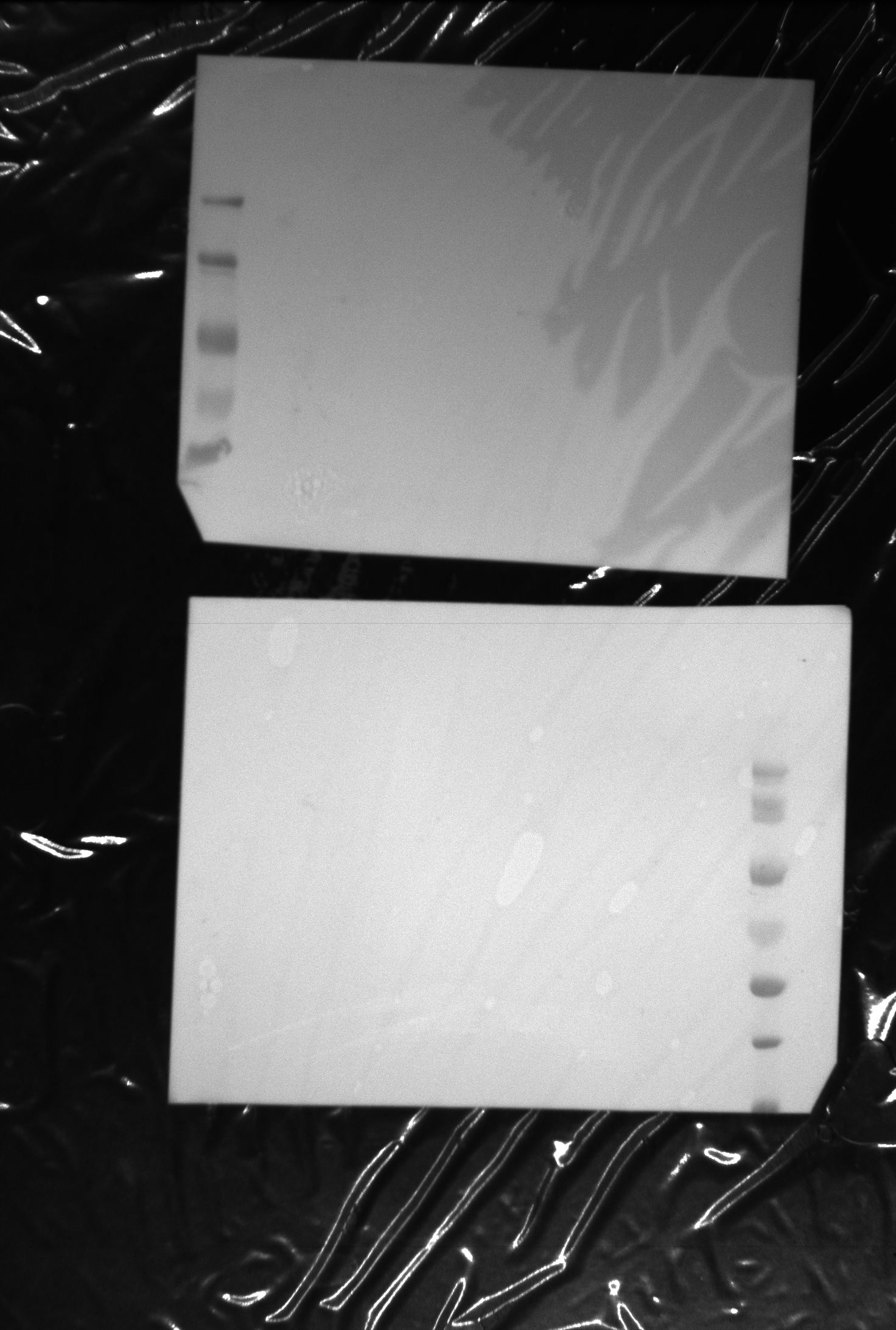

Supplement: Figure 3—source data 1. [file elife-72375-fig3-data1.zip › ECT2-Targeting_v2_Figure3-Source_data_7.tif]

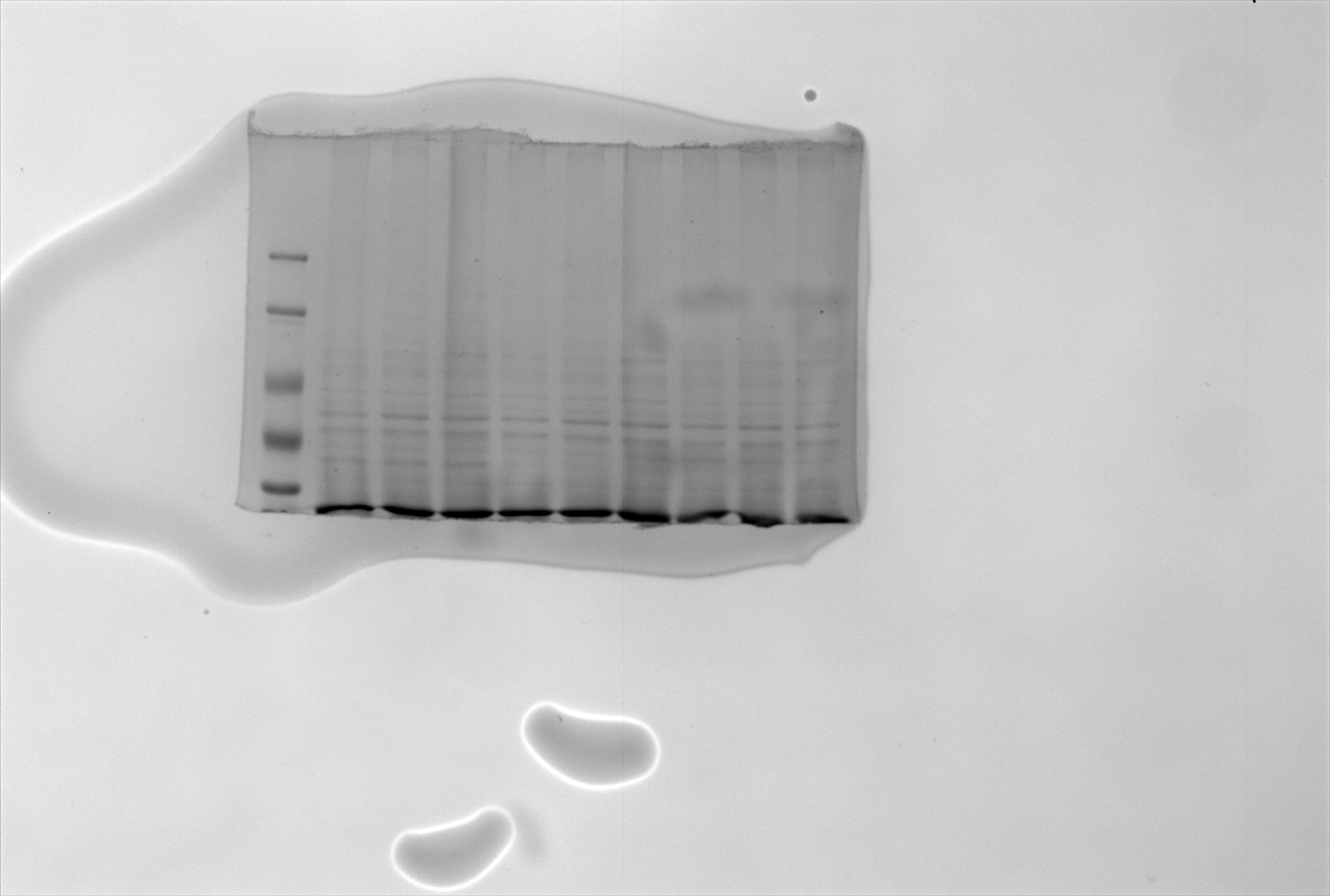

Supplement: Figure 3—source data 1. [file elife-72375-fig3-data1.zip › ECT2-Targeting_v2_Figure3-Source_data_8.tif]

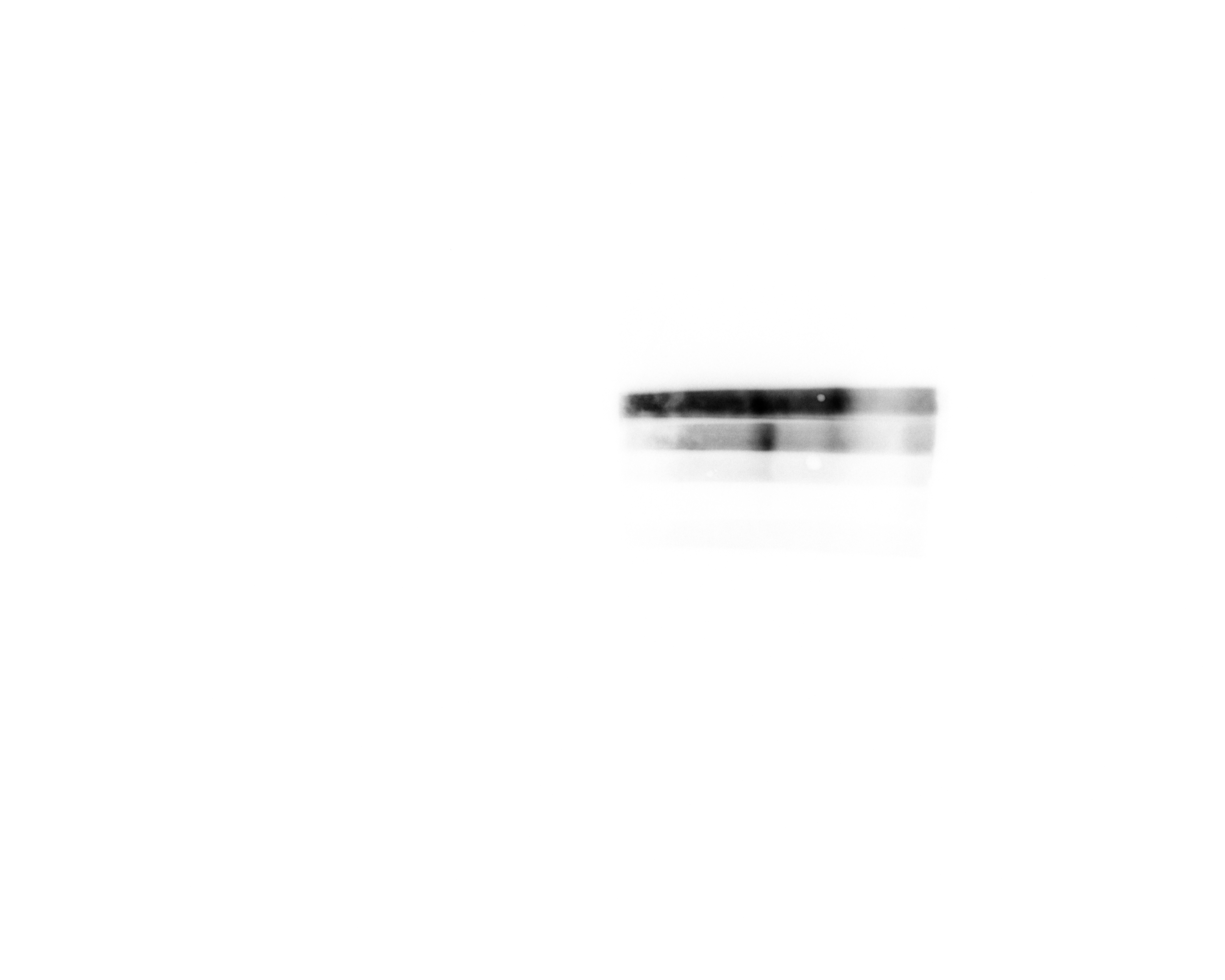

Supplement: Figure 3—source data 1. [file elife-72375-fig3-data1.zip › ECT2-Targeting_v2_Figure3-Source_data_9.tif]

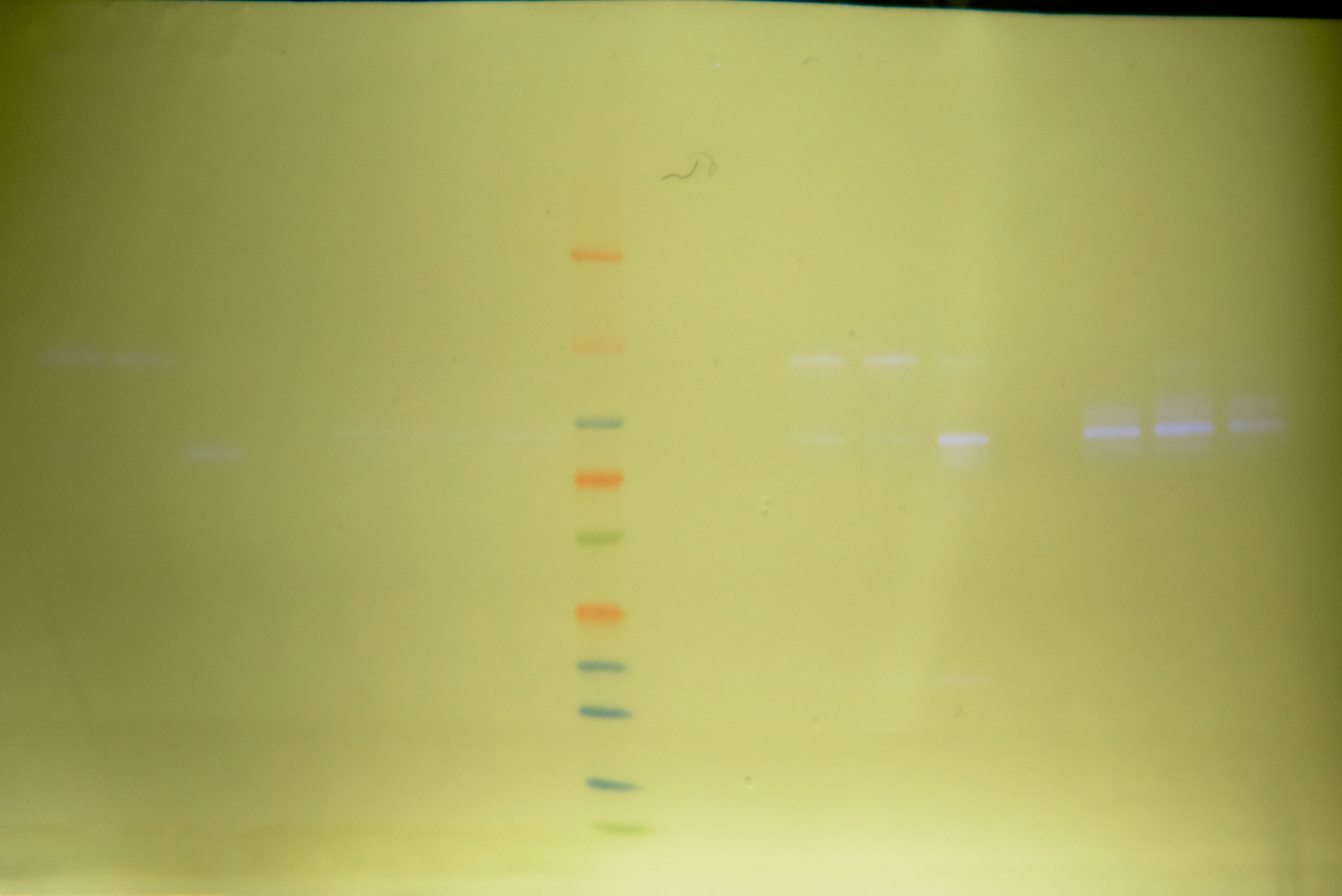

Supplement: Figure 3—figure supplement 1—source data 1. [file elife-72375-fig3-figsupp1-data1.zip › ECT2-Targeting_v2_Figure3-Figure_supplement1-Source_data10.jpg]

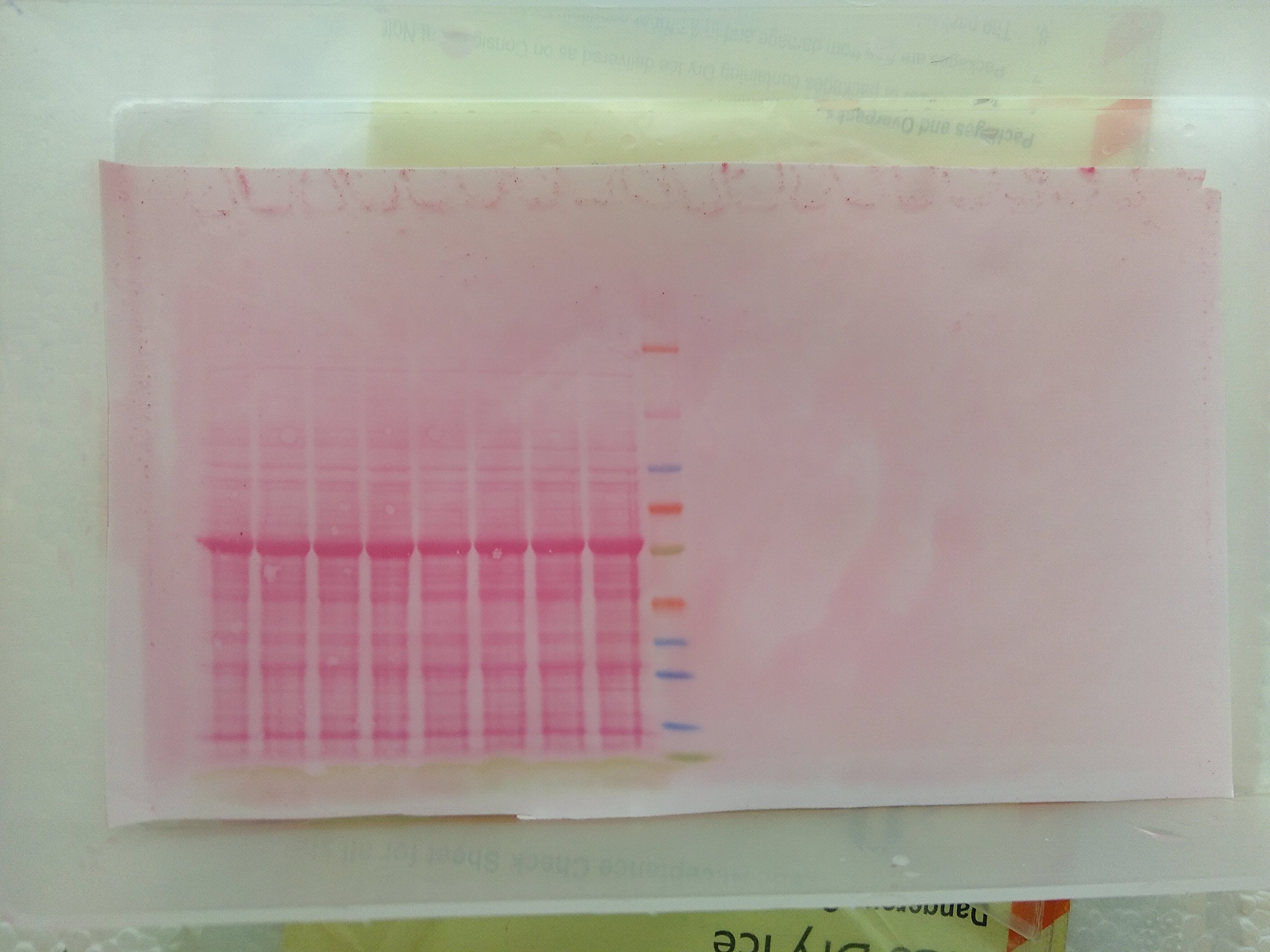

Supplement: Figure 3—figure supplement 1—source data 1. [file elife-72375-fig3-figsupp1-data1.zip › ECT2-Targeting_v2_Figure3-Figure_supplement1-Source_data11.jpg]

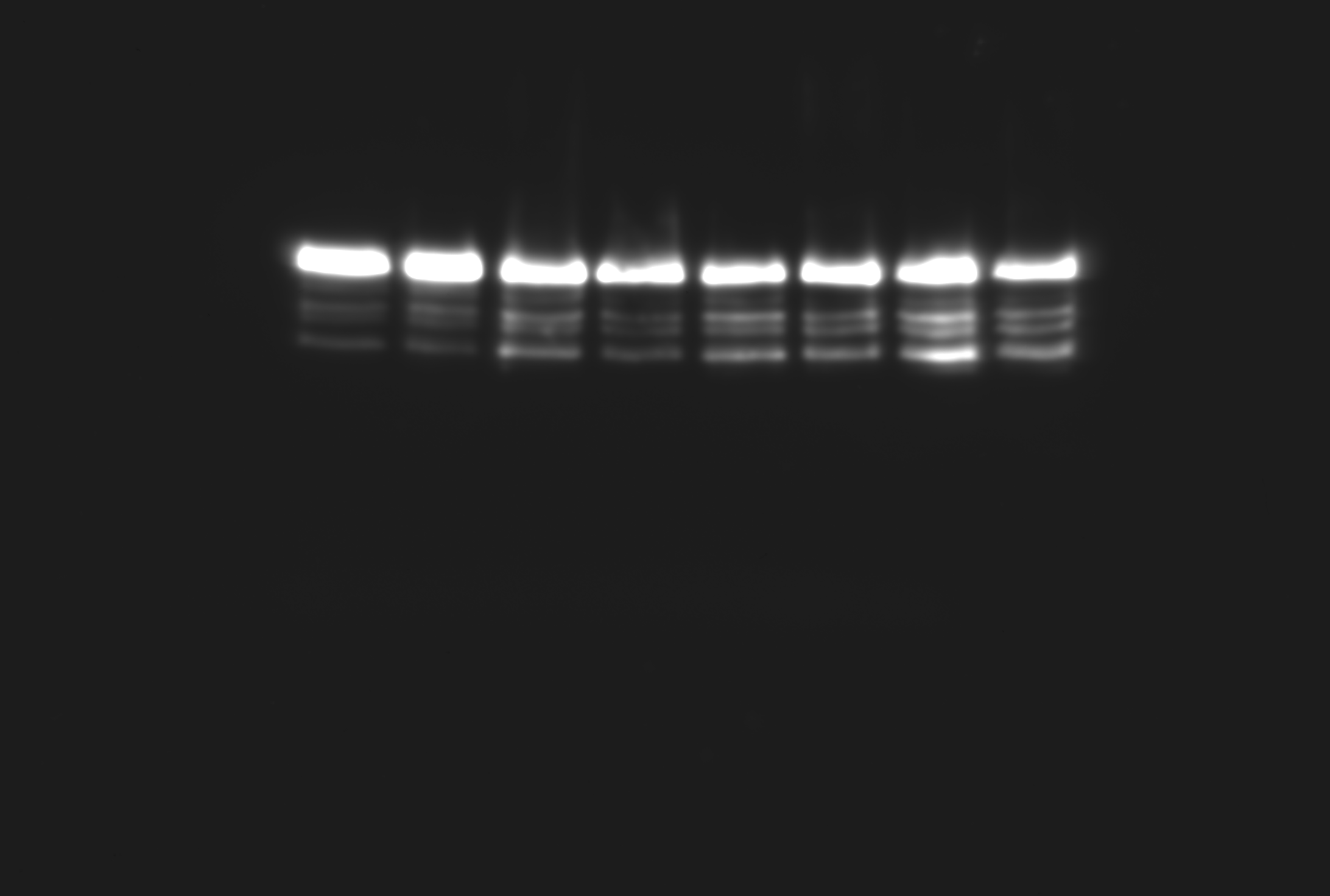

Supplement: Figure 3—figure supplement 1—source data 1. [file elife-72375-fig3-figsupp1-data1.zip › ECT2-Targeting_v2_Figure3-Figure_supplement1-Source_data12.tif]

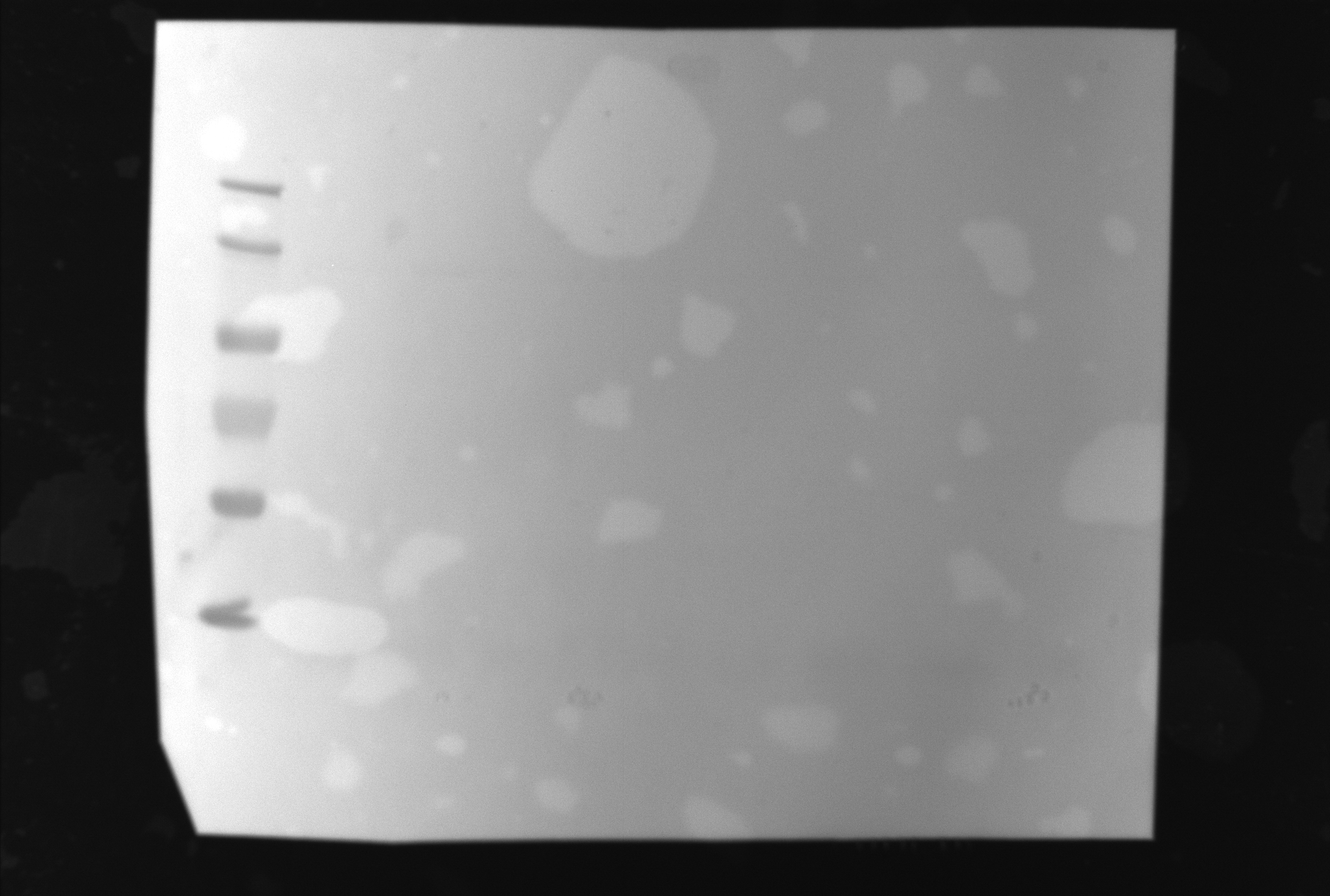

Supplement: Figure 3—figure supplement 1—source data 1. [file elife-72375-fig3-figsupp1-data1.zip › ECT2-Targeting_v2_Figure3-Figure_supplement1-Source_data13.tif]

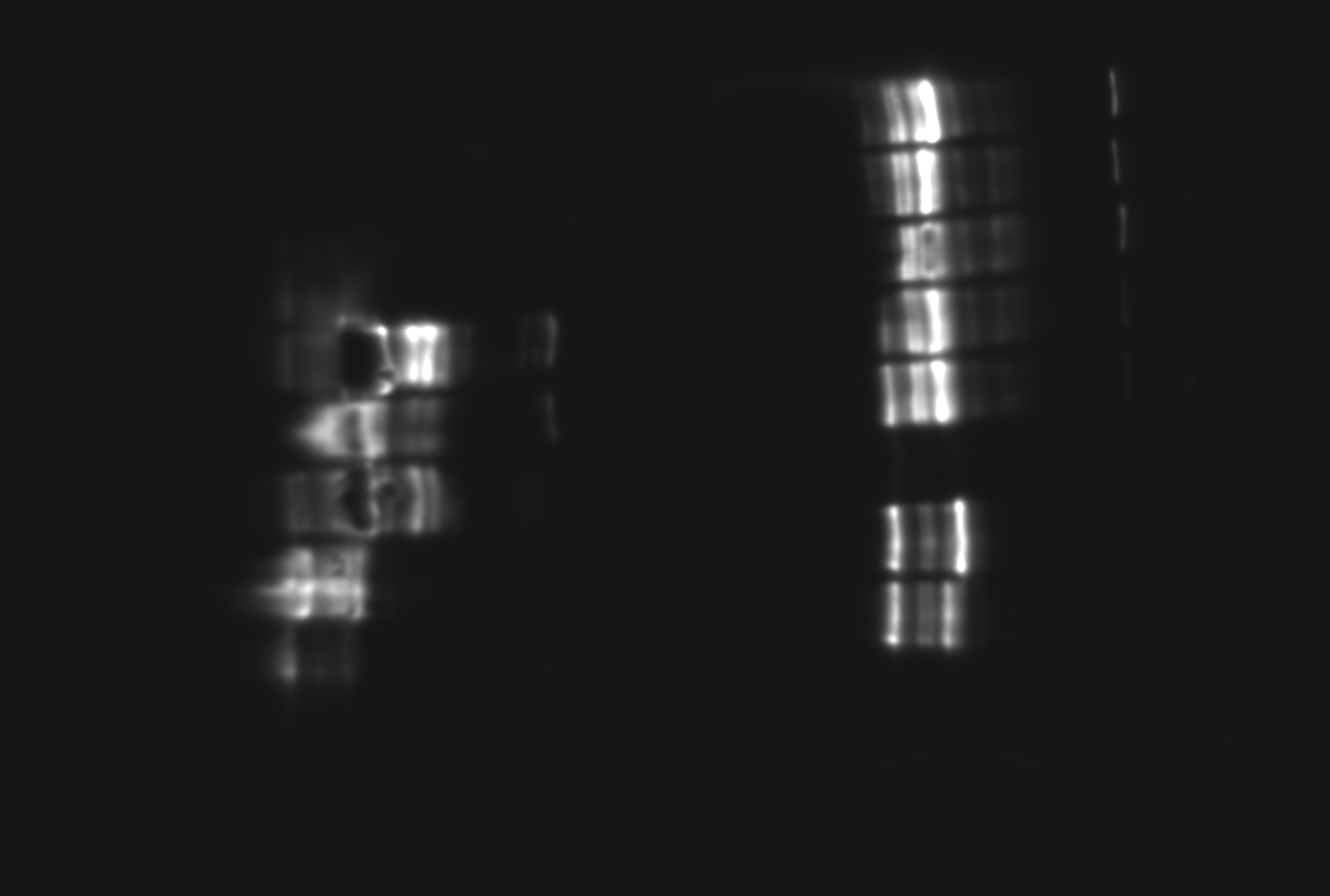

Supplement: Figure 3—figure supplement 1—source data 1. [file elife-72375-fig3-figsupp1-data1.zip › ECT2-Targeting_v2_Figure3-Figure_supplement1-Source_data14.tif]

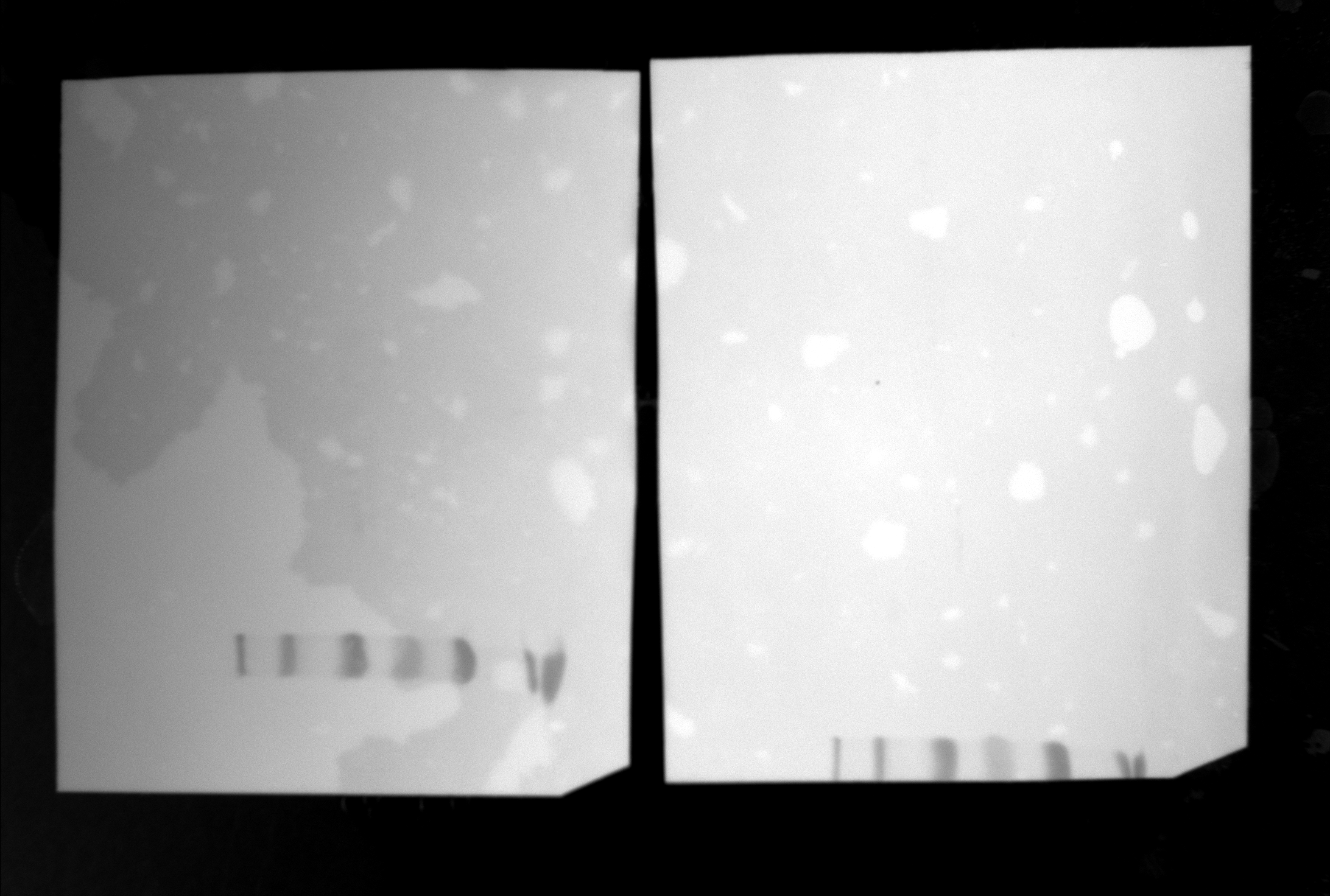

Supplement: Figure 3—figure supplement 1—source data 1. [file elife-72375-fig3-figsupp1-data1.zip › ECT2-Targeting_v2_Figure3-Figure_supplement1-Source_data15.tif]

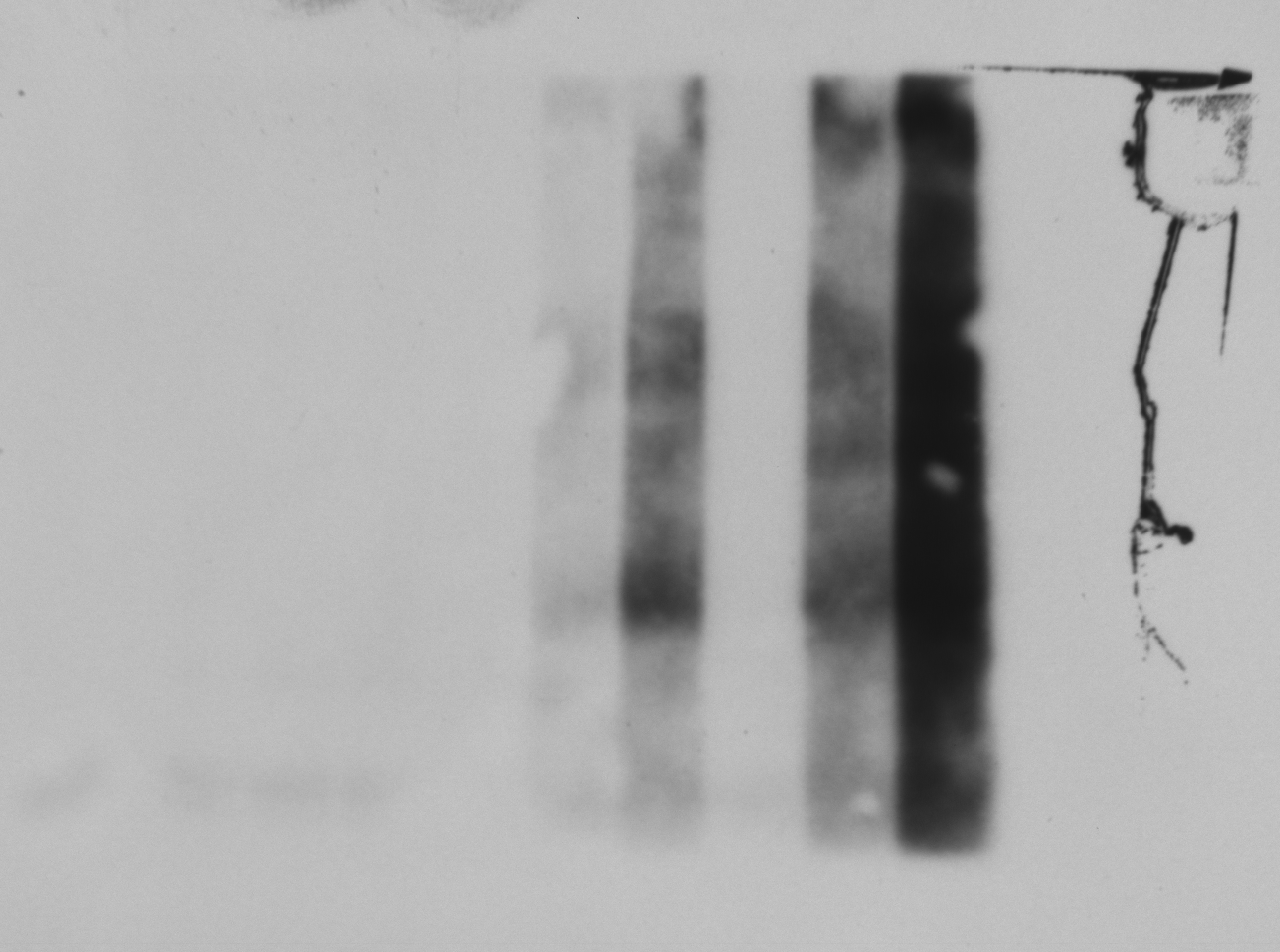

Supplement: Figure 3—figure supplement 1—source data 1. [file elife-72375-fig3-figsupp1-data1.zip › ECT2-Targeting_v2_Figure3-Figure_supplement1-Source_data2.tif]

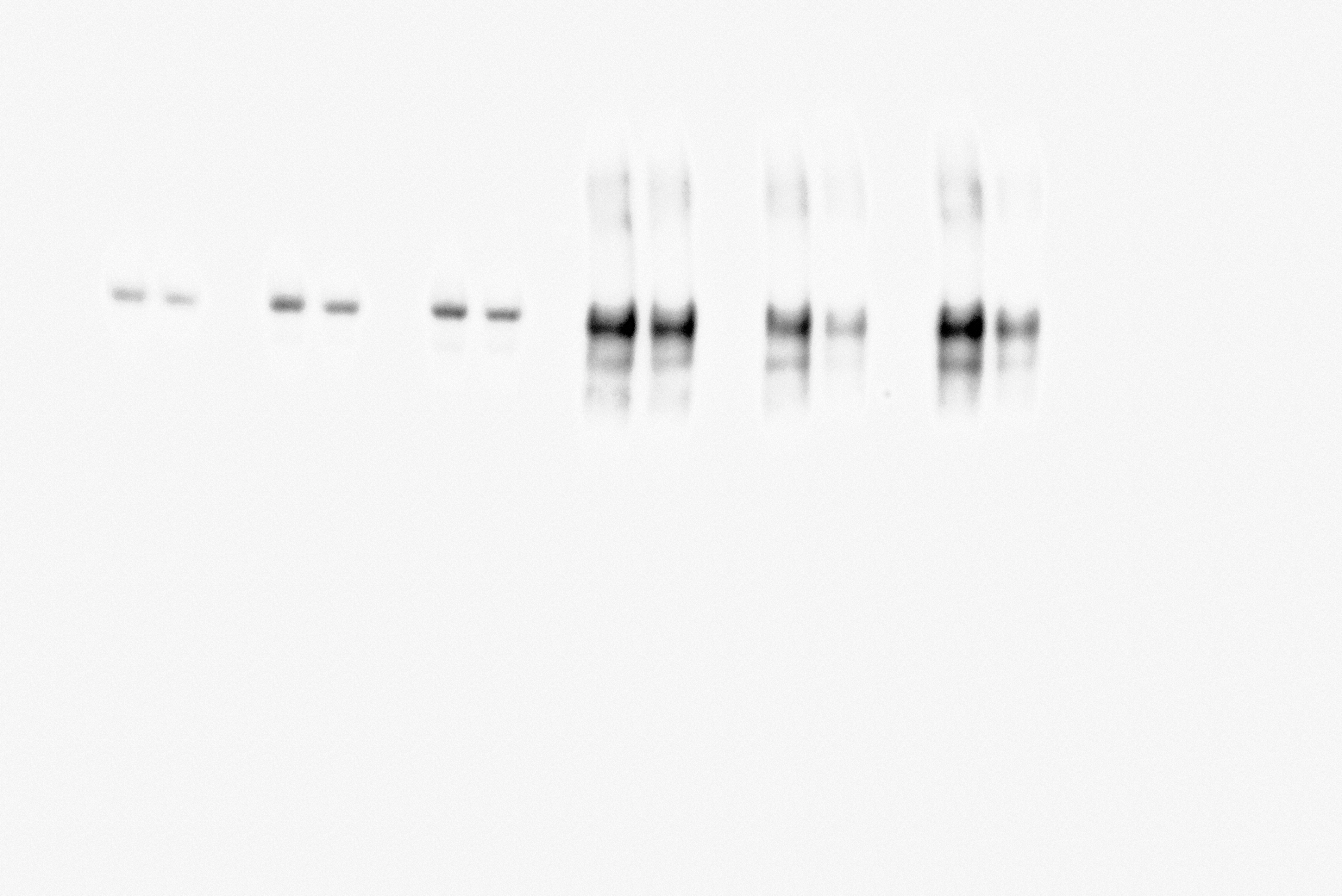

Supplement: Figure 3—figure supplement 1—source data 1. [file elife-72375-fig3-figsupp1-data1.zip › ECT2-Targeting_v2_Figure3-Figure_supplement1-Source_data3.tif]

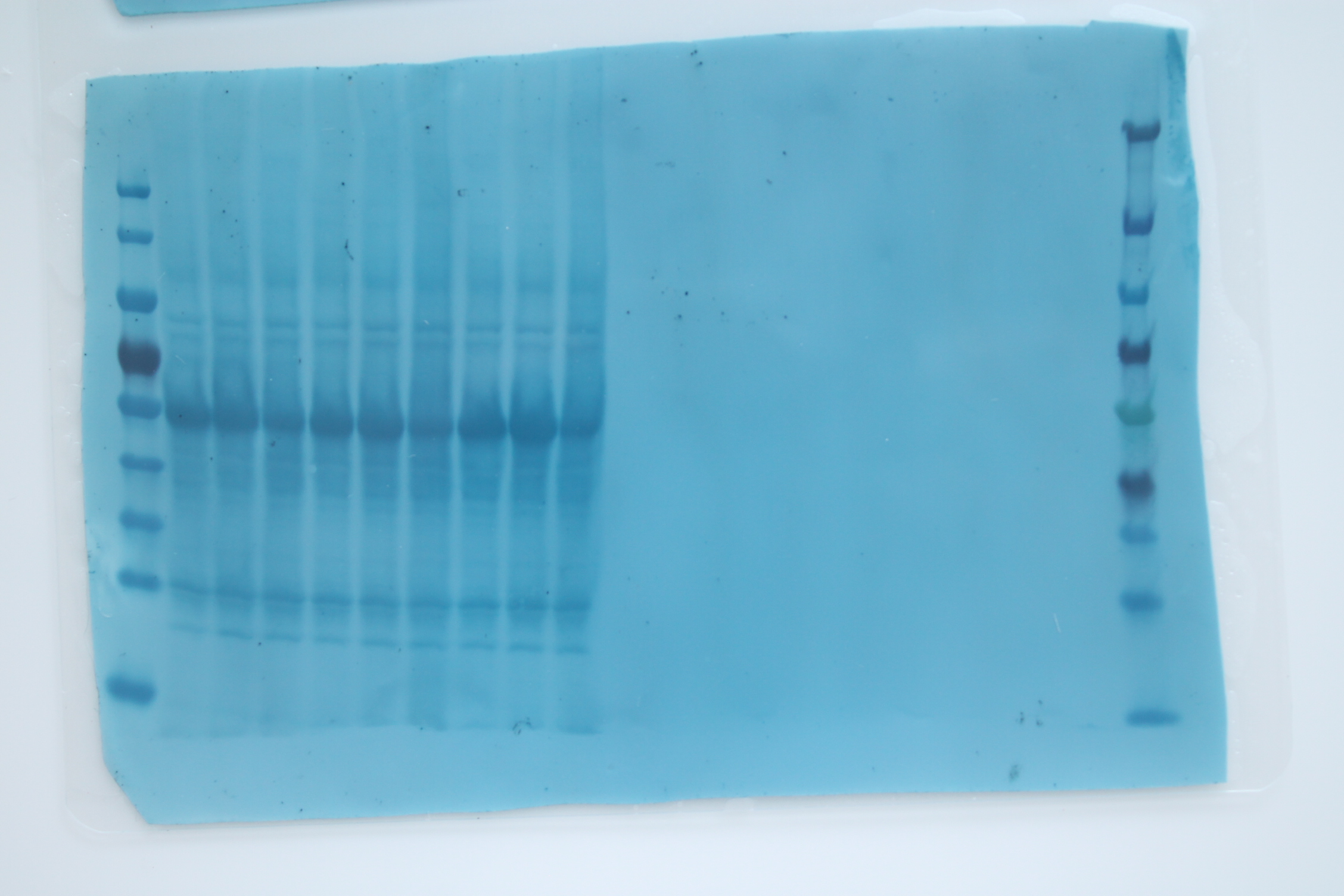

Supplement: Figure 3—figure supplement 1—source data 1. [file elife-72375-fig3-figsupp1-data1.zip › ECT2-Targeting_v2_Figure3-Figure_supplement1-Source_data4.JPG]

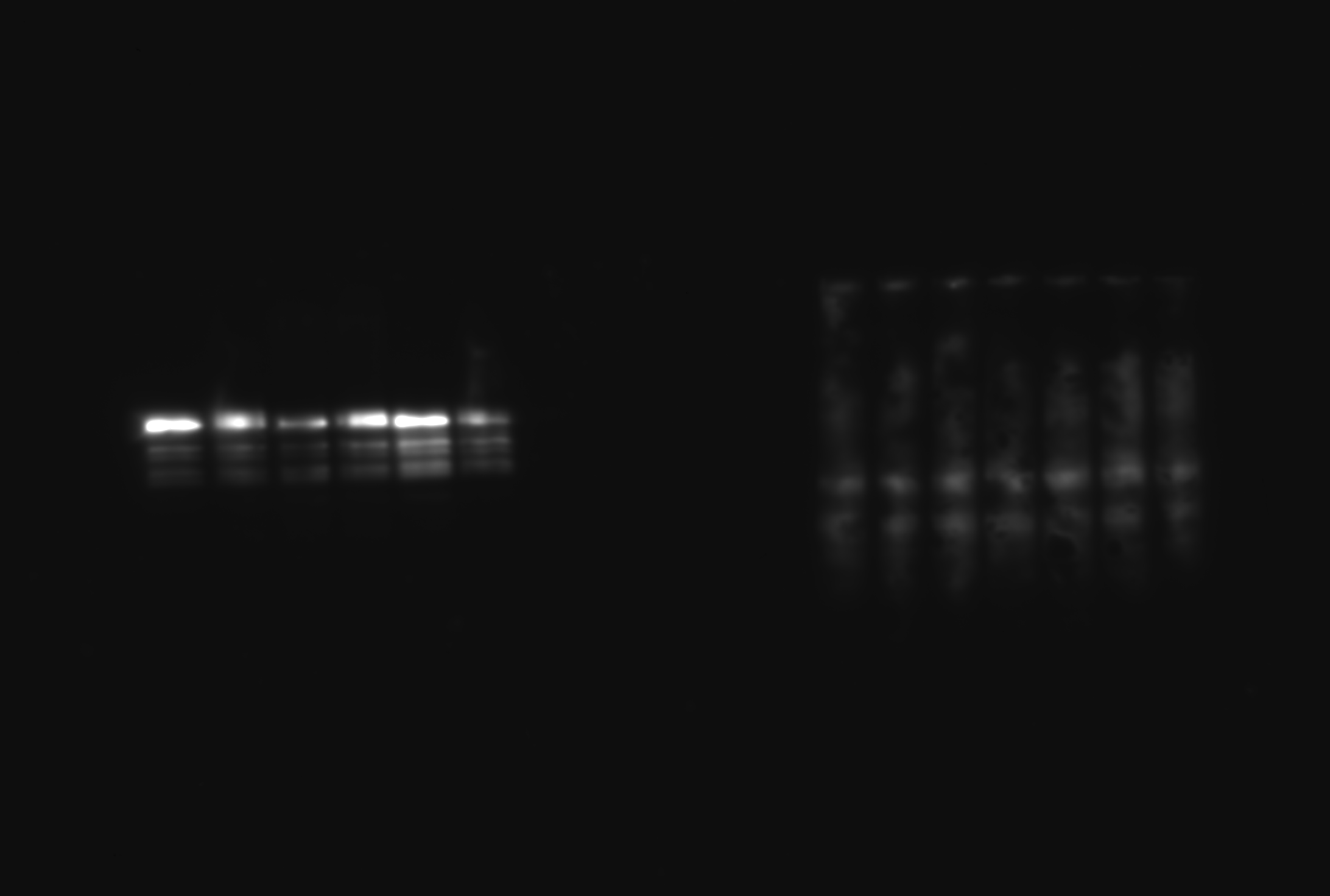

Supplement: Figure 3—figure supplement 1—source data 1. [file elife-72375-fig3-figsupp1-data1.zip › ECT2-Targeting_v2_Figure3-Figure_supplement1-Source_data5.tif]

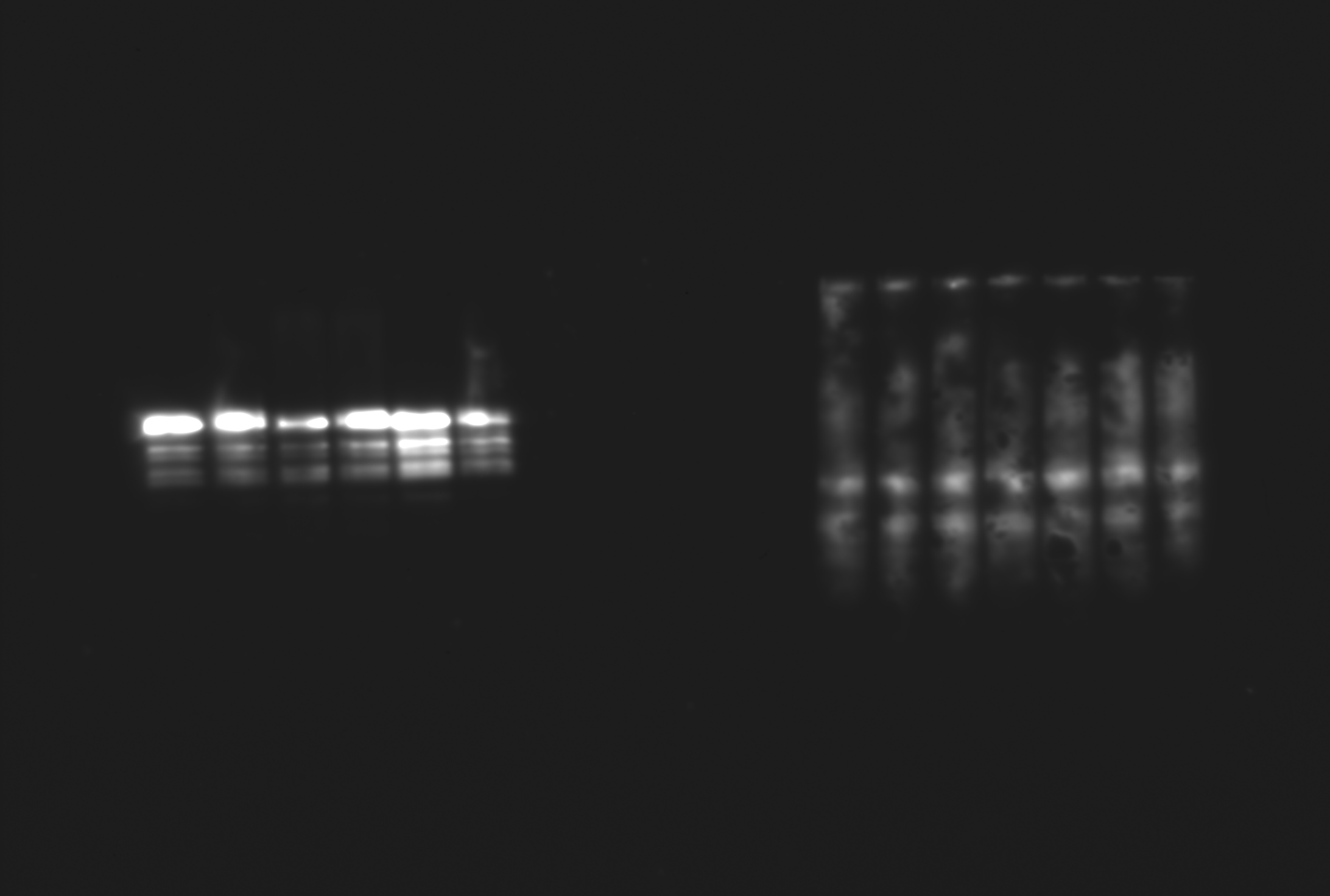

Supplement: Figure 3—figure supplement 1—source data 1. [file elife-72375-fig3-figsupp1-data1.zip › ECT2-Targeting_v2_Figure3-Figure_supplement1-Source_data6.tif]

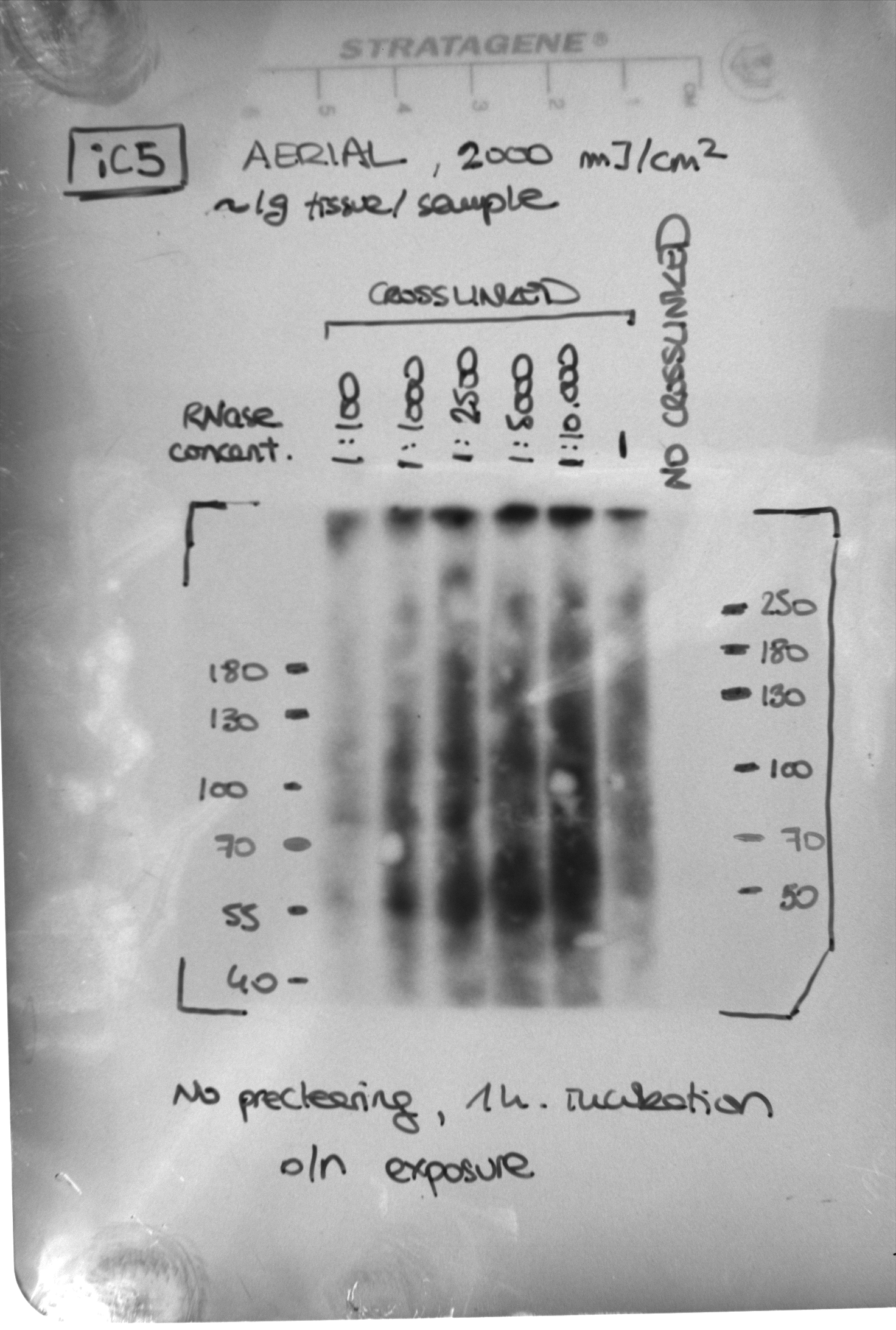

Supplement: Figure 3—figure supplement 1—source data 1. [file elife-72375-fig3-figsupp1-data1.zip › ECT2-Targeting_v2_Figure3-Figure_supplement1-Source_data7.tif]

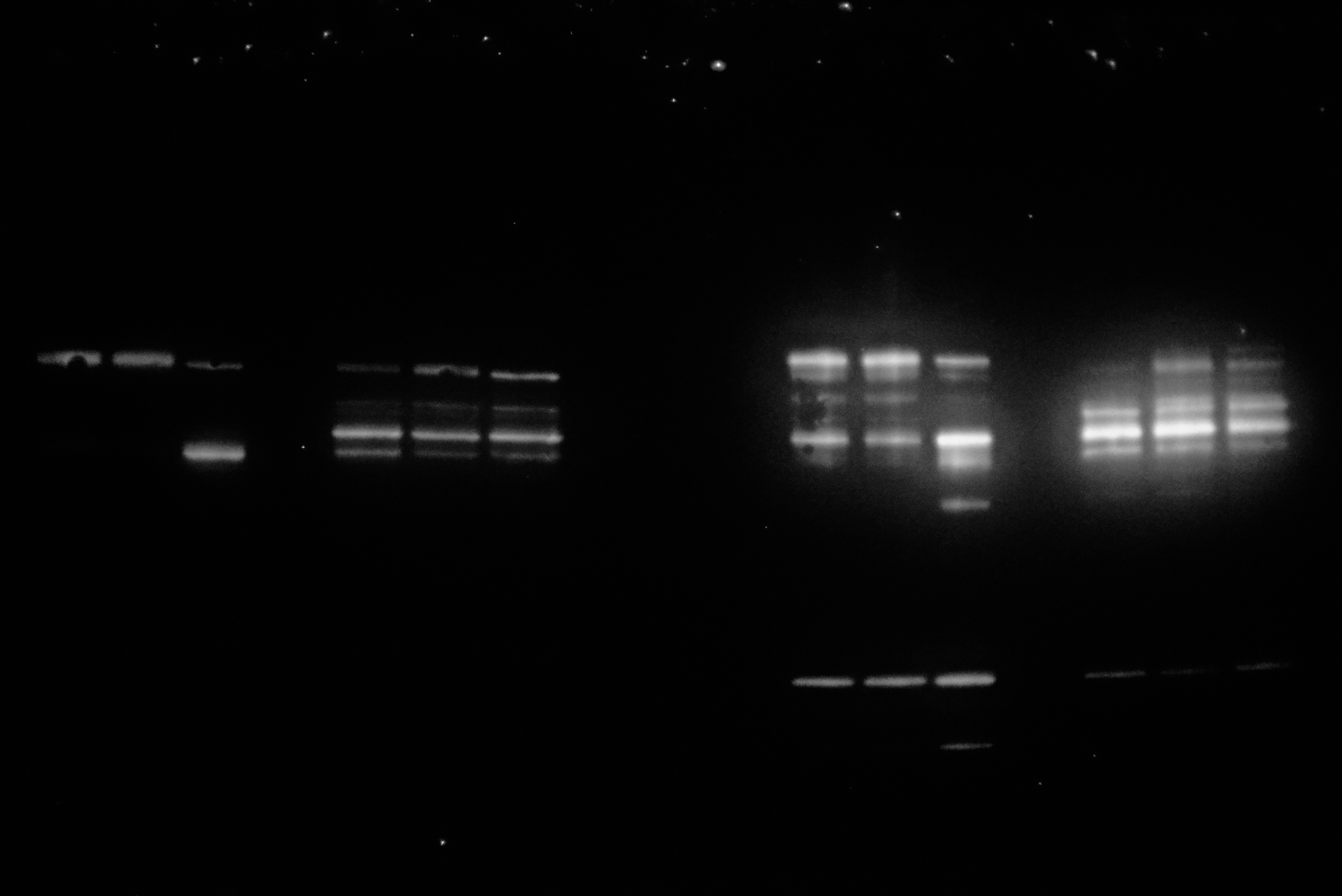

Supplement: Figure 3—figure supplement 1—source data 1. [file elife-72375-fig3-figsupp1-data1.zip › ECT2-Targeting_v2_Figure3-Figure_supplement1-Source_data8.jpg]

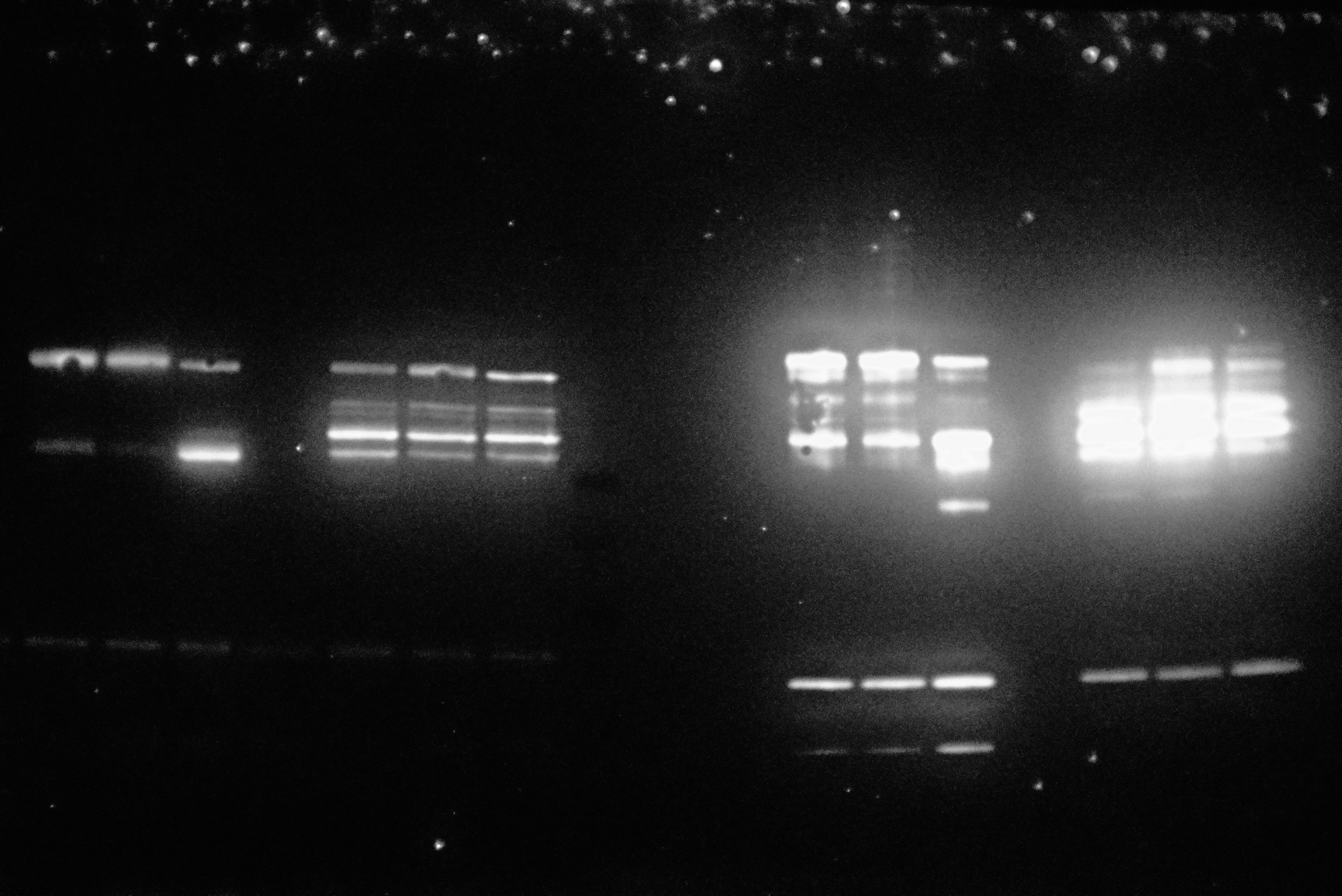

Supplement: Figure 3—figure supplement 1—source data 1. [file elife-72375-fig3-figsupp1-data1.zip › ECT2-Targeting_v2_Figure3-Figure_supplement1-Source_data9.jpg]

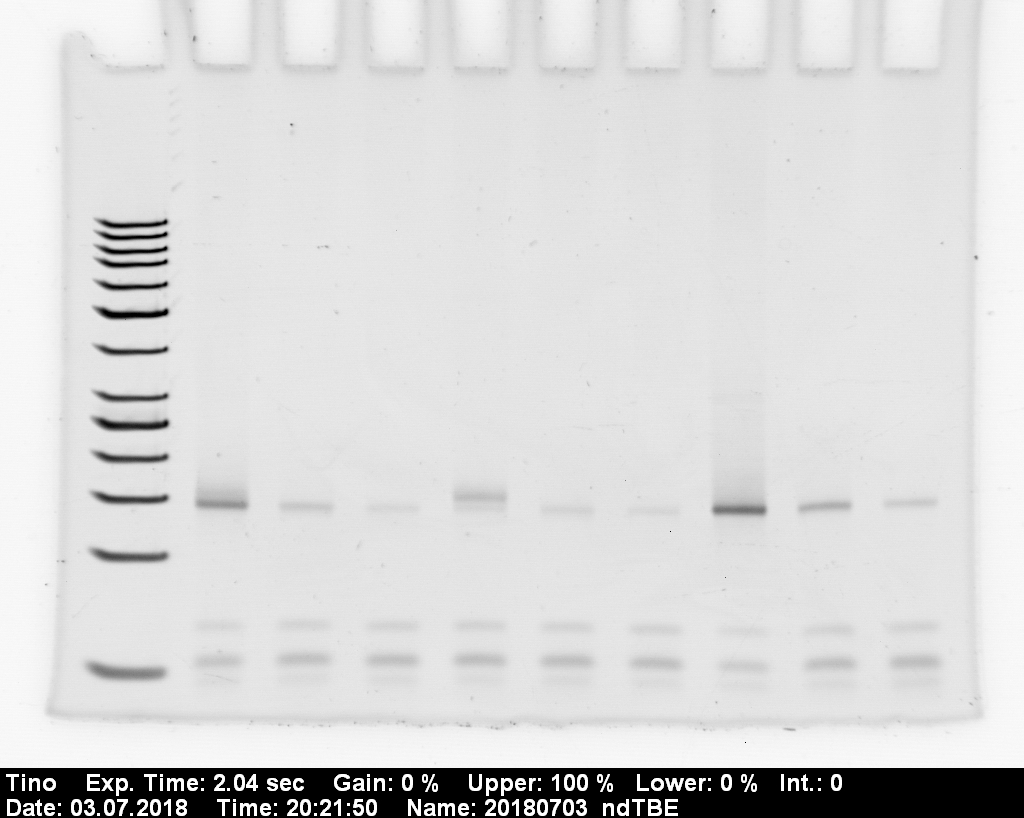

Supplement: Figure 3—figure supplement 3—source data 1. [file elife-72375-fig3-figsupp3-data1.zip › ECT2-Targeting_v2_Figure3-Figure_supplement3-Source_data10.jpg]

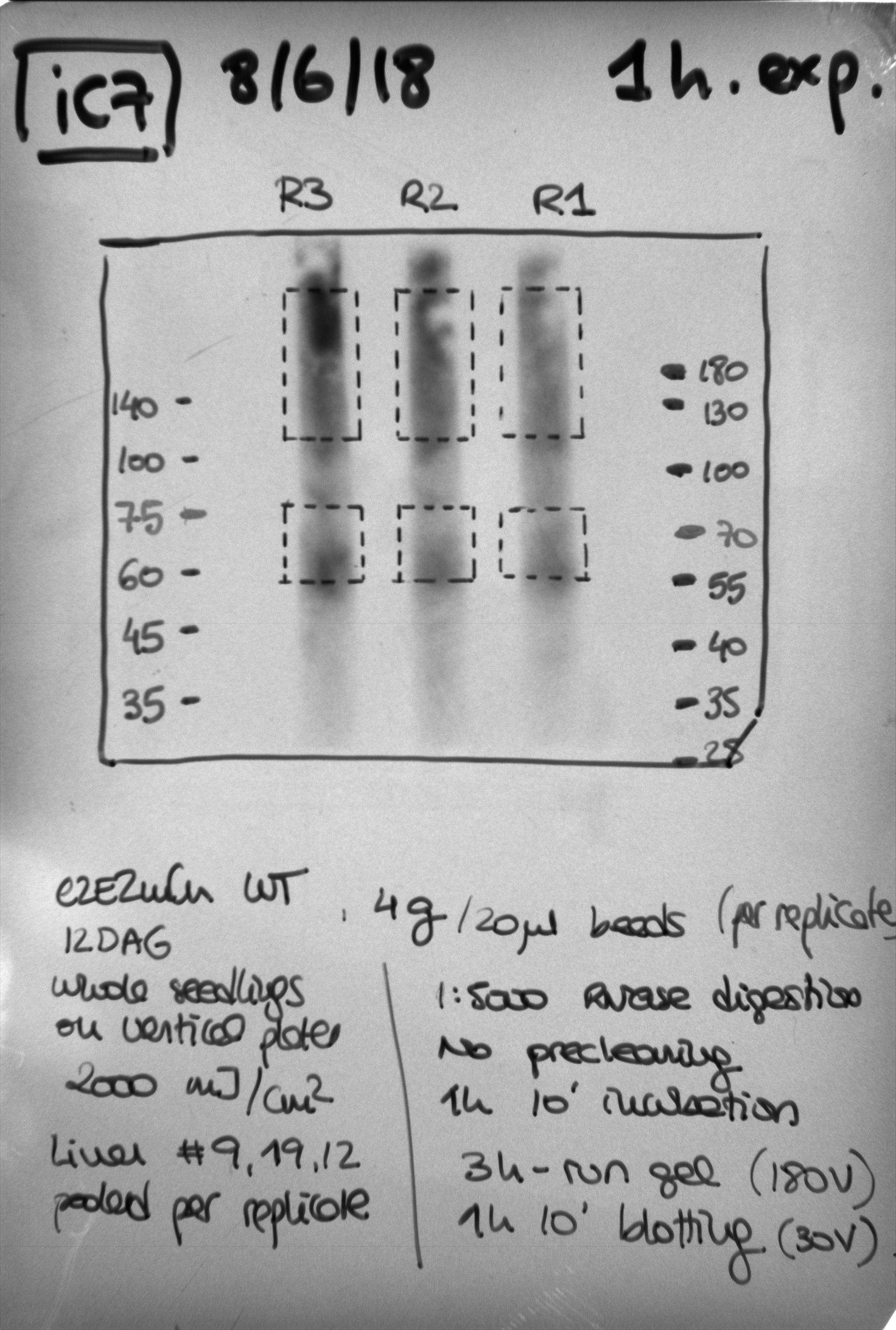

Supplement: Figure 3—figure supplement 3—source data 1. [file elife-72375-fig3-figsupp3-data1.zip › ECT2-Targeting_v2_Figure3-Figure_supplement3-Source_data2.tif]

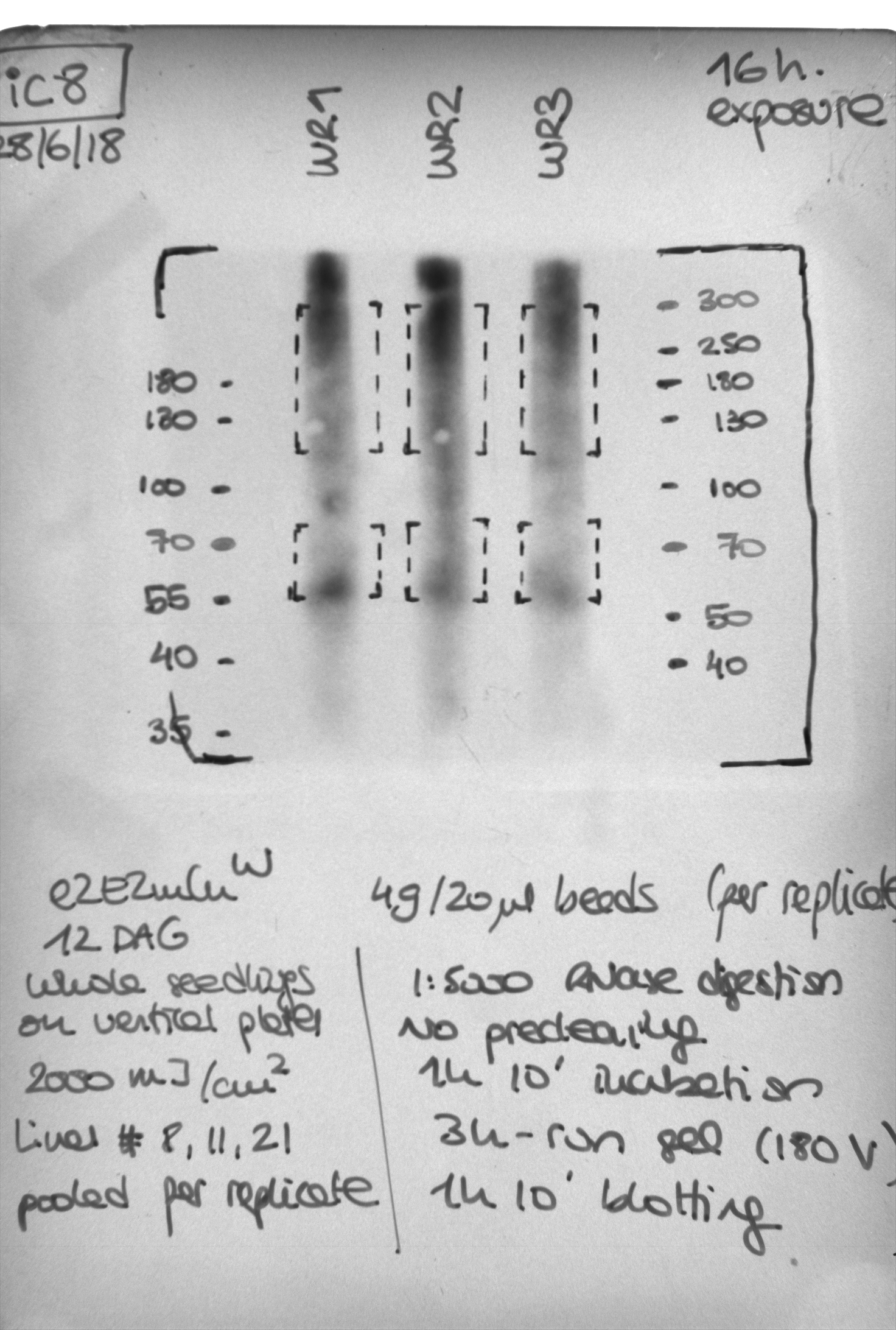

Supplement: Figure 3—figure supplement 3—source data 1. [file elife-72375-fig3-figsupp3-data1.zip › ECT2-Targeting_v2_Figure3-Figure_supplement3-Source_data3.tif]

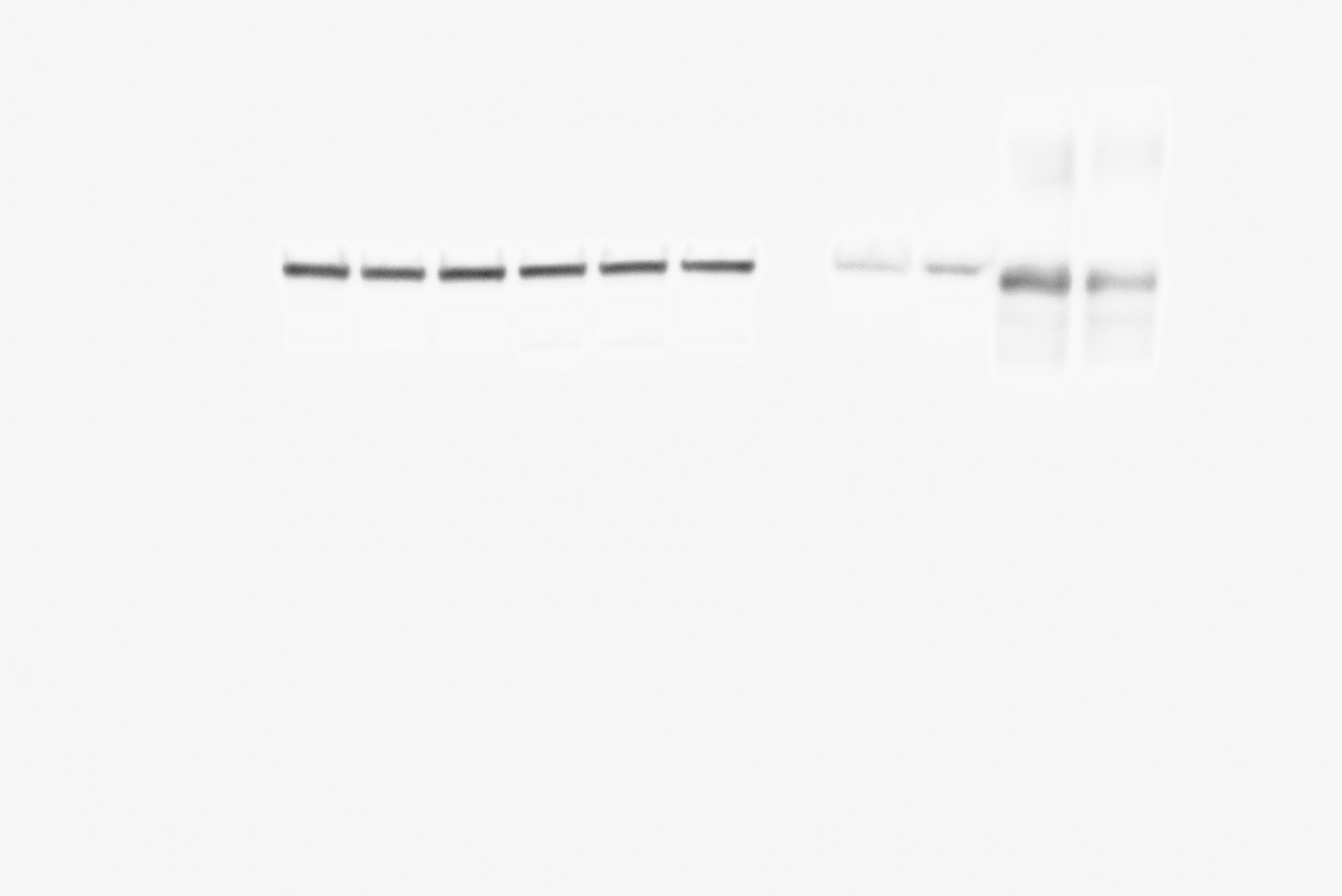

Supplement: Figure 3—figure supplement 3—source data 1. [file elife-72375-fig3-figsupp3-data1.zip › ECT2-Targeting_v2_Figure3-Figure_supplement3-Source_data4.tif]

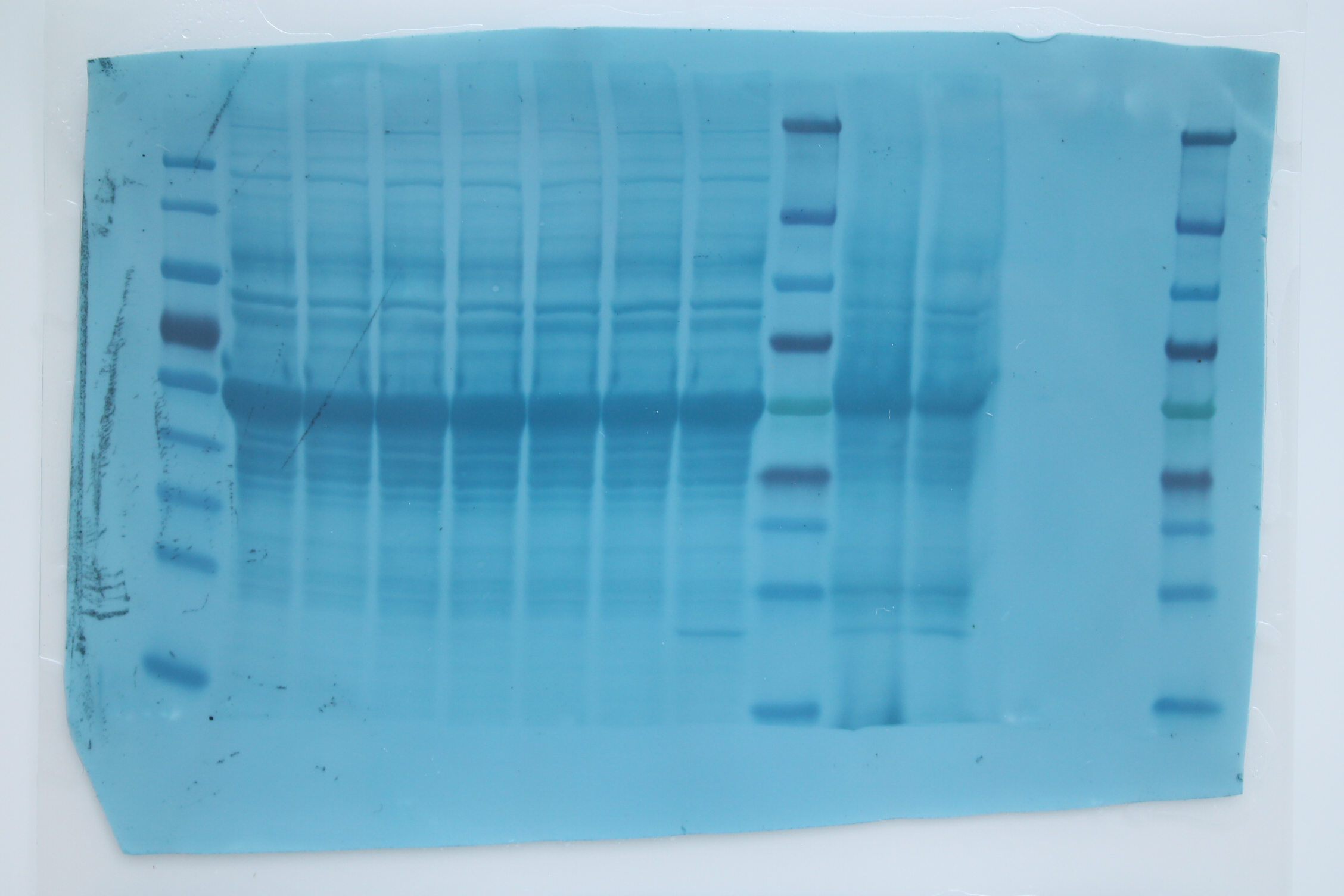

Supplement: Figure 3—figure supplement 3—source data 1. [file elife-72375-fig3-figsupp3-data1.zip › ECT2-Targeting_v2_Figure3-Figure_supplement3-Source_data5.JPG]

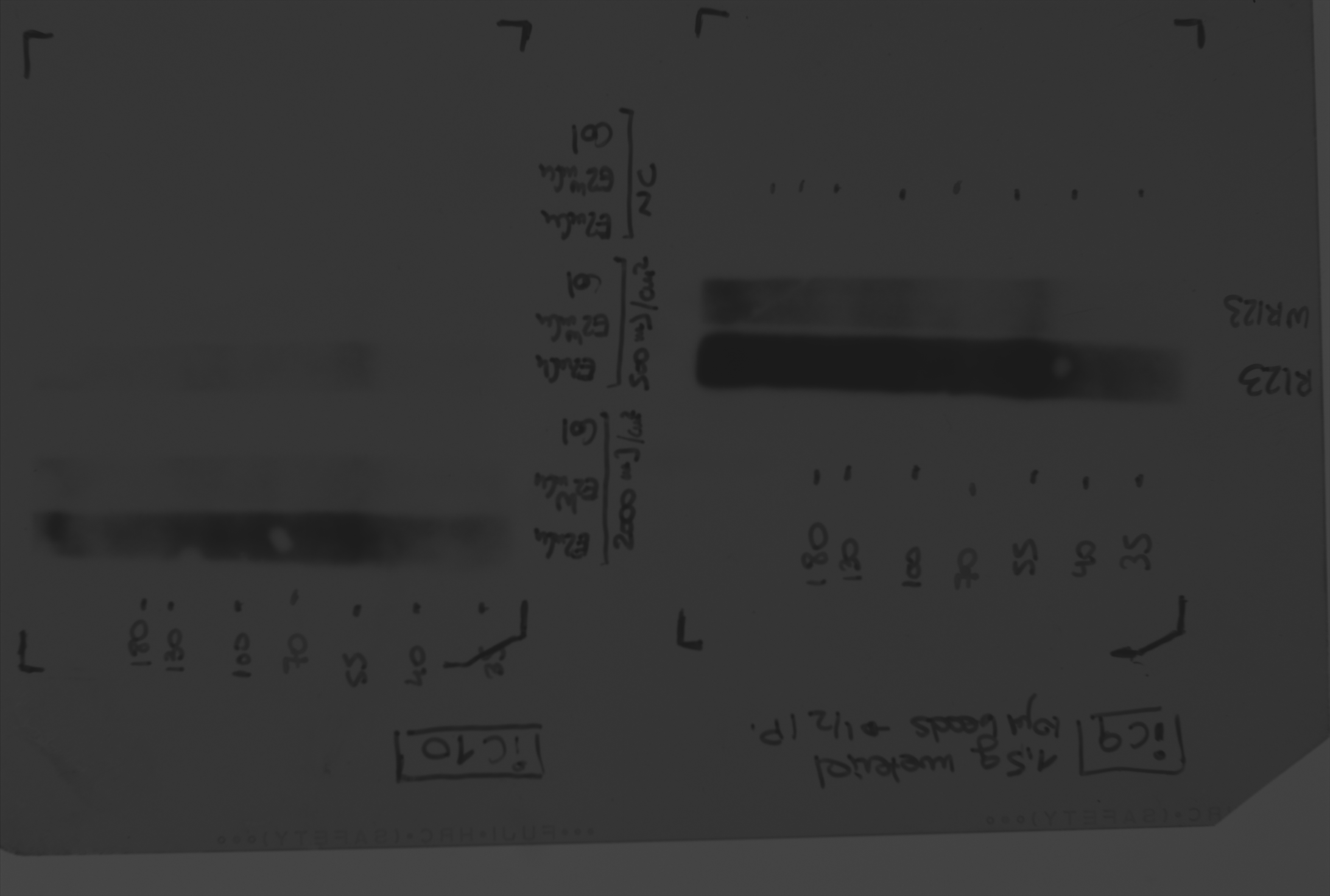

Supplement: Figure 3—figure supplement 3—source data 1. [file elife-72375-fig3-figsupp3-data1.zip › ECT2-Targeting_v2_Figure3-Figure_supplement3-Source_data6.tif]

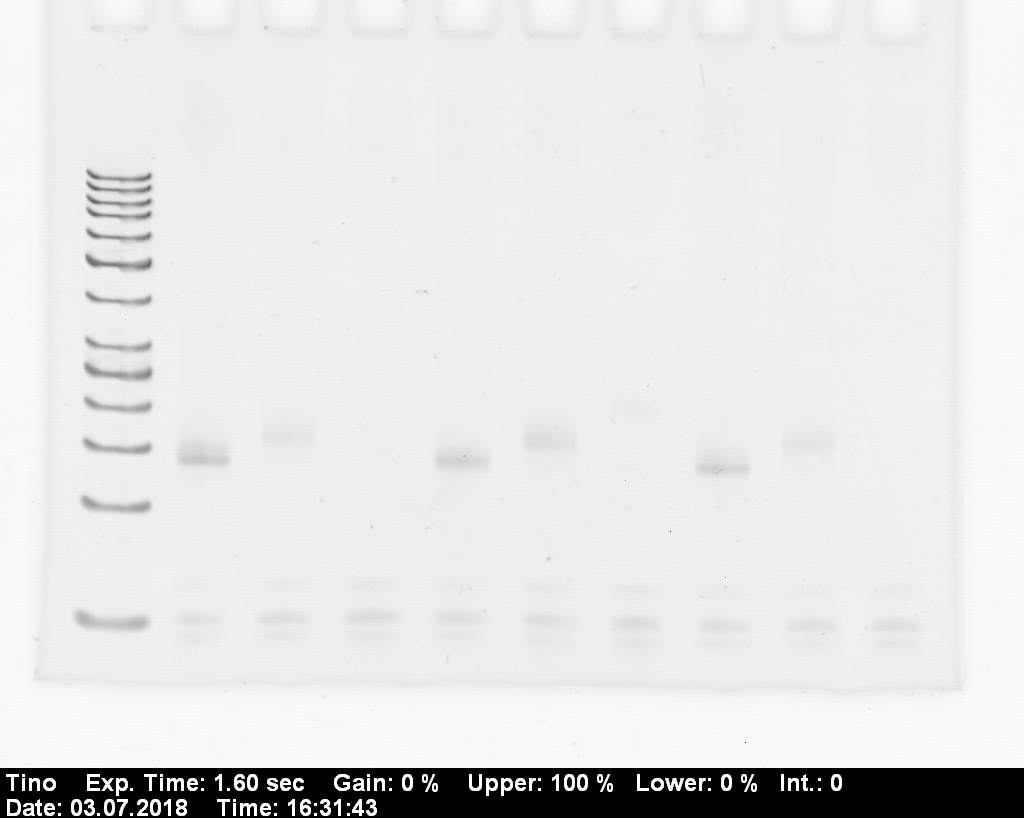

Supplement: Figure 3—figure supplement 3—source data 1. [file elife-72375-fig3-figsupp3-data1.zip › ECT2-Targeting_v2_Figure3-Figure_supplement3-Source_data7.jpg]

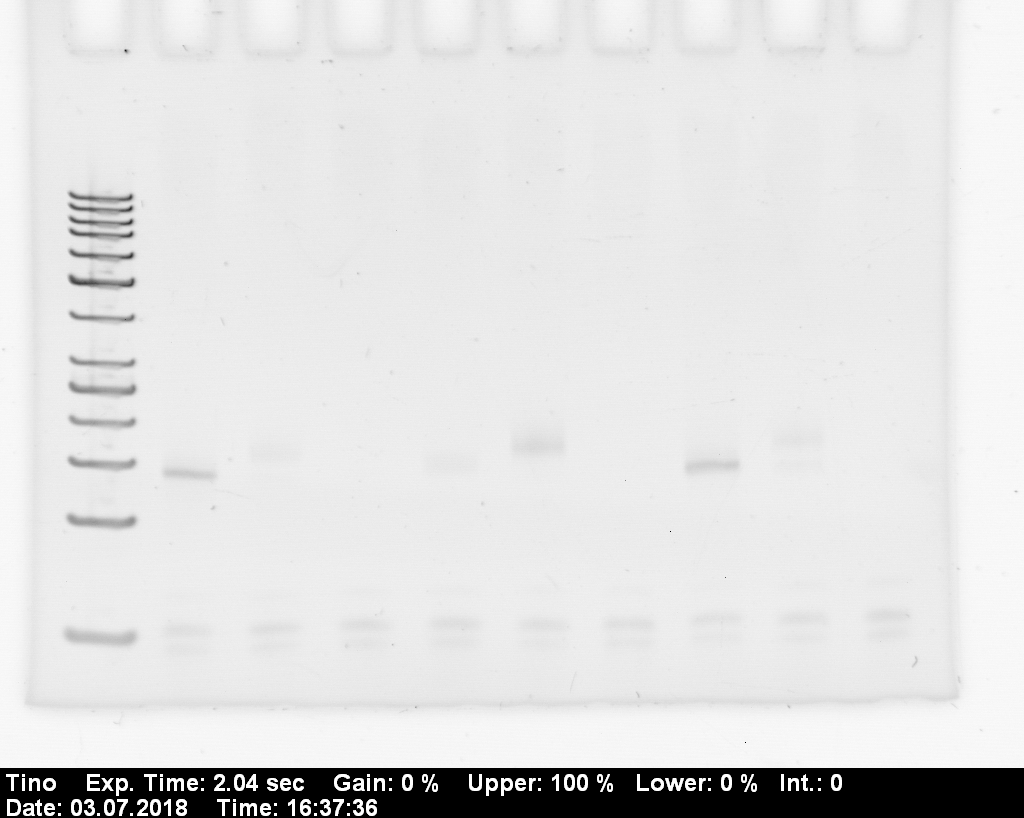

Supplement: Figure 3—figure supplement 3—source data 1. [file elife-72375-fig3-figsupp3-data1.zip › ECT2-Targeting_v2_Figure3-Figure_supplement3-Source_data8.jpg]

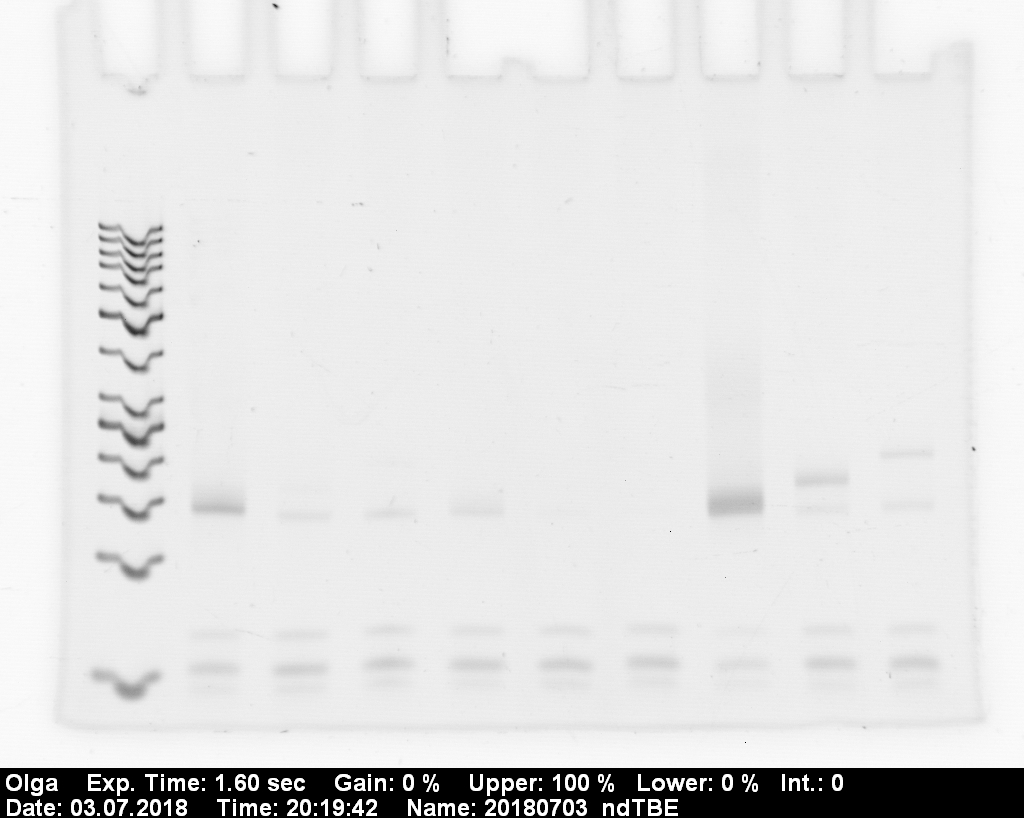

Supplement: Figure 3—figure supplement 3—source data 1. [file elife-72375-fig3-figsupp3-data1.zip › ECT2-Targeting_v2_Figure3-Figure_supplement3-Source_data9.jpg]
