# Supplementary material for: Principles of mRNA targeting via the Arabidopsis m6A-binding protein ECT2
Source: eLife. 2021 Sep 30;10:e72375. doi: 10.7554/eLife.72375 (PMC8796052; doi:10.7554/eLife.72375)

Figure 3—SourceData1

Dotted outlines indicate the cropping applied to the figure

Figure Panels

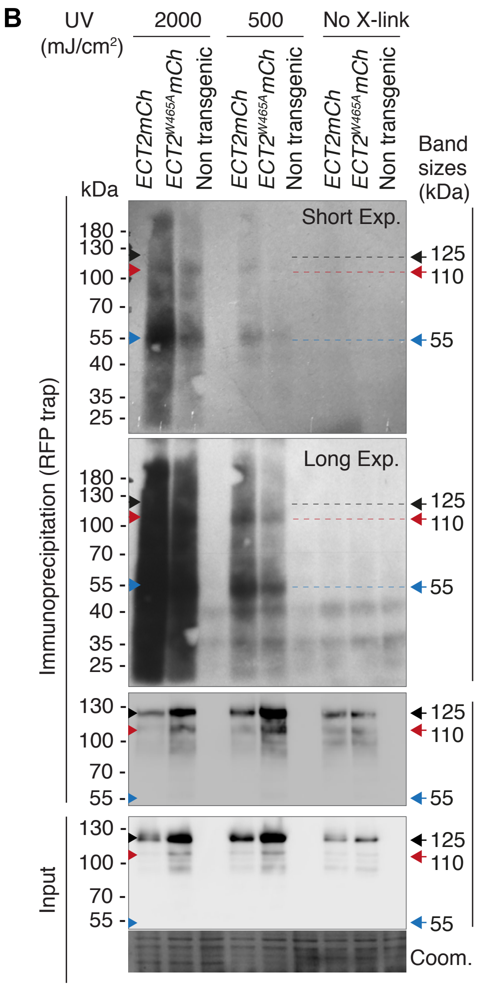

Autoradiograms (IP)

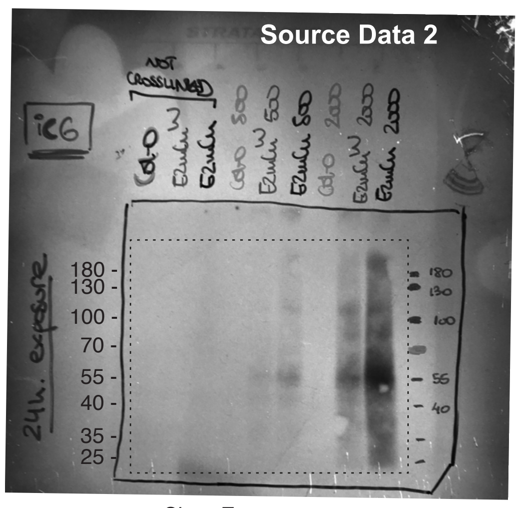

Short Exposure

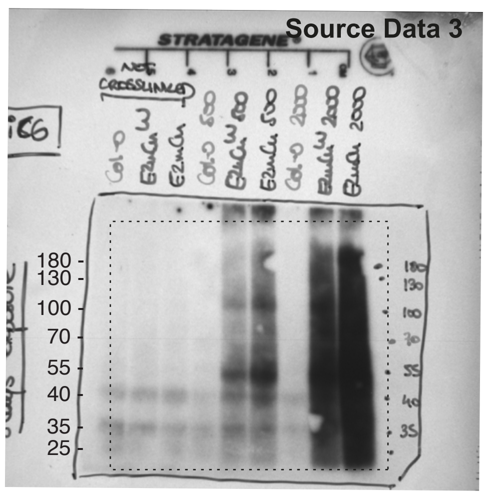

Long Exposure

Notice that the loading order has been mirrored left-to-right in the final figure to restore the original loading order that became mirrored during blotting and exposure to the film

α–mCherry Western blot (IP)

Enhanced Chemiluminescence

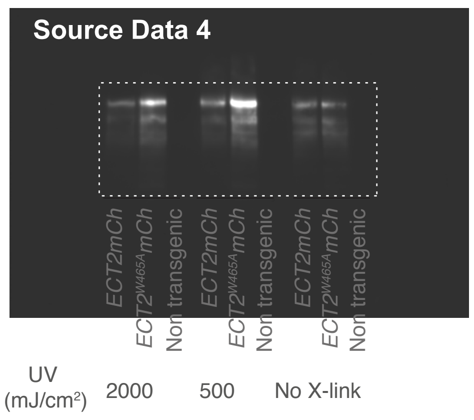

Bright field (ladder)

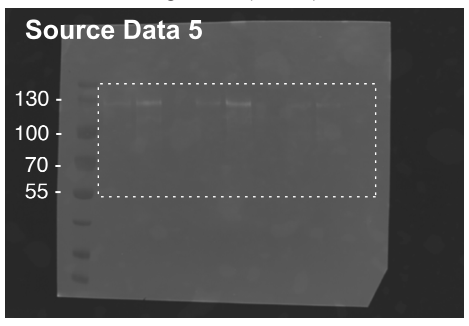

α–mCherry Western blot (input)

Enhanced Chemiluminescence

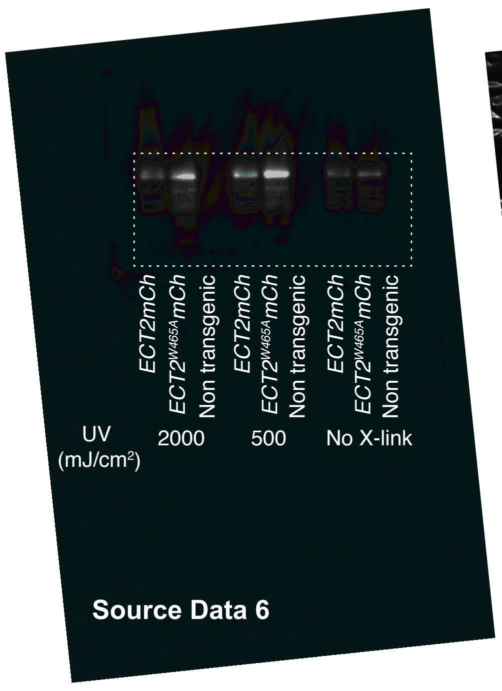

Bright field (ladder)

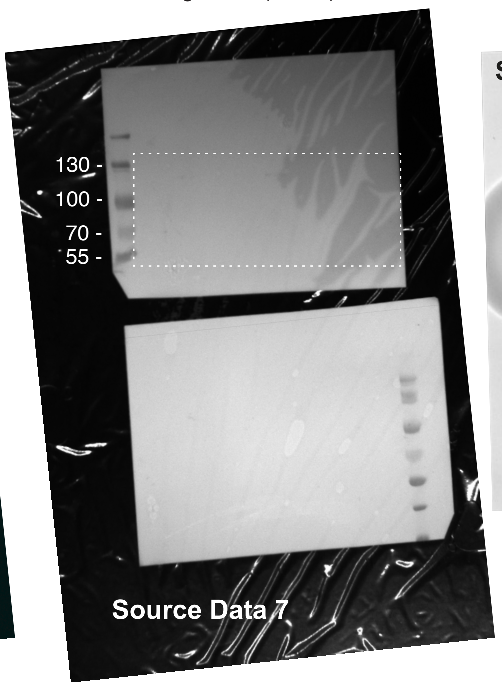

Coomassie (loading)

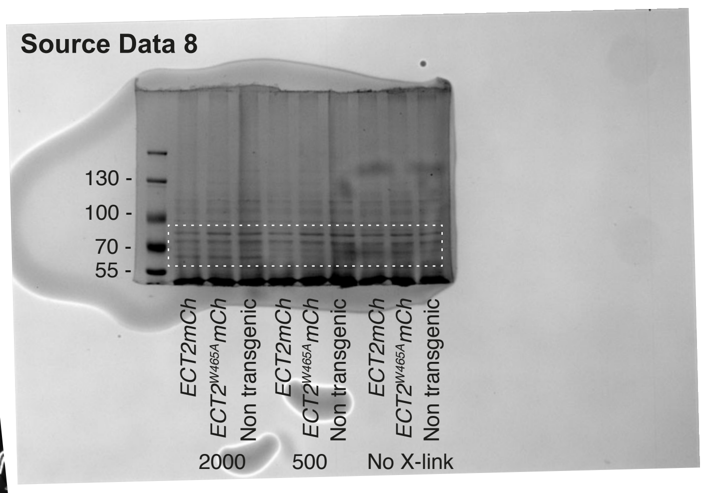

Figure Panels

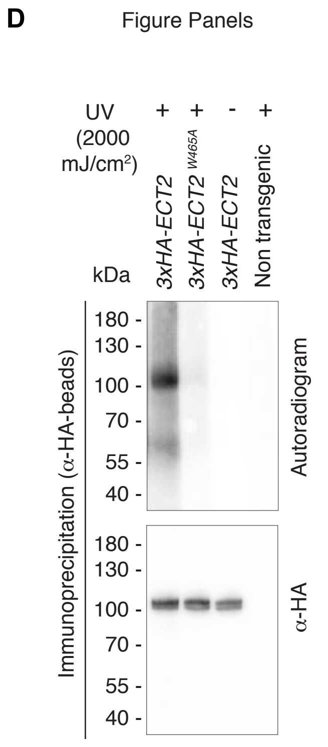

Autoradiograms (IP)

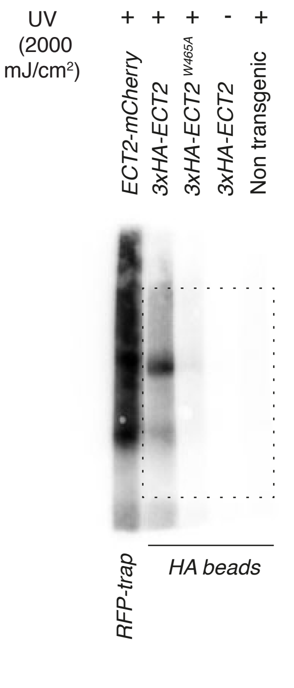

Source Data 9

α–HA Western blot (IP)

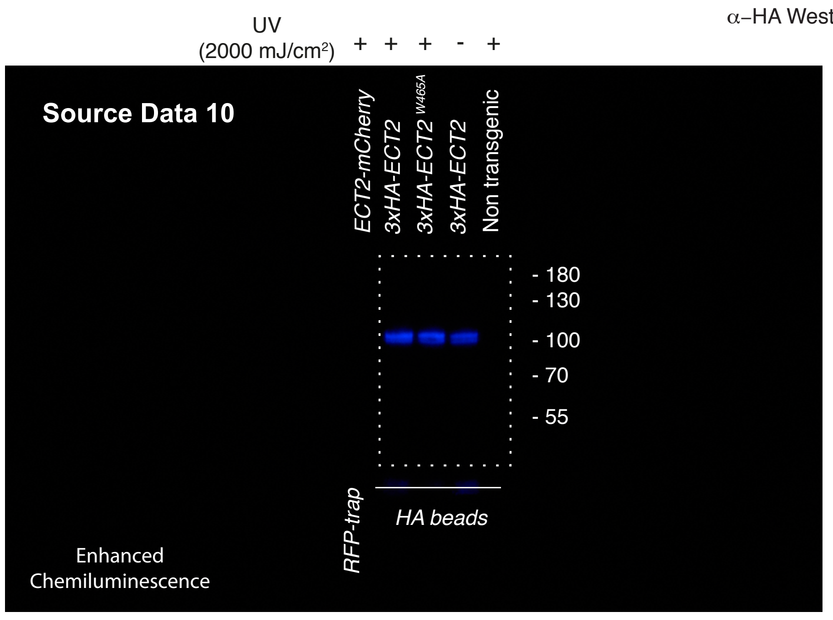

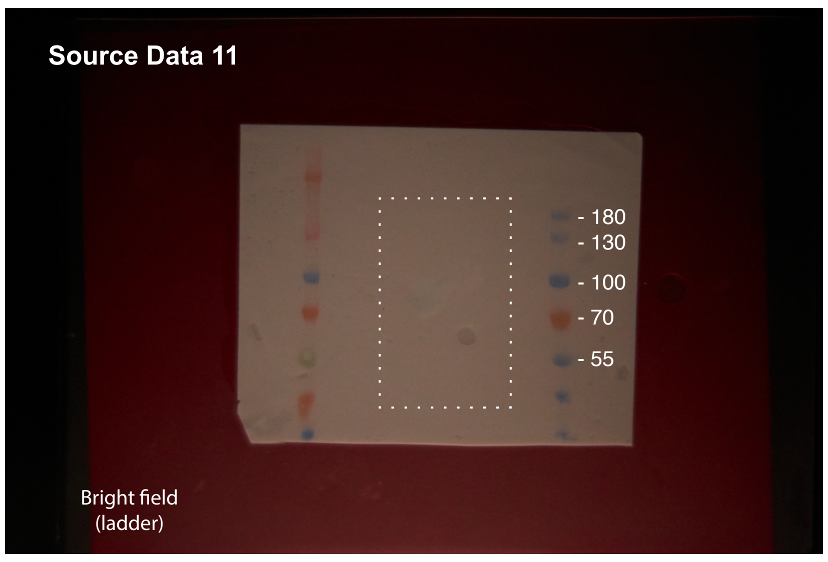

Supplement: Figure 3—source data 1. [file elife-72375-fig3-data1.zip › ECT2-Targeting_v2_Figure3-Source_data_1.pdf]
