# Supplementary material for: Principles of mRNA targeting via the Arabidopsis m6A-binding protein ECT2
Source: eLife. 2021 Sep 30;10:e72375. doi: 10.7554/eLife.72375 (PMC8796052; doi:10.7554/eLife.72375)

Figure 3—Figure supplement 1—SourceData1

Dotted outlines indicate the cropping applied to the figure

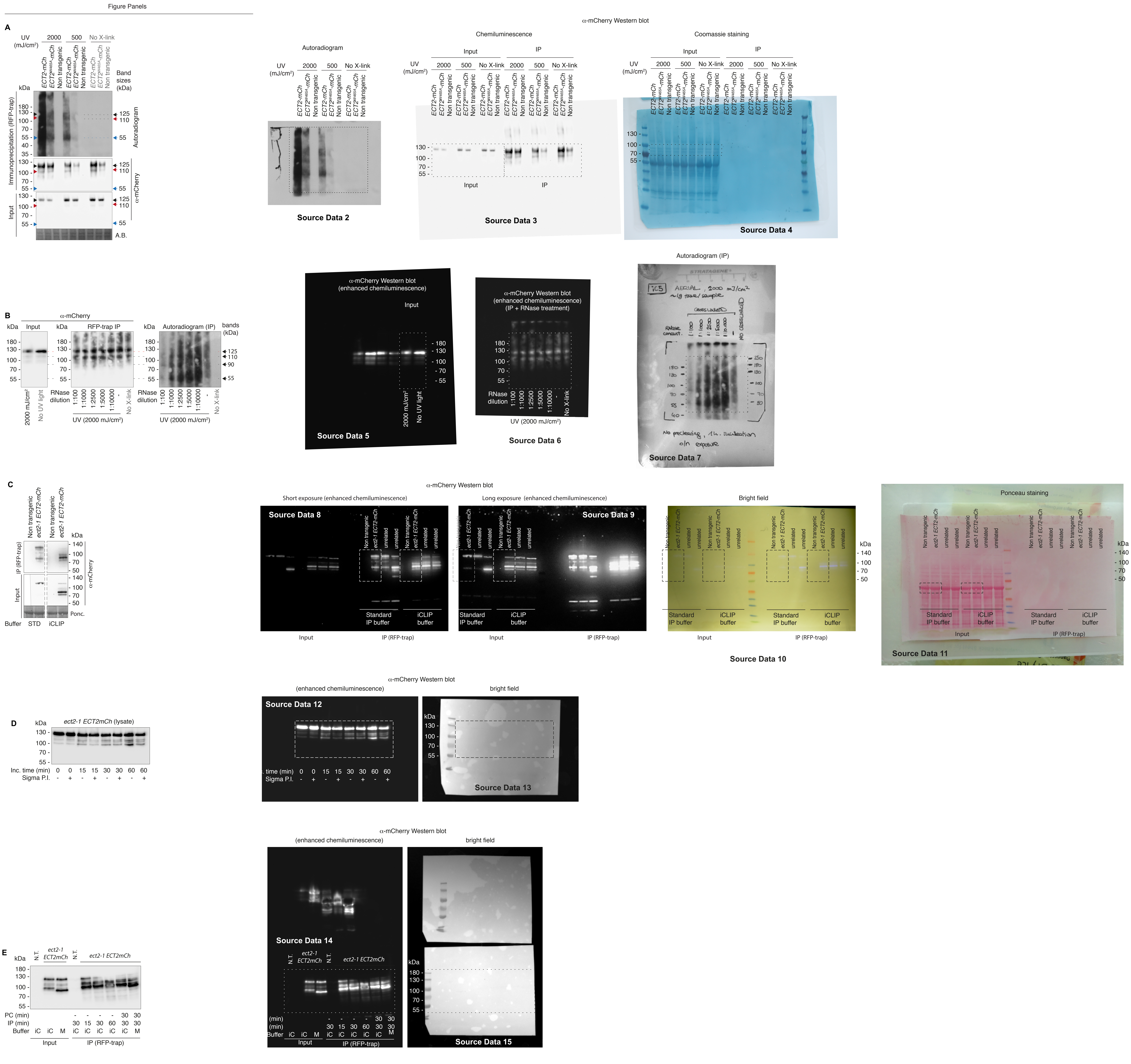

Supplement: Figure 3—figure supplement 1—source data 1. [file elife-72375-fig3-figsupp1-data1.zip › ECT2-Targeting_v2_Figure3-Figure_supplement1-Source_data1.pdf]
