# Supplementary material for: Principles of mRNA targeting via the Arabidopsis m6A-binding protein ECT2
Source: eLife. 2021 Sep 30;10:e72375. doi: 10.7554/eLife.72375 (PMC8796052; doi:10.7554/eLife.72375)

Supplementary File 3. Analysis of HOMER-identified and additional motifs around ECT2-iCLIP and m⁶A sites.

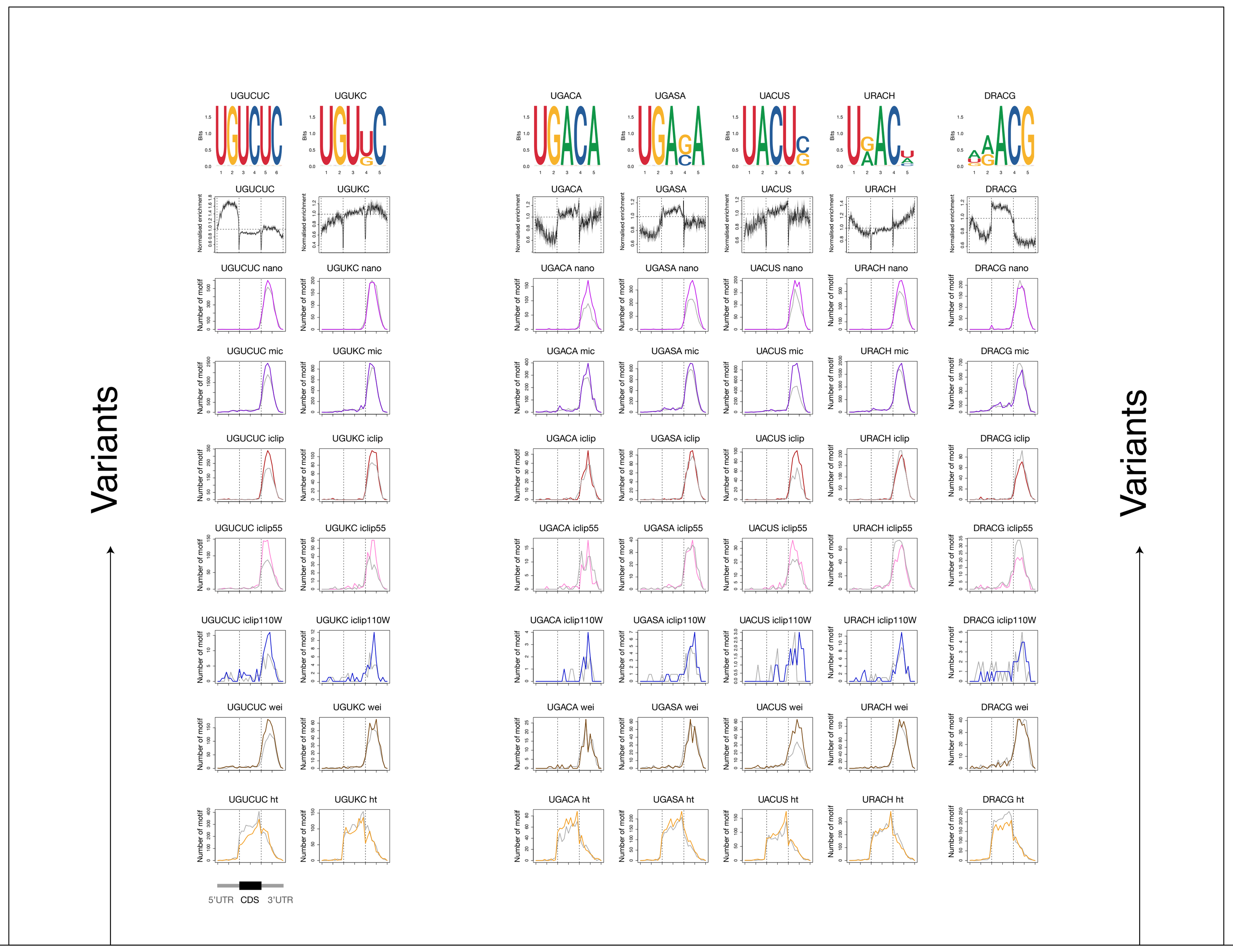

- Upper panels: Position weight matrices (PWM) derived from sequence frequencies of identified motifs around ECT2-iCLIP binding sites.

- Second panels: Normalized enrichment (compared to random sequence) of motifs across the gene body. An enrichment of 1 suggests that the motif is neither over- or under- represented at that location.

- Third-to-bottom panels: Distribution of motif counts by genomic location, for the specified m^{6}A mapping or ECT2-binding datasets (see abbreviations below). For each set, each coordinate representing a site is assigned with a coordinate of equivalent genomic location on a random non-target gene, to create a location-matched background set (plotted in grey). These plots reflect the background counts one would expect to see at the same genomic locations as the foreground set (in most cases within the 3UTR).

nano, Nanopore (Parker et al., 2020)
mic, miCLIP (Parker et al., 2020)
iclip, ECT2-mCherry iCLIP 110-kDa-band
iclip55, ECT2-mCherry iCLIP 55-kDa-band
iclip110W, ECT2^{W464A}-mCherry iCLIP 110-kDa-band
wei, ECT2 FA-CLIP (Wei et al., 2018)
ht, ECT2 HyperTRIBE

Example:

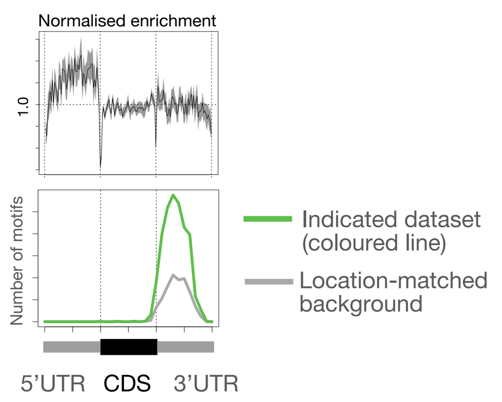

PAS signal

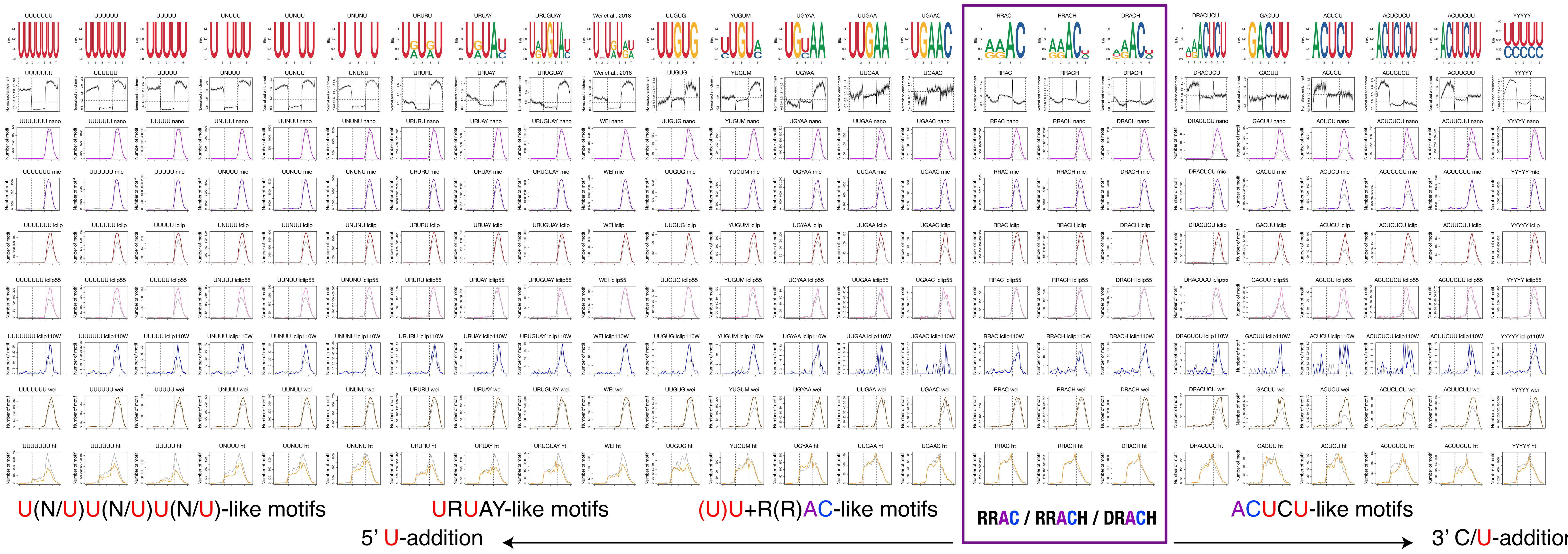

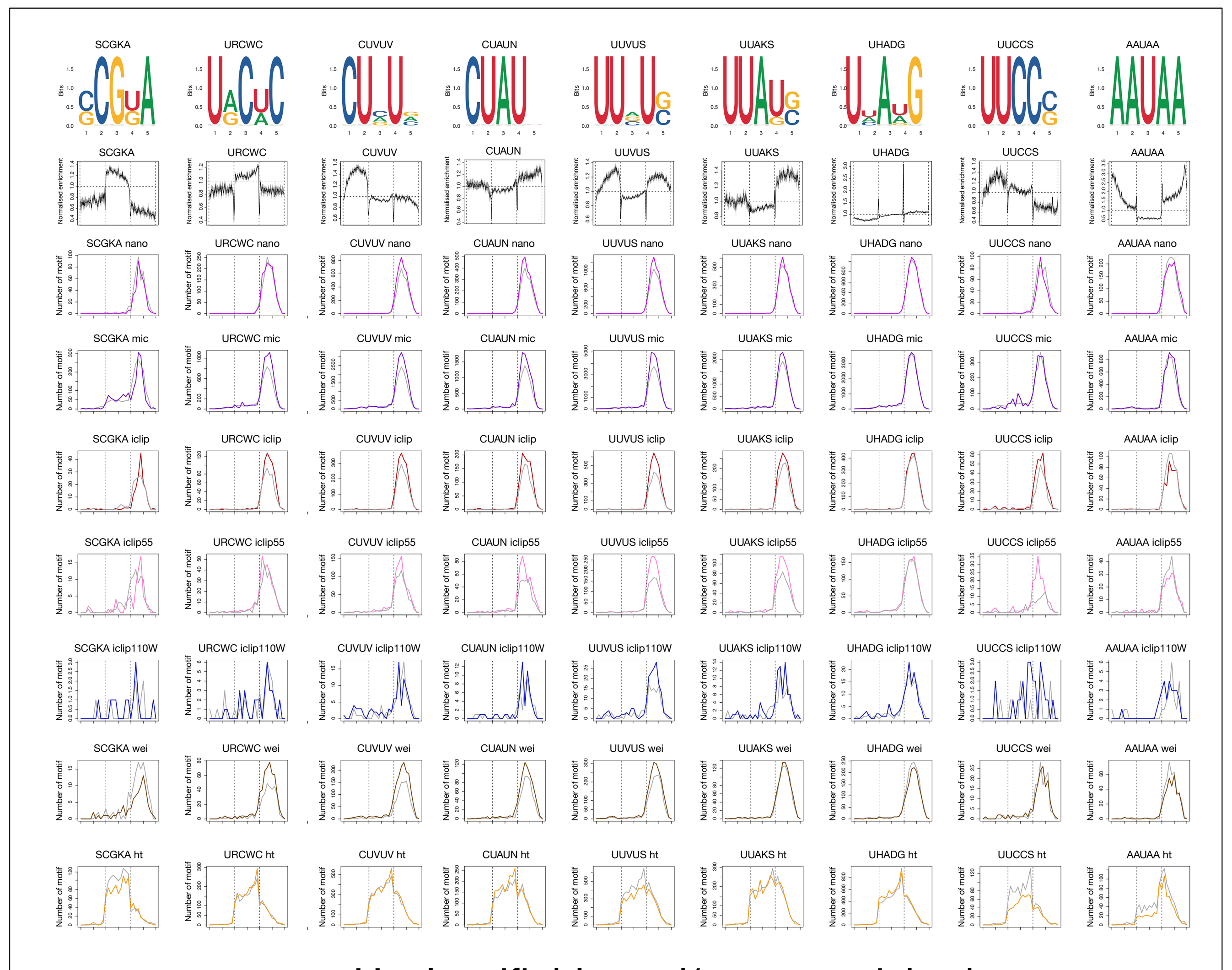

Motifs have been sorted, to facilitate their interpretation, according to:

- 1. RRACH-likeness, paying special attention to 5' or 3' extensions that may include only part of RRACH
- 2. Enrichment in m^{6}A and ECT2-binding datasets
- 3. Skewness on Nanopore-m6A-centered plots (see next page)

As a result, we display a middle array of motifs enriched in either m^{6}A or ECT2-iCLIP datasets with RRACH-like motifs (centre-right, outlined) with U-containing 5' or 3' extensions to both sides. To the left, we placed URU-containing motifs that become prevalently enriched 5' of m^{6}A sites and display ECT2-iCLIP enrichment. Left of URURU-like motifs, we extend with UNUNU/UUUUU sequences that do not contain traces of RRACH and their enrichment in the periphery of m^{6}A sites becomes symmetric. Therefore, they could be placed on either the left-most or right part of the group. Above the middle array we aligned variants of the same motifs, typically with less enrichment.
In the lower panels we placed GGAU-like motifs, with enrichment and symmetry around Nanopore-m^{6}A sites similar to RRAC, for which believe that it can be a bona-fide methylation site. Of note, miCLIP and ECT2-iCLIP enrichment is higher for GGAU-like than for RRAC-like motifs, likely due to the presence of a highly-reactive U nucleotide.
Finally, right to the middle array we show motifs for which the enrichment is small or we could not classify within a group or succession. Notice the presence among them of the poly(A) site (PAS) signal, not enriched in any dataset.

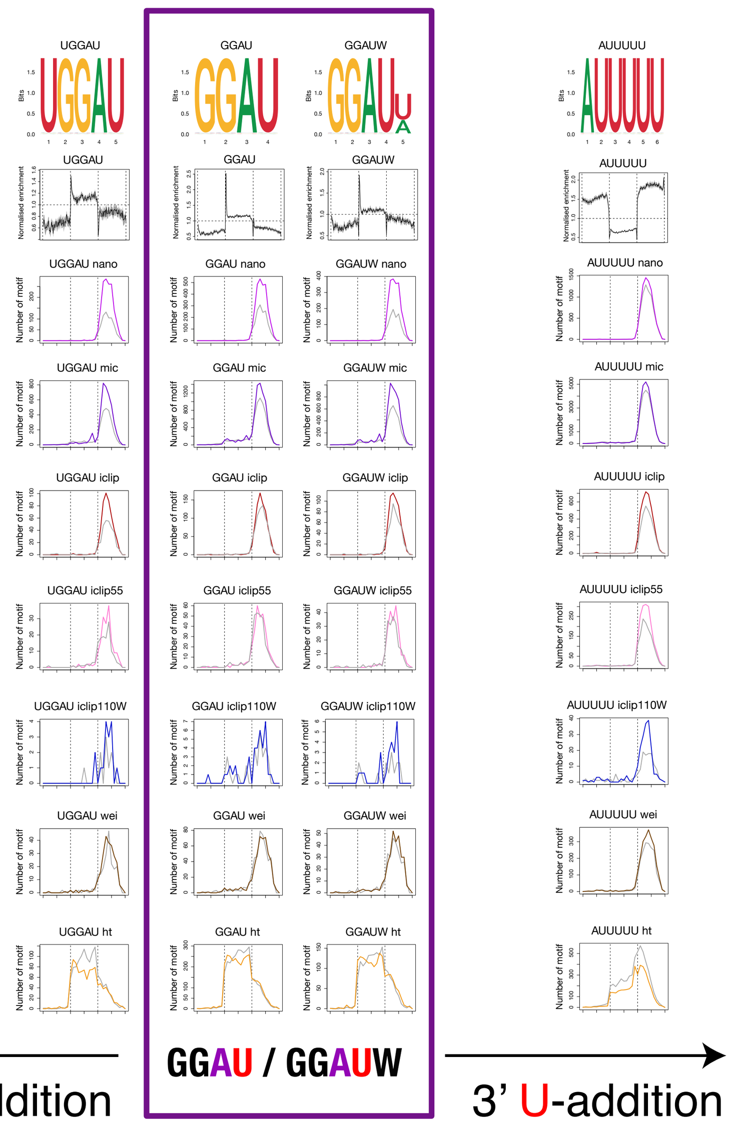

DRACH + GGAU = DRAY

and variants

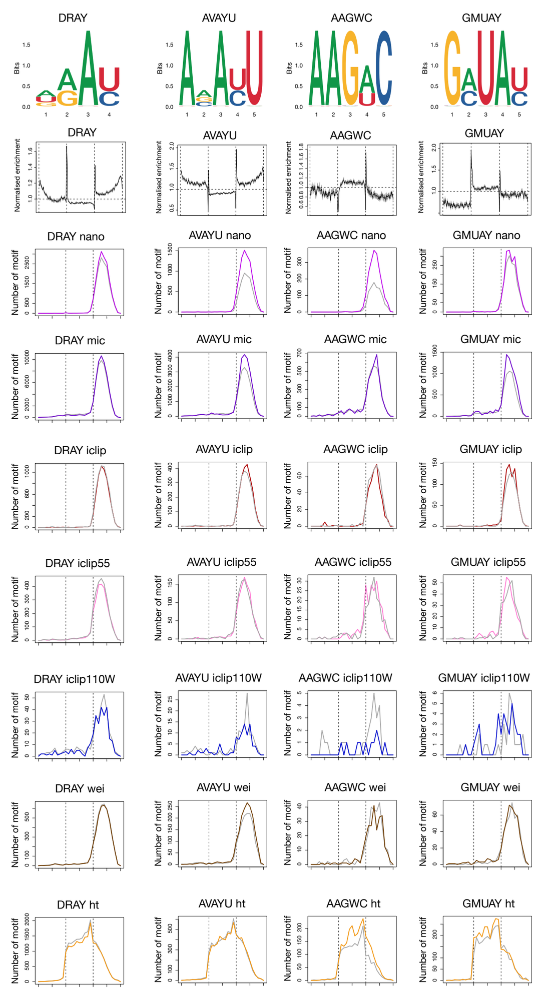

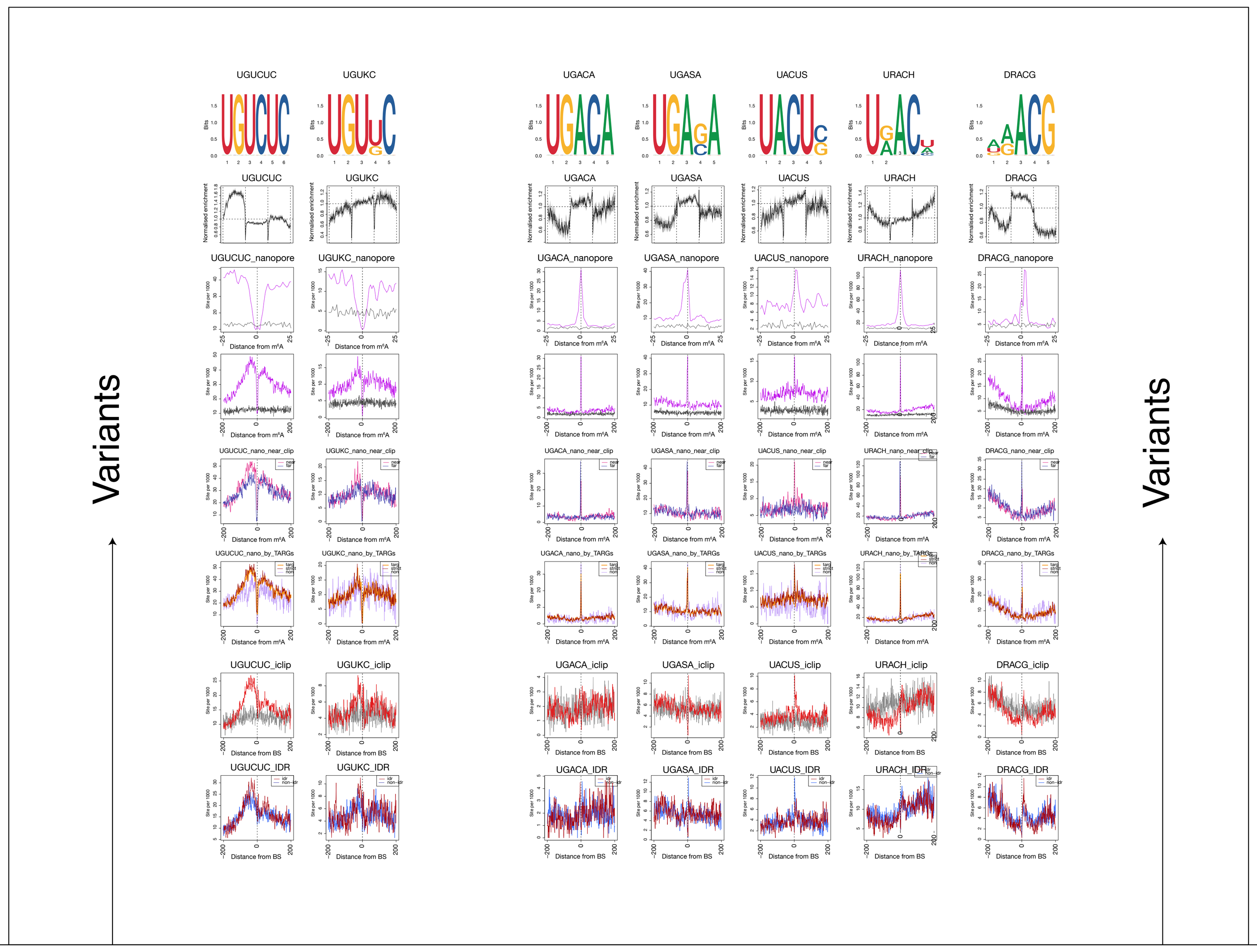

- **Upper panels:** Position weight matrices (PWM) derived from sequence frequencies of identified motifs around ECT2-iCLIP binding sites.
- **Second panels:** Normalized enrichment (compared to random sequence) of motifs across the gene body. An enrichment of 1 suggests that the motif is neither over- or under- represented at that location.
- **3rd, 4th and 8th (from the top) panels:** Number of motifs per 1000 nanopore-defined m^{6}A sites (Parker et al., 2020) or ECT2-iCLIP-peaks (coloured lines), plotted as a function of distance from the defined coordinate at the (center), typically given in a range of -200 bp upstream to 200 bp downstream. To adjust for the potential for downstream regions over-shooting the end of the 3UTR, at each given distance only sites which continue to overlap an annotated gene (Araport11) are counted. Grey lines correspond to the location-matched background set.
- **5th-7th (from the top) and bottom panels:** In some plots, genes containing either nanopore-m^{6}A or ECT2-iCLIP sites have been split into groups and plotted separately according to categories of interest as indicated below.

nano_near_clip — *near* — *far*

m^{6}A sites are split according to whether the nearest iCLIP peak is >25 bp (*far*) or <25bp (*near*)

nano_by_TARGs — *strict* — *targ* — *non*

m^{6}A sites are split according to whether they sit on an ECT2 stringent target (*strict*), i.e. identified by iCLIP and HyperTRIBE, a permissive target (*targ*) identified by 1 or 2 methods, or on a transcript not identified as an ECT2-target by either method (*non*)

IDR — *idr* — *non–idr*

ECT2-iCLIP binding sites are split according to whether they are found in libraries from both 110 kDa (full-length protein) and 55 kDa (devoid of the N-terminal IDR) bands (*non-idr*), or exclusively in the 110 kDa band (*idr*).

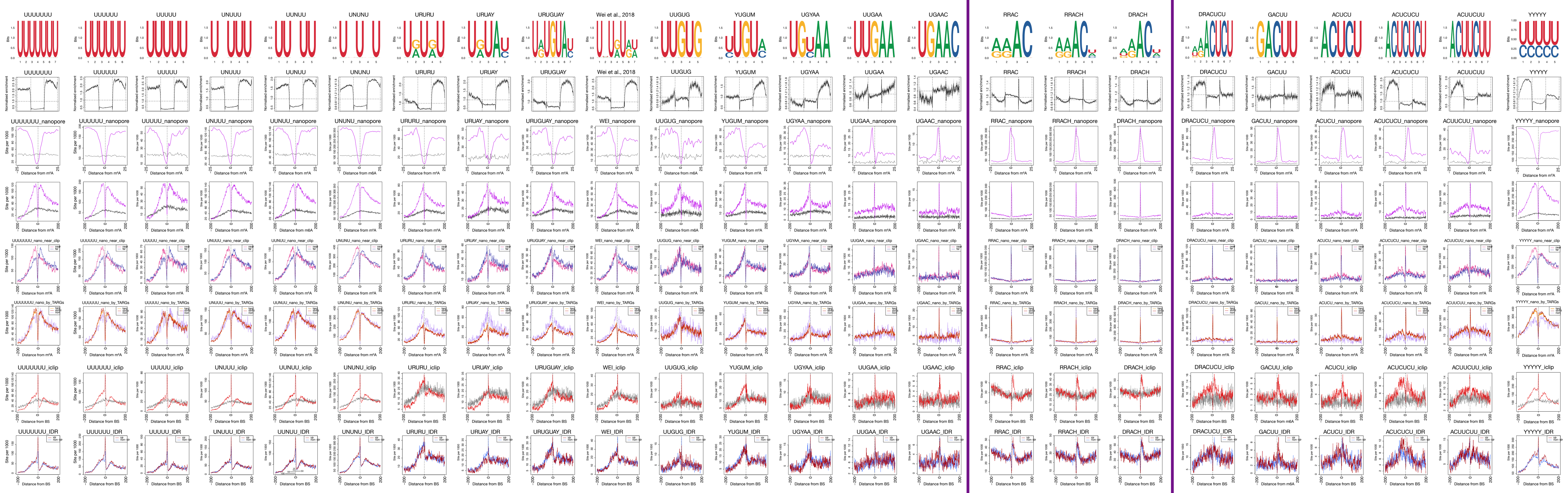

U(N/U)U(N/U)U(N/U)-like motifs — URUAY-like motifs — (U)U+R(R)AC-like motifs — **RRAC / RRACH / DRACH** — ACUCU-like motifs

5’ U-addition ← → 3’ C/U-addition

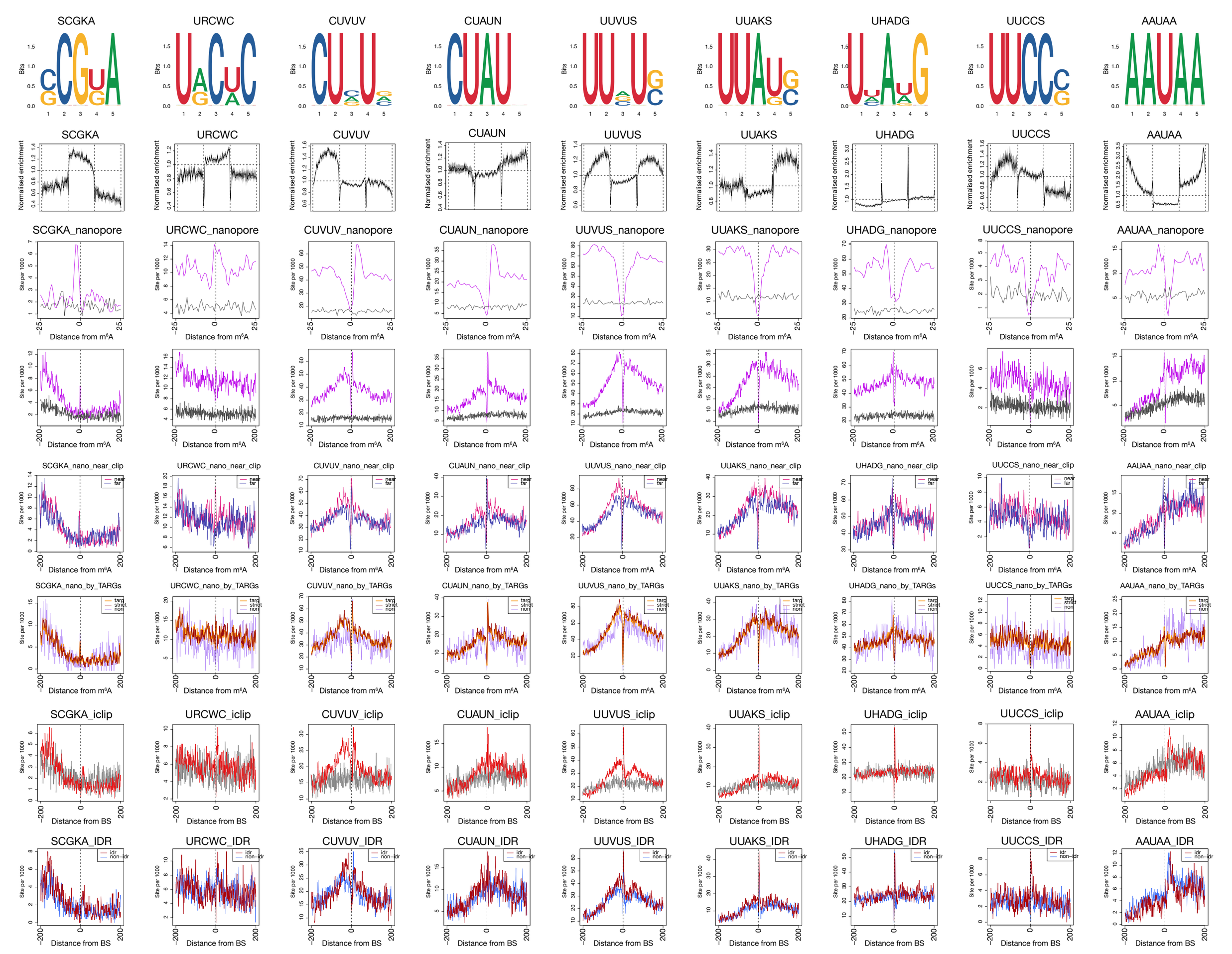

Unclassifiable and/or not enriched

Motifs have been sorted, to facilitate their interpretation, according to:

- 1. RRACH-likeness, paying special attention to 5’ or 3’ extensions that may include only part of RRACH
- 2. Enrichment in m^{6}A and ECT2-binding datasets (see previous page)
- 3. Skewness on Nanopore-m6A-centered plots

As a result, we display a middle array of motifs enriched in either m^{6}A or ECT2-iCLIP datasets with RRACH-like motifs (centre-right, outlined) with U-containing 5’ or 3’ extensions to both sides. To the left, we placed URU-containing motifs that become prevalently enriched 5’ of m^{6}A sites and display ECT2-iCLIP enrichment. Left of URURU-like motifs, we extend with UNUNU/UUUUU sequences that do not contain traces of RRACH and their enrichment in the periphery of m^{6}A sites becomes symmetric. Therefore, they could be placed on either the left-most or right part of the group. Above the middle array we aligned variants of the same motifs, typically with less enrichment.

In the lower panels we placed GGAU-like motifs, with enrichment and symmetry around Nanopore-m^{6}A sites similar to RRAC, for which believe that it can be a bona-fide methylation site. Of note, miCLIP and ECT2-iCLIP enrichment is higher for GGAU-like than for RRAC-like motifs, likely due to the presence of a highly-reactive U nucleotide.

Finally, right to the middle array we show motifs for which the enrichment is small or we could not classify within a group or succession. Notice the presence among them of the poly(A) site (PAS) signal, not enriched in any dataset.

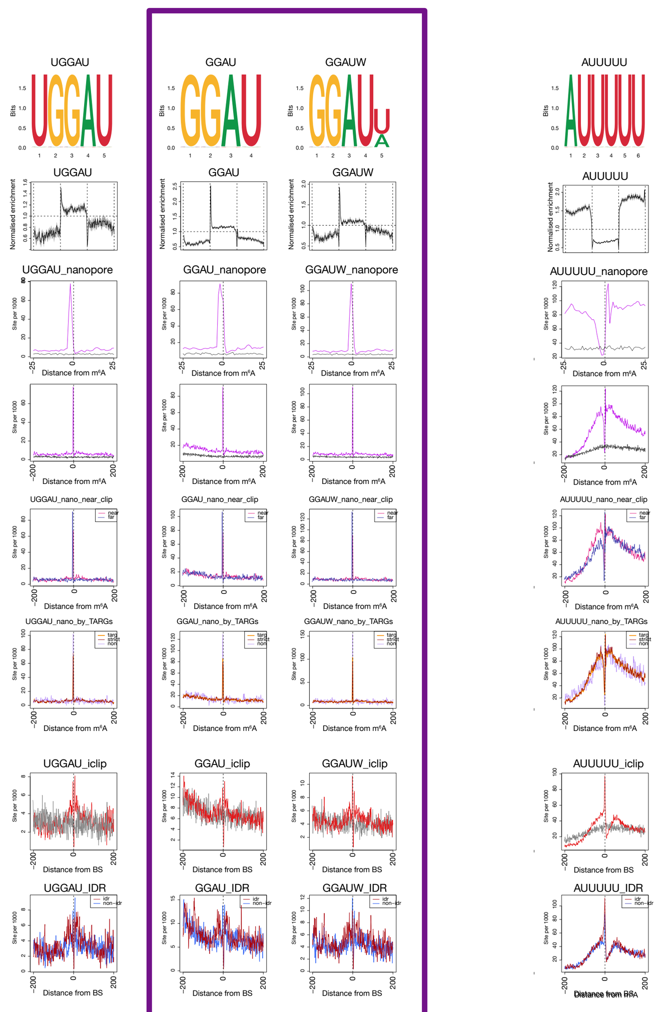

GGAU / GGAUW

5’ U-addition ← → 3’ U-addition

DRACH + GGAU = DRAY

and variants

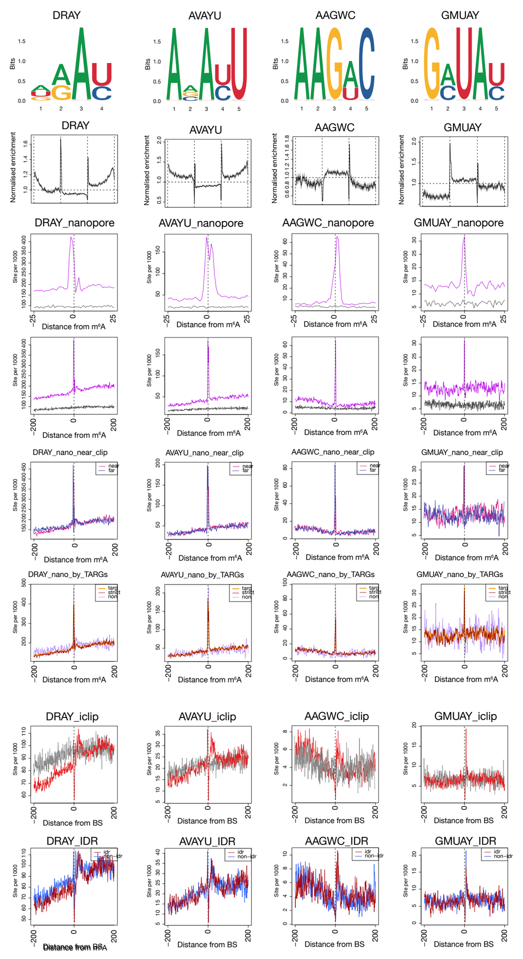

Supplement: Supplementary file 3. [file elife-72375-supp3.pdf]
