# Supplementary material for: Principles of mRNA targeting via the Arabidopsis m6A-binding protein ECT2
Source: eLife. 2021 Sep 30;10:e72375. doi: 10.7554/eLife.72375 (PMC8796052; doi:10.7554/eLife.72375)
Supplement: Figure 7—figure supplement 1—source data 1. [file elife-72375-fig7-figsupp1-data1.pdf]

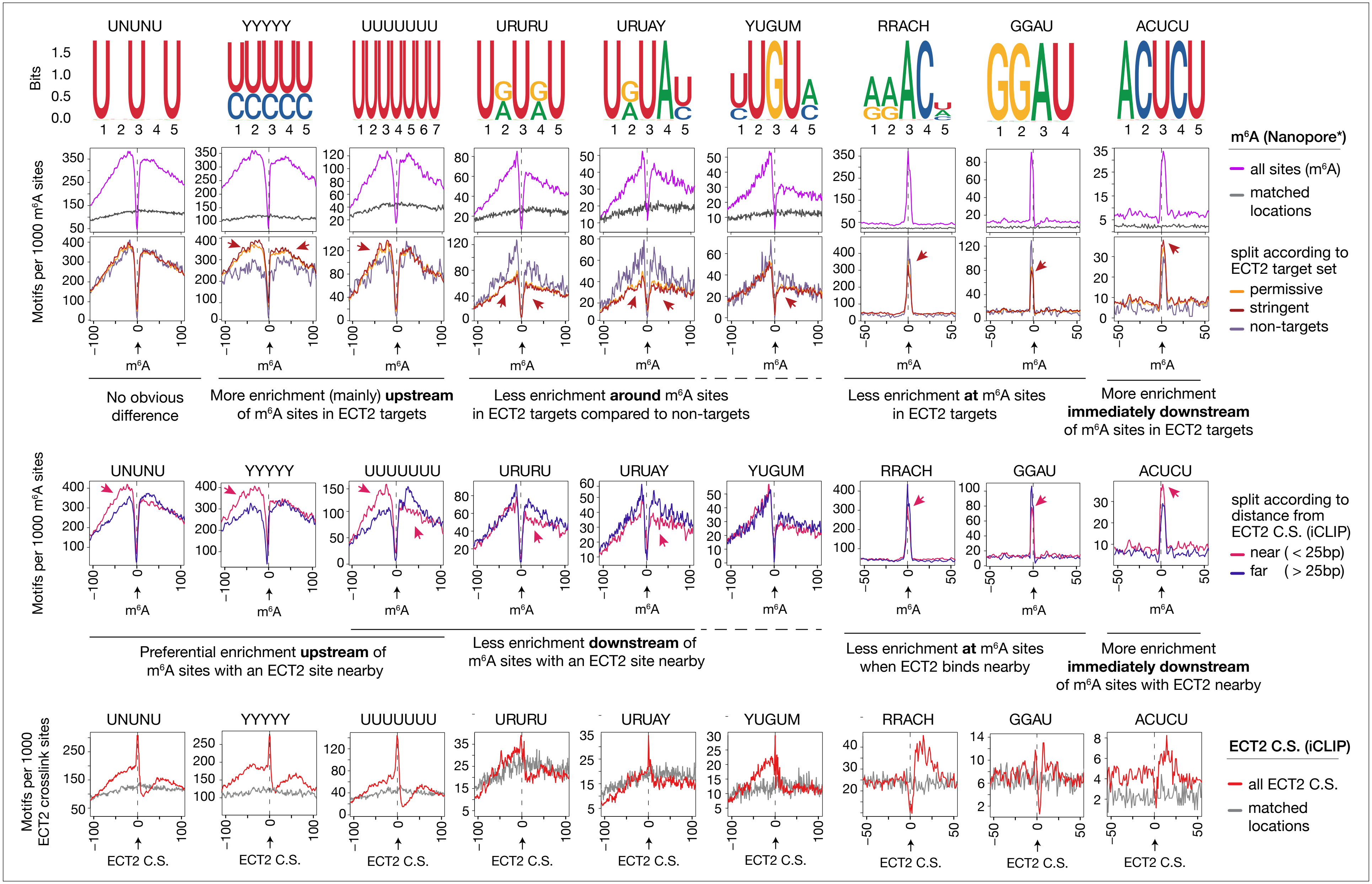

Figure 7—figure supplement 1. Motif preferences around m⁶A sites according to ECT2 binding. From top to bottom: (1) motif logos for derived position weight matrices (PWMs); (2) distance-based enrichment of motifs at and around m⁶A-Nanopore* sites, plotted as motif counts per 1000 m⁶A sites (purple lines). Grey lines indicate the enrichment in a location-matched background set as in Figure 5D; (3) same as in 2 with sites split according to whether they sit on ECT2 targets; (4) same as in 2 with sites split according to the distance from the nearest ECT2 crosslink site (for ECT2-iCLIP targets only); (5) motif counts per 1000 iCLIP binding sites, as a function of distance from the iCLIP position, showing all sites against matched background sites (grey lines). Motifs are ordered according to the different relative enrichment upstream or downstream m⁶A sites according to ECT2 binding (see text below panels 3 and 4). * Parker et al. (2020).
