# Supplementary material for: Principles of mRNA targeting via the Arabidopsis m6A-binding protein ECT2
Source: eLife. 2021 Sep 30;10:e72375. doi: 10.7554/eLife.72375 (PMC8796052; doi:10.7554/eLife.72375)
Supplement: Figure 7—figure supplement 2—source data 1. [file elife-72375-fig7-figsupp2-data1.pdf]

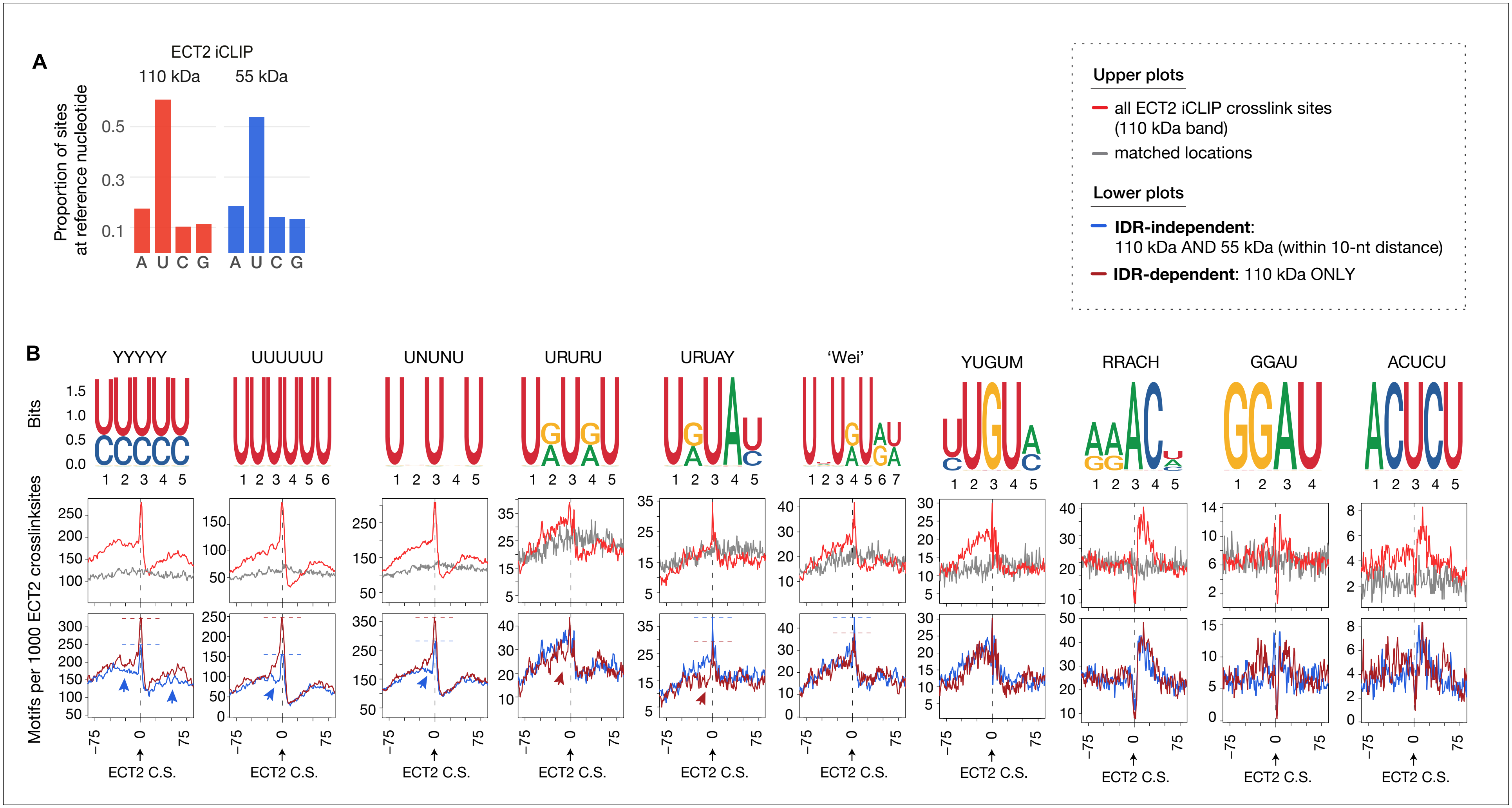

Figure 7—figure supplement 2. Dependency of the ECT2 IDR for motif enrichment. (A) Proportion of ECT2 iCLIP sites at each nucleotide for the 110 kDa and 55 kDa bands. **(B)** From top to bottom: (1) motif logos for derived position weight matrices (PWMs); (2) distribution of motifs per 1000 ECT2 iCLIP crosslink sites (red) or matched-locations background (grey), +/- 75 nt of the site; (3) Motifs per 1000 ECT2 iCLIP crosslink sites, split according to whether they are found in libraries from both 110 kDa and 55 kDa bands ('IDR-independent'), or exclusively (distance > 10 nt) in the 110 kDa band ('IDR-dependent').
