# Supplementary material for: Principles of mRNA targeting via the Arabidopsis m6A-binding protein ECT2
Source: eLife. 2021 Sep 30;10:e72375. doi: 10.7554/eLife.72375 (PMC8796052; doi:10.7554/eLife.72375)
Supplement: Transparent reporting form [file elife-72375-transrepform1.pdf]

eLIFE
1st Floor
24 Hills Road
Cambridge CB2 1JP, UK
P 01223 855340
W elifesciences.org
T @elife

eLife's transparent reporting form

We encourage authors to provide detailed information *within their submission* to facilitate the interpretation and replication of experiments. Authors can upload supporting documentation to indicate the use of appropriate reporting guidelines for health-related research (see EQUATOR Network), life science research (see the BioSharing Information Resource), or the ARRIVE guidelines for reporting work involving animal research. Where applicable, authors should refer to any relevant reporting standards documents in this form.

If you have any questions, please consult our Journal Policies and/or contact us: editorial@elifesciences.org.

Sample-size estimation

- You should state whether an appropriate sample size was computed when the study was being designed
- You should state the statistical method of sample size computation and any required assumptions
- If no explicit power analysis was used, you should describe how you decided what sample (replicate) size (number) to use

Please outline where this information can be found within the submission (e.g., sections or figure legends), or explain why this information doesn't apply to your submission:

For high-throughput sequencing experiments, we did not employ power analysis to decide on the number of replicates.
Information on the number of replicates used for each experiment (HyperTRIBE, and iCLIP) reported is found in Figure 1 and its legend (HyperTRIBE), and in Figure 3-Figure Supplement 3 and its legend (iCLIP). This information is also described in Methods.
For iCLIP, we decided on using 3 replicates simply because this is a commonly used trade-off between sequencing depth and replicates for this type of experiment. For HyperTRIBE, we used 5 different replicates, because we decided to use independent transgenic lines as our replicates to avoid line-specific artifacts, as described in Methods. For this reason, we anticipated that 1, or in the worst of scenarios, 2 samples may be outliers due to transgenic line-specific effects, and therefore decided on using 5 lines to have a high chance of having at least 3 usable replicates. Subsequent analysis showed that the sample corresponding to Line 3 (L3) of *ect2-1 ECT2-FLAG-ADAR* of root tissue had low quality and it was removed from the significance calling pipeline, but all 5 five replicates of aerial tissue were used. The analyses also showed that due to the relatively low editing proportions observed, the additional one or two replicates were useful in the calling of significant sites.

Replicates

- You should report how often each experiment was performed
- You should include a definition of biological versus technical replication
- The data obtained should be provided and sufficient information should be provided to indicate the number of independent biological and/or technical replicates
- If you encountered any outliers, you should describe how these were handled
- Criteria for exclusion/inclusion of data should be clearly stated

eLife Sciences Publications, Ltd is a limited liability non-profit non-stock corporation incorporated in the State of Delaware, USA, with company number 5030732, and is registered in the UK with company number FC030576 and branch number BR015634 at the address 1st Floor, 24 Hills Road, Cambridge CB2 1JP | August 2014

- High-throughput sequence data should be uploaded before submission, with a private link for reviewers provided (these are available from both GEO and ArrayExpress)

Please outline where this information can be found within the submission (e.g., sections or figure legends), or explain why this information doesn't apply to your submission:

Information on the number of times each experiment was performed can be found in Methods or in the legend to figure reporting the results of the experiment.
The terms "experiment", "biological replicate", and technical replicate as used in this paper are defined in the Methods section.
The only sample excluded as an outlier was L3 of *ect2-1 ECT2-FLAG-ADAR* roots, due to low quality (reported in the Methods section).
High-throughput sequencing data have been uploaded to the European Nucleotide Archive (accession number PRJEB44359) and are publicly available, hence no private access token is needed.

Statistical reporting

- Statistical analysis methods should be described and justified
- Raw data should be presented in figures whenever informative to do so (typically when N per group is less than 10)
- For each experiment, you should identify the statistical tests used, exact values of N, definitions of center, methods of multiple test correction, and dispersion and precision measures (e.g., mean, median, SD, SEM, confidence intervals; and, for the major substantive results, a measure of effect size (e.g., Pearson's r, Cohen's d)
- Report exact p-values wherever possible alongside the summary statistics and 95% confidence intervals. These should be reported for all key questions and not only when the p-value is less than 0.05.

Please outline where this information can be found within the submission (e.g., sections or figure legends), or explain why this information doesn't apply to your submission:

Statistical analyses are explained in Methods, and, where appropriate, briefly described in legends. Where appropriate, p-values are given in the Results sections describing the results in question.

(For large datasets, or papers with a very large number of statistical tests, you may upload a single table file with tests, Ns, etc., with reference to sections in the manuscript.)

Group allocation

- Indicate how samples were allocated into experimental groups (in the case of clinical studies, please specify allocation to treatment method); if randomization was used, please also state if restricted randomization was applied
- Indicate if masking was used during group allocation, data collection and/or data analysis

Please outline where this information can be found within the submission (e.g., sections or figure legends), or explain why this information doesn't apply to your submission:

Group allocation does not apply to this study, other than in the most obvious sense:
- plants of a specific genotype are treated as a group
- for iCLIP experiments, protein-RNA complexes migrating around 55 kDa are treated as one sub-group, while protein-RNA complexes migrating at or above 110 kDa are treated as a separate group.
This information is detailed both in Results and in Methods.

Additional data files ("source data")

- We encourage you to upload relevant additional data files, such as numerical data that are represented as a graph in a figure, or as a summary table
- Where provided, these should be in the most useful format, and they can be uploaded as "Source data" files linked to a main figure or table
- Include model definition files including the full list of parameters used
- Include code used for data analysis (e.g., R, MatLab)
- Avoid stating that data files are "available upon request"

Please indicate the figures or tables for which source data files have been provided:

eLife Sciences Publications, Ltd is a limited liability non-profit non-stock corporation incorporated in the State of Delaware, USA, with company number 5030732, and is registered in the UK with company number FC030576 and branch number BR015634 at the address 1st Floor, 24 Hills Road, Cambridge CB2 1JP | August 2014

Code is available at
https://github.com/sarah-ku/targets_arabidopsis

Figure 1 and Figure 1 supplement 1:
HyperTRIBE sequencing data is available at European Nucleotide Archive (accession number PRJEB44359).
Full gel images (Figure1–figure supplement 1A) have been uploaded as source data to eLife.

Figure 2
HyperTRIBE sequencing data is available at European Nucleotide Archive (accession number PRJEB44359).

Figure 3, Figure 3 supplement 1, Figure 3 supplement 3
iCLIP-seq data is available at European Nucleotide Archive (accession number PRJEB44359).

Full gel images (Figure 3B,D; Figure 3–figure supplement 1A-E; Figure 3–figure supplement 3A-C) have been uploaded as source data to eLife.

Figures 4-7
iCLIP-seq data is available at European Nucleotide Archive (accession number PRJEB44359).
Coordinates of m6A-sites and ECT2 FA-CLIP sites from previously published datasets on m6A-sites were obtained from public repositories as indicated in figure legends.
