# Supplementary material for: Principles of mRNA targeting via the Arabidopsis m6A-binding protein ECT2
Source: eLife. 2021 Sep 30;10:e72375. doi: 10.7554/eLife.72375 (PMC8796052; doi:10.7554/eLife.72375)
Supplement: Figure 5—figure supplement 4—source data 1. [file elife-72375-fig5-figsupp4-data1.pdf]

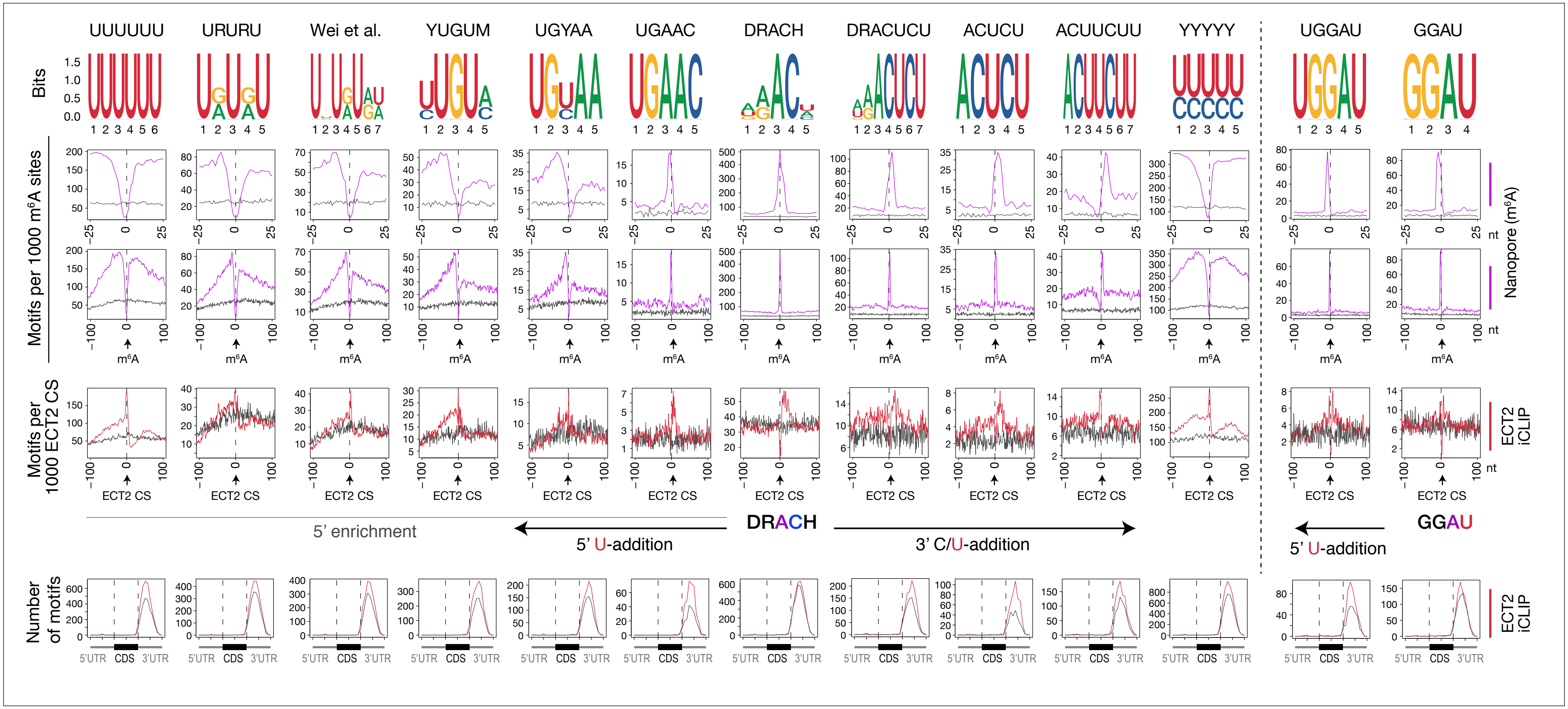

Figure 5—figure supplement 4. Uridines flanking DRACH result in additional motifs enriched at ECT2 iCLIP sites. Enrichment of DRACH-like motifs containing additive amounts of U(/Y)s at the flanks until there is only U/Ys. Through the upstream U-additions, transition forms like URURU/UGUAY/YUGUM are included. GGAU with addition of one U upstream is also shown on the right side of the figure. From top panel to bottom: (1) motif logos for derived position weight matrices (PWMs); (2-4) distribution of the relevant motif relative to m⁶A-Nanopore (Parker et al., 2020) (2,3) or ECT2–iCLIP crosslink sites (CS) (4). Grey lines represent the distribution for gene-body location-matched background set of sites of equivalent number; (5) total number of the relevant motif found at ECT2-iCLIP sites according to gene body location. Grey lines indicate numbers found in the same gene-body location-matched background set.
